# Cognition and metacognition in functional motor symptoms and functional seizures: a case–control study

**DOI:** 10.1017/S0033291726103420

**Published:** 2026-03-03

**Authors:** Susannah Pick, L.S. Merritt Millman, Esin Gun Gürsoy, Yasmine Basamh, Jemima Uloyok-Job, Jessica Davies, Lauren Blunstone, Snigdha Bhuma, Jan Coebergh, Anthony S. David, Mark J. Edwards, Laura H. Goldstein, John Hodsoll, Mitul A. Mehta, Timothy R. Nicholson, Biba Stanton, Joel S. Winston, Matthew Hotopf, Trudie Chalder

**Affiliations:** 1 Psychological Medicine, King’s College London, Institute of Psychiatry Psychology & Neuroscience, London, UK; 2 South London and Maudsley Mental Health NHS Trust, UK; 3 St George’s University Hospitals NHS Foundation Trust, UK; 4 Institute of Mental Health, University College London, UK; 5 Basic & Clinical Neurosciences, King’s College London, Institute of Psychiatry Psychology & Neuroscience, UK; 6 Psychology, King’s College London, Institute of Psychiatry Psychology & Neuroscience, UK; 7 Biostatistics & Health Informatics, King’s College London, Institute of Psychiatry Psychology & Neuroscience, UK; 8 Neuroimaging, King’s College London, Institute of Psychiatry Psychology & Neuroscience, UK; 9 Psychosis Studies, King’s College London, Institute of Psychiatry Psychology & Neuroscience, UK; 10 King’s College Hospital NHS Foundation Trust, UK

**Keywords:** attention, cognitive, conversion disorder, dissociative seizures, executive, functional motor, functional movement disorder, functional seizures, metacognition, neuropsychology, non-epileptic seizures, performance validity

## Abstract

**Background:**

Cognitive symptoms are common in functional neurological disorder (FND), yet evidence of impaired neurocognitive test performance is variable. We aimed to assess self-reported cognitive symptoms, neurocognitive test performance, and metacognitive confidence in patients with functional seizures (FS) and functional motor symptoms (FMS).

**Methods:**

Participants with FS (n = 50) and FMS (n = 50) were compared to age- and gender-matched healthy controls (HC, n = 50), and clinical controls with depression and/or anxiety disorders (CC, n = 50). The Cambridge Neuropsychological Test Automated Battery was used to examine response speed, working memory, executive functions, and social–emotional processing, with subjective confidence rated for each test. Intellectual functioning, performance validity, and self-reported cognitive symptoms were also assessed.

**Results:**

The FND groups reported elevated cognitive symptoms compared to HC and CC (p-values<0.001). Impaired performance was demonstrated in both FND groups on tests of sustained attention (p-values = 0.03- < 0.001) and set-shifting (p-values = 0.01–0.001). Performance validity was comparable between groups (p = 0.64). The FND groups reported reduced post-diction confidence for sustained attention (p < 0.001). Executive performance deficits correlated with reduced test-specific confidence in FS/FMS (p-values = 0.02- < 0.001). In FMS, post-diction confidence for sustained attention performance correlated negatively with cognitive symptoms (p = 0.002). Cognitive symptoms were associated with psychological/physical symptom load, quality-of-life, and/or general functioning in FND and CC groups (p-values = 0.04- < 0.001).

**Conclusions:**

Patients with FS and FMS displayed localized deficits on tests of executive functioning, with reduced domain-specific metacognitive confidence, alongside significant cognitive symptoms. These neurocognitive features were associated with poorer clinical status, warranting interventions targeting cognitive control and/or cognitive symptoms in everyday life.

## Introduction

Cognitive symptoms, such as forgetfulness, distractibility, and disrupted goal-driven behaviours, are commonly reported by patients with functional neurological disorder (FND) (APA, 2013; Butler et al., 2021; Pick et al., 2023; Teodoro, Edwards, & Isaacs, 2018). Occurring as the primary concern in functional cognitive disorder (FCD) (Teodoro et al., 2018), or alongside other FND presentations such as functional seizures (FS) and functional motor symptoms (FMS) (Pick et al., 2023; Teodoro et al., 2018), self-reported cognitive symptoms have been associated with greater psychological symptom load and compromised quality of life (Forejtova et al., 2023; Millman, Williams, Jungilligens, & Pick, 2025; Pick et al., 2023; Vechetova et al., 2018).

Despite the prevalence and apparent impact of self-reported cognitive difficulties in FND, neuropsychological testing studies have provided inconsistent evidence for cognitive performance deficits across FND phenotypes. One systematic review and meta-analysis reported significant performance impairments across cognitive domains in patients with FS, with moderate-large effect sizes compared to healthy controls (Van Patten et al., 2024a). Compromised cognitive performance may also be related to poorer mental health and quality-of-life in FS (Van Patten et al., 2024b). However, patients with FS displayed superior performance to epilepsy controls on tests of general cognitive functioning, language, and long-term memory (Millman et al., 2025; Van Patten et al., 2024a). The broader neuropsychological literature across FND phenotypes has also provided mixed evidence for intact, impaired, or superior cognitive test performance compared to neurological, psychiatric, and/or healthy controls (Alluri et al., 2020; Millman et al., 2025; Pick et al., 2023; Teodoro et al., 2018; Van Patten et al., 2024a). While a considerable number of investigations have examined cognitive performance in FS samples, fewer have focused on FMS, FCD, or mixed FND presentations (Millman et al., 2025; Van Patten et al., 2024a). It therefore remains unclear whether cognitive performance impairments are present to a similar extent across FND phenotypes, and which cognitive domains are most affected.

Beyond core cognitive functions, emerging findings indicate potential differences in metacognition in FND (Sadnicka et al., 2025; Teodoro et al., 2018). Metacognition refers to awareness and control of one’s own cognitive processes, and adaptive use of this knowledge in goal-driven behaviours (Fleming, 2024). Metacognitive deficits are associated transdiagnostically with mental health symptoms and maladaptive behaviour, and may be important treatment targets in neuropsychiatric disorders (Seow, Rouault, Gillan, & Fleming, 2021). ‘Global metacognition’ involves broad self-evaluations of cognitive performance, often assessed with self-report measures of cognitive symptoms or aspects of cognitive functioning in everyday life. ‘Local metacognition’, on the other hand, relates to shorter-term assessments of performance or specific decisions/actions within a particular cognitive task/activity (Sadnicka et al., 2025; Seow et al., 2021). In FND, altered global metacognition has been indicated by the presence of elevated self-reported cognitive symptoms and/or poorer self-evaluations of everyday cognitive functioning, in the absence of observable performance deficits (Alluri et al., 2020; Pick et al., 2023; Sadnicka et al., 2025; Teodoro et al., 2018). Reduced concordance between neurocognitive test performance and post-diction self-evaluations in some FND samples has also indicated potentially diminished local metacognition (Millman et al., 2025; Pick et al., 2023; Sadnicka et al., 2025; Teodoro et al., 2018), although a limited number of performance-controlled experimental studies have yielded mixed findings on local metacognition in FND samples to date (Sadnicka et al., 2025).

The disparate findings relating to cognitive functioning in FND may be due in part to methodological issues, such as suboptimal consideration of confounding variables (e.g., age, education, medication, and performance validity), the wide range and variable quality of measures employed, and differing comparison groups (Millman et al., 2025; Pick et al., 2023; Sadnicka et al., 2025; Van Patten et al., 2024a,2024b). As such, studies with improved methodological rigor are needed to establish with greater certainty whether cognitive and metacognitive differences are related directly to the presence of FND, rather than to non-specific factors that are merely associated with the disorder. Additionally, few studies have directly compared cognitive and metacognitive profiles in specific FND subgroups using the same measures, which could highlight shared and distinct cognitive/metacognitive features, with clinical and mechanistic relevance.

### Aims and hypotheses

Within a larger research program examining aetiological factors and mechanisms specifically in FS and FMS (Pick et al., 2024), this study aimed to assess self-reported cognitive symptoms, neuropsychological test performance profiles, and post-diction metacognitive confidence in patients with a primary diagnosis of FS or FMS. We sought to compare the FS and FMS groups to healthy controls without physical or mental health disorders (HC), and clinical controls (CC) with major depression and/or anxiety disorders, to better understand the neurocognitive features that distinguish those with FS and FMS from the general population, and from those with common mental health symptoms. This project was not designed to investigate mechanisms in FCD, but rather to improve the understanding of cognitive and metacognitive functioning in FS and FMS – two of the most common FND presentations.

The objectives were to examine overall between-group differences (FS/FMS/CC/HC) and FND subgroup-specific (FS/FMS) outcome profiles in cognitive domains with clinical and mechanistic relevance in FND (Brown & Reuber, 2016; Drane et al., 2020; Edwards et al., 2012; Pick, Goldstein, Perez, & Nicholson, 2019), including (1) attention, executive functioning, working memory, and social–emotional processing test performance, (2) test-specific post-diction metacognitive confidence, and (3) self-reported cognitive symptoms. We also sought to explore within-group relationships between cognitive/metacognitive outcomes and clinical characteristics (FS/FMS/CC).

Compared to HC and CC, we predicted that the FS and/or FMS groups would display:

- Elevated self-reported cognitive symptoms (Butler et al., 2021; Pick et al., 2023).
- Altered performance on tests of:
  - executive functioning (i.e., diminished sustained attention, set-shifting, response inhibition) (Alluri et al., 2020; Brown & Reuber, 2016; Edwards et al., 2012; Millman et al., 2025; Pick et al., 2019; Van Patten et al., 2024a);
  - working memory (poorer performance) (Alluri et al., 2020; Jungilligens et al., 2022; Millman et al., 2025; Pick et al., 2023; Van Patten et al., 2024a);
  - social–emotional processing (enhanced emotional bias/reduced emotion recognition) (Brown & Reuber, 2016; Pick et al., 2019);
- Reduced post-diction metacognitive confidence (Sadnicka et al., 2025).

## Methods

### Study design and participants

This single-centre, case–control study was based at King’s College London (KCL), conducted within a larger research program (Pick et al., 2024). Ethical approval was granted by the North-West Greater Manchester South National Health Service (NHS) Research Ethics Committee, United Kingdom (UK, Reference: IRAS 322652). Recruitment and data collection proceeded from 11/2023 to 04/2025.

Participants with FMS (n = 50) or FS (n = 50) were recruited from neuropsychiatry/neurology services at King’s College Hospital, South London, and Maudsley, and St George’s University Hospital NHS Foundation Trusts (London, UK). Most of the FS (82%) and FMS (96%) groups self-referred to the study following adverts shared by clinicians and/or through FND Hope UK. When self-referring to the study, participants in the FND groups were asked to provide documentary evidence of an FND diagnosis from a specialist clinician. Healthy (n = 50) and clinical (n = 50) controls were recruited using advertisements on community webpages and participant registers. Control participants were selected to frequency-match groups for age and gender (1:1 cases/controls). An a-priori power calculation indicated that a between-group (FS/FMS/CC/HC) ANOVA/ANCOVA, used to test the primary hypotheses, would require a sample of 180 to achieve 80% power for the detection of a small effect (f = 0.1, p < 0.05).

Inclusion criteria required that participants were aged 18–65 years, fluent in English, and had normal/corrected eyesight. FND samples were required to have a confirmed DSM-5 (Association, 2013) diagnosis with FS *or* FMS as their most prominent symptom. Clinical controls met criteria for a diagnosis of major depression and/or an anxiety disorder, confirmed with a structured clinical interview (‘Materials and Measures’). We excluded candidates with active severe psychiatric disorder (i.e., severe alcohol/substance use disorder, psychosis) and current major neurological diseases and/or lesions (e.g., neurodegenerative disorder, active epilepsy). Other exclusion criteria were being unable to perform tasks due to significant physical/cognitive impairment, current participation in an interventional trial, lifetime diagnosis of FND (HC/CC), or the presence of concurrent FS and FMS within the previous 12 months (FND). Individuals with a primary diagnosis of FCD were ineligible.

## Materials and measures

### Clinical measures (interview, self-report questionnaires)

A medical history interview was conducted at baseline (SP/LSMM/SB), including modules from the *Quick Structured Clinical Interview for DSM-5 Disorders* (First & First, 2021) (Psychosis, Alcohol/Substance Use Disorder, Affective Disorders, Anxiety Disorders). Clinical self-report questionnaires (Supplementary Table 1) assessed depression, generalised anxiety, psychological/somatoform dissociation, physical symptoms, health-related quality-of-life, and general functioning (Pick et al., 2024). A study-specific scale was developed to quantify FND symptom severity/impact (Supplementary Table 2).

The *Cognitive Failures Questionnaire* (*CFQ*) (Broadbent, Cooper, FitzGerald, & Parkes, 1982) assessed self-reported cognitive symptoms over the preceding 6 months, such as everyday memory and attention lapses, and disrupted action sequences. Higher scores signify poorer subjective cognitive functioning. Satisfactory psychometric properties have been demonstrated for this measure (Bridger, Johnsen, & Brasher, 2013; Broadbent et al., 1982; Wallace, Kass, & Stanny, 2002).

### Medical Symptom Validity Test (MSVT)

The *MSVT* is a computerised test designed to examine neuropsychological test performance validity, with sound psychometric properties (Green, 2005; Green & Flaro, 2016; Green, Montijo, & Brockhaus, 2011). Performance validity indicators are *Immediate* and *Delayed Recall* scores, and immediate-delayed *Consistency* scores (test failure indicated by scores ≤85%). This test was included to assess task engagement/negative response biases across groups (McWhirter, Ritchie, Stone, & Carson, 2020). A pooled failure base-rate of 33% has been reported in previous FS samples (Roor, Peters, Dandachi-FitzGerald, & Ponds, 2024).

### Wechsler Abbreviated Scale of Intelligence – 2nd edition (WASI-II)

The *WASI-II* two-subtest version was administered to test non-verbal and verbal intellectual abilities (*Matrix Reasoning/Vocabulary*) and to provide an estimated *Full-Scale Intellectual Quotient Score (WASI-II FSIQ-2).* This version has strong inter-rater reliability (.95–.99), internal consistency (0.94), and test–retest stability (.94) (Wechsler, 2011).

### Cambridge Neuropsychological Test Automated Battery (CANTAB) Connect

The *CANTAB Connect* platform is a modified version of the original battery, administered using a touchscreen device. Selected subtests probed motor/cognitive response times, attention, executive functioning, social–emotional cognition, and memory (Table 1, Supplementary Table 3). The original battery has strong psychometric properties in clinical and non-clinical samples (Robbins et al., 1994, 1998), although psychometric data for the *Connect* version are not currently available.

**Table 1.** CANTAB Connect subtests

Subtest	Domain	Description
Motor Screening (2 min)	Sensorimotor speed	Trials consist of a colored cross presented onscreen in an unpredictable spatial location. The location of the cross varies between trials. Participants are asked to manually tap the cross as fast as possible. *Outcome variables*: Total Correct/Incorrect; Motor Mean Latency
Reaction Time (3 min)	Motor and cognitive response speed	A circle is presented at the bottom of the screen, with five circles at the top. One of the top circles lights up on each trial. Participants must press on the bottom circle until the top circle lights up, then manually tap the illuminated upper circle as quickly as possible. *Outcome variables*: Total Error Score; Movement Time; Reaction Time
Rapid Visual Information Processing (~7 min)	Sustained attention	A series of digits is presented individually onscreen at a rate of 100 digits/minute. Participants are asked to identify target sequences of digits (e.g., 6–2–9) by manually tapping a button onscreen as fast as possible. *Outcome variables*: Ability score; Total Misses; Probability of Hit; False Alarm; Response Latency
Spatial Span (5 min)	Visuospatial working memory	Sequences of squares are shown changing color individually, with an unpredictable spatial pattern. On each trial, participants are asked to tap the squares in the sequence that changed color. The Forward version requires the sequence to be repeated in the same order. The Reverse version requires the sequence to be repeated backwards. The number of squares increases during the task from 2 to 9, increasing the difficulty level. *Outcome variables*: Forward/Reverse errors; Forward/Reverse Span Length
Intra-Extra Dimensional Set Shift (7 min)	Executive functioning: Attentional set-shifting & cognitive flexibility	Participants aim to identify an implicit rule based on an array of pink shapes and white lines. Participants manually select the stimulus that they think aligns with the current rule. Auditory feedback indicates ‘Correct’ or ‘Incorrect’ responses. After six correct responses, the rule changes, and the participant attempts to identify the new rule. Rule changes can be within a dimension (intra-dimensional set shift) or shift to a different dimension (extra-dimensional set shift). Difficulty increases across the trials. *Outcome variables*: Total Trials/Stages; Total/Adjusted Errors; Extra-Dimensional Shift Errors; Pre-Extra-Dimensional Shift Errors; Completed Stage Trials/Errors; Response Latency
Stop Signal Task (14 min)	Executive functioning: Response inhibition	Trials consist of an arrow presented centrally at the top of the screen, pointing to the right or left, with a right and left button presented below on the respective side. Participants respond by manually tapping the right or left button, based on the direction of the arrow. On some trials, a tone is presented. Participants are asked to withhold their response when they hear the tone (response inhibition). The stop-signal delay is variable and adapts to the participant’s performance. *Outcome variables*: Missed Trials; Errors (Go/Stop trials); Stop Signal Reaction Time
Emotional Bias Task (4 min)	Social Cognition (Emotional processing)	Trials consist of a briefly presented morphed emotional face (150 msec). The face varies in intensity between two emotions (e.g., happy–angry). Participants respond by selecting which of the two emotions they perceived (e.g., happiness or anger). Three versions of the task were administered (happy–anger, happy–disgust, happy–sadness). *Outcome variables*: Reaction Time; Bias Point – Proportion of Happy selections.
Emotion Recognition Test (6–10 min)	Social cognition (Emotional processing)	Trials consist of a briefly presented emotional face (200 msec), displaying anger, sadness, fear, disgust, surprise, or happiness. Participants respond by manually tapping the emotion they perceived from an array of six emotion labels. *Outcome variables*: Reaction Times; False Alarms; Total/Unbiased Hits

Adapted from Pick et al. (2023).

### Metacognitive confidence ratings

Post-diction metacognitive confidence ratings were obtained immediately after every test (1 = Very poor performance; 2 = Poor performance; 3 = Below average; 4 = Average; 5 = Above average; 6 = Superior; 7 = Very superior). Such metacognitive assessments have been used widely in other clinical disorders (Seow et al., 2021), capturing short-term, test-level metacognitive judgements.

## Procedure

The recruitment and screening process is detailed in the study protocol (Pick et al., 2024). After obtaining written consent, the medical history interview was conducted remotely. Eligible participants were sent a link to the online questionnaires to be completed within 48 hours of the research visit. The cognitive battery was administered in a quiet testing room, between 10 am and 12 pm for most participants. The session commenced with the *WASI-II*, followed by the *CANTAB* battery (fixed order, Table 1), then the *MSVT.* Participants were provided with standardised instructions for each test. Abstinence from caffeine/nicotine for >2 hours before the visit was requested of all participants. Those on psychotropic medications that might alter cognition were asked to abstain for 12–24 hours, provided abstinence was unlikely to cause adverse consequences (self-report/judgement of principal investigator/research group). Participants received a £50 shopping voucher on completion of the session.

## Data processing and statistical analyses

Data analyses were conducted in SPSS (v29, IBM, 2022) by SP and EGG independently, following a pre-registered analysis plan (https://osf.io/grh89/). Assumptions were checked for each test, and if violated, suitable corrections were implemented (‘Results’). We defined outliers as scores of 2.5 standard deviations (+/−) from the group mean for each variable. Analyses were rerun with outliers winsorised in sensitivity analyses. Some participants failed to complete tests due to technicalities, symptoms, or declining (‘Results’). There were no within-test missing data points because each test required all trials/elements to be completed to yield a valid score.

Categorical variables were examined with Fisher’s exact/chi-squared tests. One-way Analysis of Variance (ANOVA) was used for between-group comparisons with most continuous variables. Mixed-model ANOVA was used for the *CANTAB Emotion Recognition Test*, with group as the between-groups factor (FMS/FS/CC/HC) and emotion (happiness, disgust, anger, fear, sadness, surprise) as the within-subjects factor. Significant main effects/interactions were examined with Bonferroni or Games–Howell post hoc tests for ANOVA/Welch’s ANOVA. Cramer’s V, eta-squared, partial eta-squared, and omega-squared were calculated as effect sizes, as appropriate. Analysis of Covariance (ANCOVA) was applied in sensitivity analyses to examine the influence of potentially relevant confounding variables, such as performance validity, education, intellectual functioning, age, gender, and psychotropic medication. Results that did not withstand sensitivity analyses were considered uninterpretable and not considered further.

Exploratory Spearman’s correlations were run between variables that differed significantly between groups. Benjamini–Hochberg corrections were applied within each set of tests to control the false discovery rate (5%). Results remaining significant after correction are reported.

## Results

### Sample characteristics

Sociodemographic and clinical characteristics of the 200 participants are presented in Table 2, including common psychotropic medications and comorbid diagnoses (additional details provided in Supplementary Tables 4–5). The groups were comparable in age, gender, and handedness. Significant overall group differences were found for self-reported education, psychotropic medication, comorbid physical/mental health diagnoses, anxiety, depression, somatoform and psychological dissociation, physical symptoms, health-related quality-of-life, and work/social functioning. These results largely reflected expected differences between HC and FS/FMS/CC groups (https://osf.io/preprints/psyarxiv/8vtpe_v1). The CC and FND groups were similar in rates of physical health comorbidities (p = 0.25), anxiety (p = 0.55), and depression severity (p = 0.50), but they differed significantly in education (p = 0.01) and psychotropic medication use (p < 0.001).

**Table 2.** Sample characteristics, cognitive symptoms, performance validity, and intellectual functioning

Characteristics	FS (n = 50)	FMS (n = 50)	CC (n = 50)	HC (n = 50)	Comparison statistics
Age (years): M (SD)	36.7 (13.25)	35.9 (11.1)	37.4 (13.1)	37.7 (14.9)	F(3,196) = 0.18, p = 0.91, η^2^ = 0.003
Gender: n (%)	F = 39 (78) M = 9 (18) Other = 2 (4)	F = 41 (82) M = 9 (18) Other = 0 (0)	F = 42 (84) M = 7 (14) Other = 1 (2)	F = 41 (82) M = 9 (18) Other = 0 (0)	Fisher’s exact = 3.39, p = 0.81, v = 0.10
Handedness (self-reported): n (%)	R = 40 (80) L = 4 (8) A = 6 (12)	R = 45 (90) L = 2 (4) A = 3 (6)	R = 42 (84) L = 0 (0) A = 8 (16)	R = 46 (92) L = 0 (0) A = 4 (8)	Fisher’s exact = 9.07, p = 0.12, v = 0.16
Education: n (%)	C = 9 (18) AL = 15 (30) UG = 11 (22) PG = 15 (30)	C = 7 (14) AL = 19 (38) UG = 17 (34) PG = 7 (14)	C = 3 (6) AL = 8 (16) UG = 25 (50) PG = 14 (28)	C = 2 (4) AL = 11 (22) UG = 16 (32) PG = 21(42)	χ^2^ (9, n = 200) = 24.20, **p = 0.004**, v = 0.20
Current physical health diagnosis: n (%)	Y = 32 (64) N = 18 (36)	Y = 36 (72) N = 14 (28)	Y = 28 (56) N = 42 (44)	Y = 15 (30) N = 35 (70)	χ^2^ (3, n = 200) = 20.14, **p < 0.001**, v = 0.32
Current (self-reported) mental health diagnosis: n (%)	Y = 42 (84) N = 8 (16)	Y = 34 (68) N = 16 (32)	Y = 50 (100) N = 0 (0)	Y = 0 (0) N = 50 (100)	Fisher’s exact = 147.75, **p < 0.001**, v = 0.79
Current psychotropic medication^a^: n (%)	None = 11 (22) Low = 5 (10) Mod = 29 (58) High = 5 (10)	None = 12 (24) Low = 3 (6) Mod = 29 (58) High = 6 (12)	None = 22 (44) Low = 0 (0) Mod = 27 (54) High = 1 (2)	None = 50 (100)	Fisher’s exact = 8.93, **p < 0.001**, v = 0.38
Anti-seizure (e.g., pregabalin, lamotrigine)	16 (32%)	15 (30%)	3 (6%)	0 (%)	
Antidepressants (e.g., SSRI, SNRI, tricyclic)	43 (86%)	42 (84%)	26 (52%)	0 (%)	
Benzodiazepines (e.g., diazepam)	5 (10%)	2 (4%)	3 (6%)	0 (%)	
Central nervous system stimulants (e.g., methylphenidate)	4 (8%)	0 (%)	2 (4%)	0 (%)	
Opiates (e.g., tramadol, co-codamol)	5 (10%)	13 (26%)	3 (6%)	0 (%)	
CFQ Total: M (SD) (FS = 49; CC = 48)^b^	57.25 (17.05)	57.26 (16.97)	45.54 (13.87)	31.44 (14.93)	F(3,193) = 30.10, **p < 0.001**, η^2^ = 0.32
MSVT					
MSVT Immediate Recall % Correct: M (SD)	98.60 (4.85)	98.30 (4.24)	97.70 (14.15)	98.90 (4.44)	F(3,196) = 0.20, p = 0.90, η^2^ = 0.00
MSVT Delayed Recall % Correct: M (SD) (FS = 49)^b^	98.78 (5.16)	96.60 (8.72)	99.20 (2.55)	98.40 (6.50)	^c^F(3,97.51) = 1.48, p = 0.23, ω^2^ = 0.01
MSVT Consistency %: M (SD) (FS = 48)^b^	98.13 (5.89)	96.30 (8.32)	97.30 (13.49)	97.90 (6.71)	F(3,194) = 0.40, p = 0.76, η^2^ = 0.01
MSVT Pass rate: n (%)	48 (96)	46 (92)	48 (96)	49 (98)	Fisher’s exact = 2.02, p = 0.64, v = 0.11
WASI-II					
WASI-II FSIQ–2: M (SD)	96.80 (14.66)	97.74 (14.12)	108.04 (11.63)	105.36 (10.46)	^c^F(3,107.96) = 9.15, **p < 0.001**, ω^2^ = 0.11
WASI-II Vocabulary T Score: M (SD)	45.94 (9.55)	47.98 (11.85)	52.30 (9.21)	49.78 (7.12)	F(3,196) = 3.98, **p = 0.009**, η^2^ = 0.06
WASI-II Matrix Reasoning T Score: M (SD)	50.42 (9.81)	49.46 (9.00)	56.54 (8.25)	56.56 (8.87)	F(3,196) = 9.09, **p < 0.001**, η^2^ = 0.12

*Note:* A, ambidextrous; AL, A levels or equivalent; C, compulsory; CC, clinical control; CFQ, Cognitive Failures Questionnaire; CGI, Clinical Global Impression; FMS, functional motor symptoms; FS, functional seizures; HC, healthy control; L, left; M, mean; Mod, Moderate; MSVT, Medical Symptom Validity Test; N, no; NA, not applicable; O, other; PG, postgraduate degree; R, right; SD, standard deviation; SNRI, selective serotonin and noradrenalin reuptake inhibitors; SSRI, selective serotonin reuptake inhibitors; UG, undergraduate degree; WASI-II FSIQ-2, Wechsler Abbreviated Scale of Intelligence–Full-Scale Intelligence Quotient 2 sub-test; Y, yes. Bold values indicate statistically significant (p < 0.05).

a Low, ‘Occasional use/very low daily dose 1–2 psychotropics’; Moderate, ‘medium-high (therapeutic) doses of 1–3 psychotropics (daily/multiple daily)’; High, ‘high-very high doses of 3 or more psychotropics (daily/multiple daily/continuous)’.

b Sample size deviated due to missing data.

c Welch’s ANOVA.

### Cognitive symptoms

**Figure 1.**
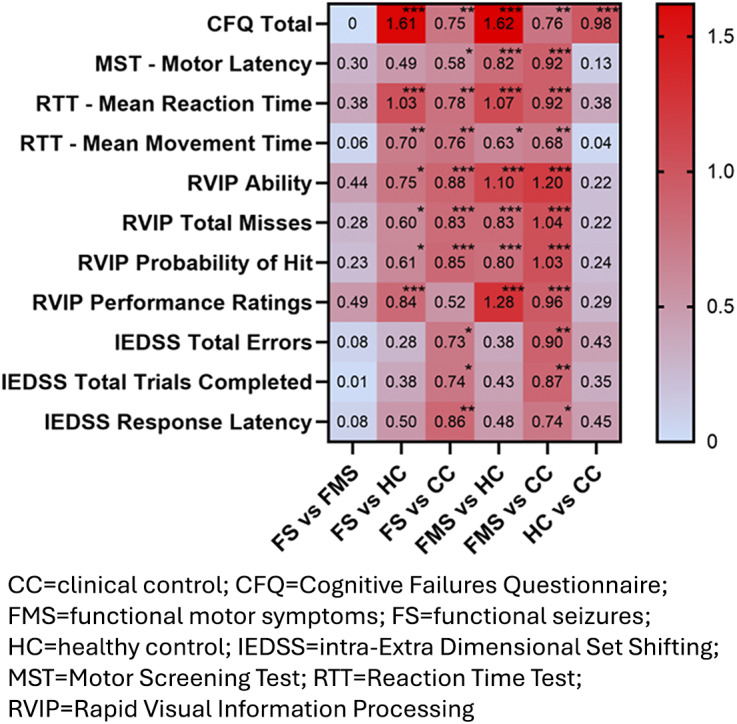
Cohen’s d (effect size) for cognitive symptoms (CFQ), Motor/Reaction Time (MST/RTT), Sustained Attention (RVIP), and Set-Shifting (IEDSS) tasks.

Self-reported cognitive symptoms (*CFQ-Total*) differed significantly between groups (Table 2, Figure 1), withstanding sensitivity analyses (Supplementary Table 6). Bonferroni post hoc tests (Supplementary Table 7) showed that *CFQ-Total* scores were elevated in the FND groups compared to HC and CC, and they were also higher in the CC group relative to HC (p-values = 0.002- < 0.001).

### Performance validity

The *MSVT* performance validity test *Pass* rate was comparable between groups, and there were no significant differences between groups in *Immediate Recall, Delayed Recall*, and *Consistency* scores (Table 2).

### General intellectual functioning

On the *WASI-II* test (Table 2), overall intellectual functioning scores (*FSIQ-2*) were in the average range in all groups. There were significant differences between groups for *FSIQ-2, Vocabulary*, and *Matrix Reasoning* scores. Pairwise comparisons (Supplementary Table 8) indicated that performance was poorer in the FS group than in CC for *FSIQ-2* (p = 0.005) and *Vocabulary* (p = 0.006). Both FS and FMS groups showed reduced performance on *Matrix Reasoning* compared to HC and CC (p-values = 0.005- < 0.01). The group differences in *WASI-II* performance survived all sensitivity analyses (Supplementary Table 9).

### CANTAB test performance

Statistical values for the *CANTAB* performance outcomes that varied between groups are shown in Table 3 and Figure 1.

**Table 3.** CANTAB results – Motor/reaction time, sustained attention, and set-shifting

	FS	FMS	CC	HC	ANOVA statistics
Motor Screening Test (MST)	n = 50	n = 50	n = 50	n = 50	
MST – Mean Motor Latency (ms): M (SD)	913.55 (327.33)	1016.04 (351.87)	760.12 (178.29)	783.94 (186.69)	F(3,105.30) = 8.88, **p < 0.001**, ω^2^ = 0.11
Reaction Time Test (RTT)	n = 50	n = 50	n = 50	n = 50	
RTT – Mean Reaction Time (ms): M (SD)	421.90 (84.15)	466.16 (141.35)	370.32 (40.71)	356.43 (32.10)	F(3,100.98) = 16.81, **p < 0.001**, ω^2^ = 0.20
RTT – Mean Movement Time (ms): M (SD)	297.05 (85.52)	292.01 (86.38)	243.95 (50.43)	246.12 (57.23)	F(3,106.39) = 7.96, **p < 0.001**, ω^2^ = 0.10
Rapid Visual Information Processing (RVIP)	n = 47	n = 50	n = 49	n = 49	
RVIP Mdn Response Latency (ms): M (SD)	466.40 (92.40)	543.07 (177.03)	453.02 (80.91)	457.07 (75.48)	^a^F(3,103.87) = 3.73, **p = 0.01**, ω^2^ = 0.08
RVIP Ability: M (SD)	0.89 (0.04)	0.87 (0.05)	0.93 (0.05)	0.92 (0.04)	F(3, 191) = 13.40, **p < 0.001**, η^2^ = 0.17
RVIP Total Misses: M (SD)	22.45 (8.55)	25.04 (9.88)	15.06 (9.28)	17.10 (9.34)	F(3, 191) = 12.22, **p < 0.001**, η^2^ = 0.16
RVIP Probability of Hit: M (SD)	0.58 (0.16)	0.54 (0.18)	0.72 (0.17)	0.68 (0.17)	F(3, 191) = 12.22, **p < 0.001**, η^2^ = 0.16
RVIP Probability of False Alarm: M (SD)	0.01 (0.03)	0.02 (0.04)	0.01 (0.01)	0.01 (0.05)	F(3, 191) = 0.82, p = 0.49, η^2^ = 0.01
Intra-Extra Dimensional Set-Shift (IEDSS)	n = 50	n = 50	n = 50	n = 50	
IEDSS Total Errors: M (SD)	18.20 (11.79)	19.05 (10.65)	11.84 (4.36)	15.14 (10.02)	^a^F(3, 79.94) = 7.99, **p < 0.001**, ω^2^ = 0.07
IEDSS Adjusted Errors: M (SD)	39.76 (49.35)	32.80 (30.52)	20.14 (22.77)	27.28 (39.07)	^a^F(3,105.13) = 3.18, **p = 0.03**, ω^2^ = 0.02
IEDSS Extra-Dimensional Shift Errors: M (SD)	5.81 (6.13)	8.91 (8.93)	7.02 (8.14)	7.82 (9.89)	^a^F(3,100.54) = 1.31, p = 0.28, ω^2^ = 0.00
IEDSS Pre-Extra-Dimensional Shift Errors: M (SD)	13.32 (11.03)	12.90 (11.92)	7.72 (6.90)	10.42 (9.13)	^a^F(3,106.45) = 4.29, **p = 0.007**, ω^2^ = 0.04
IEDSS Stages Completed: M (SD)	8.04 (2.08)	8.36 (1.21)	8.68 (0.84)	8.46 (1.61)	^a^F(3,103.78) = 1.78, p = 0.16, ω^2^ = 0.01
IEDSS Total Trials Completed: M (SD)	85.80 (23.98)	86.05 (20.16)	72.53 (9.54)	77.75 (18.47)	^a^F(3, 82.04) = 7.34, **p < 0.001**, ω^2^ = 0.07
IEDSS Completed Stage Trials: M (SD)	74.88 (29.61)	77.82 (18.95)	70.92 (11.75)	71.90 (20.95)	^a^F(3,103.42) = 1.69, p = 0.17, ω^2^ = 0.00
IEDSS Response Latency (ms): M (SD)	131119.23 (56928.56)	136710.59 (81216.06)	91660.98 (32183.23)	107135.32 (37080.14)	^a^F(3, 84.39) = 7.18, **p < 0.001**, ω^2^ = 0.09

*Note:* CC, ‘clinical controls’; FS, ‘functional seizures’; FMS, ‘functional motor symptoms’; HC, ‘healthy controls’; IEDSS = Intra-Extra Dimensional Set-Shift’; M, ‘mean’; ms, ‘milliseconds’; MST, ‘Motor Screening Test’; RTT, ‘Reaction Time Test’; RVIP, ‘Rapid Visual Information Processing’; SD, ‘standard deviation’. Bold values indicate statistically significant (p < 0.05).

a Welch’s ANOVA.

*CANTAB Motor Screening Test (MST) – Mean Motor Latency* diverged significantly between groups (Table 3), remaining so in all sensitivity analyses (Supplementary Table 10). Games–Howell post hoc tests (Supplementary Table 11) showed extended latency in the FMS group compared to both HC (p < 0.001) and CC groups (p < 0.001), and in FS relative to CCs (p = 0.02).

*CANTAB Reaction Time Test (RTT*) – *Mean Reaction Time* and *Mean Movement Time* scores were significantly different across groups (Table 3). Games–Howell pairwise tests (Supplementary Table 11) demonstrated significantly elevated scores (slower responses) in both FND groups compared to HC/CC (p-values = 0.01− < 0.001). As delayed response speed in the FND groups could unduly impair performance on several other *CANTAB* tests, *MST* and *RTT* variables were entered as covariates in sensitivity analyses for all other *CANTAB* outcomes.

Sustained attention test performance (*CANTAB RVIP*) differed significantly between groups on several indices – *Ability, Total Misses, Probability of Hit*, and *Median Response Latency* (Table 3). These differences endured all sensitivity analyses (Supplementary Table 12), aside from the group effect on *RVIP Median Response Latency*, which became non-significant after excluding *MSVT* fails and/or including *RTT Mean Reaction Time* as a covariate. Both FND groups displayed significantly inferior performance to HC and CCs on all other *RVIP* outcomes, assessed with Bonferroni post hoc tests (Supplementary Table 13, p-values = 0.03- < 0.001).

Set-shifting test performance (*CANTAB IEDSS)* deviated significantly between-groups on *Total Errors, Adjusted Errors, Pre-Extra-Dimensional Shift Errors, Total Trials Completed*, and *Response Latency* (Table 3). These results remained stable in sensitivity analyses (Supplementary Table 14), except *Adjusted Error* and *Pre-Extra-Dimensional Shift Errors*, which dissipated after controlling for MSVT fails, education, gender, and/or response latency (*CANTAB MST/RTT variables*). Post hoc analyses confirmed that the FS and FMS groups had significantly more *IEDSS Total Errors* and *Total Trials Completed*, and showed extended *Response Latency*, compared to CCs (Supplementary Table 15, p-values = 0.01−0.001).

None of the significant group main effects and/or interactions on *CANTAB Spatial Span, Stop Signal, Emotion Bias, or Emotion Recognition* test performance remained stable in sensitivity analyses accounting for *MSVT* fails, education, *FSIQ-2* performance, psychotropic medication, and/or *CANTAB MST/RTT* scores (Supplementary Tables 16–22).

### Metacognitive confidence ratings

Both FND groups expressed significantly lower post-diction confidence for sustained attention test (*CANTAB RVIP*) performance than HCs, and the FMS group also reported reduced confidence on this task compared to CCs (Supplementary Tables 23–25, p-values<0.001). The groups did not differ in post-diction confidence ratings for any other test.

### Exploratory correlational analyses

Within-groups (FS/FMS/CC) correlations examined potential associations between sustained attention and set-shifting test outcomes *(CANTAB RVIP/IEDSS)*, post-diction metacognitive confidence ratings, self-reported cognitive symptoms, and other clinical variables (Table 4). Only correlations that remained significant after Benjamini–Hochberg correction are reported.

**Table 4.** Significant within-group correlations

Outcome variable	FS		FMS		CC	
	*r/r_s_*	*p*	*r/r_s_*	*p*	*r/r_s_*	*p*
CFQ Total						
GAD-7	0.38	**0.008**	0.38	**0.006**	0.34	**0.02**
PHQ-9	0.45	**0.001**	0.48	**<0.001**	0.45	**0.001**
MDI Disengagement T	0.59	**<0.001**	0.63	**<0.001**	0.49	**<0.001**
MDI Depersonalisation T	0.42	**0.003**	0.34	**0.02**	0.28	0.06
MDI Derealisation T	0.42	**0.003**	0.41	**0.003**	0.21	0.16
MDI Emotional Constriction T	0.32	0.03^a^	0.29	0.04^a^	0.37	**0.01**
MDI Memory Disturbance T	0.46	**<0.001**	0.59	**<0.001**	0.36	**0.01**
MDI Identity Disturbance T	0.22	0.12	0.46	**<0.001**	0.18	0.23
SDQ-20	0.45	**0.001**	0.46	**<0.001**	0.43	**0.002**
PHQ-15	0.45	**0.001**	0.23	0.11	0.39	**0.006**
FNSQ Total	0.02	0.88	0.43	**0.002**	NA	NA
FNSQ Severity	−0.09	0.53	0.34	**0.02**	NA	NA
FNSQ Impact	−0.20	0.17	0.30	**0.03**	NA	NA
WSAS	−0.03	0.83	0.39	**0.005**	0.50	**<0.001**
SF-36 Physical Function	−0.07	0.66	−0.38	**0.007**	−0.57	**<0.001**
SF-36 Role Limitations – Physical	−0.17	0.26	−0.50	**<0.001**	−0.43	**0.003**
SF-36 Role Limitations – Emotional	−0.43	**0.002**	−0.44	**0.002**	−0.48	**<0.001**
SF-36 Energy	−0.22	0.15	−0.33	**0.03**	−0.27	0.06
SF-36 Emotional Well-being	−0.35	**0.02**	−0.43	**0.002**	−0.34	**0.02**
SF-36 Social Functioning	−0.11	0.45	−0.47	**<0.001**	−0.42	0.003
SF-36 Pain	−0.18	0.23	−0.48	**<0.001**	−0.52	**<0.001**
SF-36 General Health	−0.53	**<0.001**	−0.36	**0.01**	−0.41	**0.004**
RVIP Performance ratings						
CFQ Total	−0.09	0.55	−0.43	**0.002**	−0.22	0.14
RVIP Ability	0.27	0.07	0.48	**<0.001**	0.24	0.10
RVIP Probability of Hit	0.19	0.21	0.36	**0.01**	0.22	0.12
RVIP Total Misses	−0.19	0.21	−0.36	**0.01**	−0.22	0.12
PHQ-9	0.14	0.37	−0.39	**0.005**	−0.21	0.15
GAD-7	0.02	0.88	−0.37	**0.007**	−0.09	0.53
MDI Disengagement T	−0.10	0.51	−0.53	**<0.001**	−0.11	0.45
MDI Depersonalisation T	0.17	0.25	−0.36	**0.01**	−0.00	0.98
MDI Derealisation T	−0.02	0.89	−0.31	0.03^a^	−0.08	0.60
MDI Emotional Constriction T	0.07	0.63	−0.29	0.04^a^	−0.13	0.38
MDI Memory Disturbance T	−0.10	0.51	−0.59	**<0.001**	−0.13	0.40
MDI Identity Disturbance T	0.36	0.01^a^	−0.36	**0.01**	0.14	0.36
SF-36 Role Limitations – Physical	0.01	0.94	0.54	**<0.001**	−0.13	0.38
RVIP Ability						
IEDSS Total Errors	−0.24	0.15	−0.48	**0.002**	−0.16	0.30
IEDSS Total Trials	−0.33	0.04^a^	−0.48	**0.002**	−0.17	0.27
IEDSS Response Latency	−0.23	0.16	−0.35	**0.03**	−0.32	0.04^a^
RVIP Probability of Hit						
IEDSS Total Errors	−0.14	0.42	−0.47	**0.003**	−0.14	0.38
IEDSS Total Trials	−0.24	0.15	−0.42	**0.008**	−0.15	0.33
IEDSS Response Latency	−0.14	0.42	−0.32	**0.048**	−0.32	0.04^a^
RVIP Total Misses						
IEDSS Total Errors	0.14	0.42	0.47	**0.003**	0.14	0.38
IEDSS Total Trials	0.15	0.33	0.42	**0.008**	0.15	0.33
IEDSS Response Latency	0.14	0.42	0.32	**0.048**	0.32	0.04^a^
IEDSS Performance ratings						
IEDSS Total Errors	−0.53	**<0.001**	−0.38	**0.02**	−0.09	0.58
IEDSS Total Trials	−0.50	**<0.001**	−0.40	**0.01**	−0.10	0.52
IEDSS Response Latency	−0.37	**0.02**	−0.45	**0.004**	−0.14	0.36

*Note:* CC, ‘clinical controls’; CFQ, ‘Cognitive Failures Questionnaire’; FMS, ‘functional motor symptoms’; FNSQ, ‘Functional Neurological Symptoms Questionnaire’; FS, ‘functional seizures’; GAD-7, ‘Generalised Anxiety Disorder-7’; IEDSS=Intra-Extra Dimensional Set Shift’; MDI, ‘Multiscale Dissociation Inventory’; PHQ, ‘Patient Health Questionnaire’; RVIP, ‘Rapid Visual Information Processing’; SDQ-20, ‘Somatoform Dissociation Questionnaire-20’; SF-36-Short-Form Health Survey-36 item’; WSAS, ‘Work and Social Adjustment Scale’. Bold values indicate statistically significant (p < 0.05).

a Did not survive Benjamini–Hochberg correction (5%).

#### Sustained attention and set-shifting performance outcomes

In the FMS group, significant associations were found between performance outcomes on both the sustained attention (*CANTAB RVIP*) and set-shifting tasks (*CANTAB IEDSS*).

Performance outcomes on the sustained attention (*CANTAB RVIP*) and set-shifting (*CANTAB IEDSS*) tests did not correlate significantly with self-reported cognitive symptoms (*CFQ Total)* in any group. However, in both FMS and FS groups, poorer set-shifting task outcomes *(CANTAB IEDSS)* were significantly associated with reduced post-diction confidence on that test. Additionally, in the FMS group, worse performance on the sustained attention test outcomes *(CANTAB RVIP)* correlated with lower post-diction confidence ratings for the task.

#### Clinical variables

Self-reported cognitive symptoms (*CFQ-Total*) correlated positively with several clinical features in the FND/CC samples, including anxiety, depression, and psychological/somatoform dissociation. *CFQ-Total* scores were also related to physical symptoms in the FS/CC groups, and to greater FNS severity and impact in the FMS group. Furthermore, *CFQ-Total* scores were associated with work/social functioning in the FMS/CC groups, and quality-of-life in FMS/FS/CC groups.

In the FMS sample only, post-diction metacognitive confidence for sustained attention performance (*RVIP*) correlated with self-reported cognitive symptoms, psychological dissociation, depression, anxiety, and role limitations due to physical factors.

## Discussion

We examined self-reported cognitive difficulties, neurocognitive test performance, and metacognitive confidence in patients with FMS or FS, compared to healthy and clinical controls (HC/CC). As predicted, the FND groups reported greater self-reported cognitive symptom burden, combined with localised performance impairments on tests of sustained attention and set-shifting. In contrast to our hypotheses, there were no consistent group differences in performance on tests of response inhibition, working memory, or social–emotional processing. In the FMS and/or FS groups, impaired test performance in sustained attention and/or set-shifting was associated with reduced metacognitive confidence in those domains, suggesting broadly accurate self-appraisals in those domains.

### Self-reported cognitive symptoms

Elevated self-reported cognitive symptoms in our FS and FMS samples replicated several studies (Butler et al., 2021; Heintz et al., 2013; Pick et al., 2023; Teodoro et al., 2018; Vechetova et al., 2022), showing that subjective cognitive symptoms are a significant challenge for individuals with FND, beyond those with a primary diagnosis of FCD. As found in other populations, cognitive difficulties were significantly related to a range of clinical features in our FS/FMS samples, but they did not correlate meaningfully with test performance deficits (Forejtova et al., 2023; Goodman, Timpano, Llabre, & Bainter, 2022; Millman et al., 2025; Pick et al., 2023; Van Patten et al., 2025). Clinical correlates of self-reported cognitive symptoms included depression, anxiety, dissociation, physical symptoms, quality-of-life, and work/social functioning, with some of these associations more prominent in our FMS sample. These self-reported cognitive concerns were heightened in our FS/FMS samples compared to CCs, who were comparable in anxiety and depression severity.

Together, these findings underscore the prominence and potential impact of cognitive concerns in FND and provide preliminary evidence that these difficulties may be particularly relevant in patients with FMS. Our results also indicate that self-reported cognitive symptoms in FND may not be due solely to the presence of elevated psychological distress (i.e., anxiety/depression). Future research should evaluate the extent to which self-reported cognitive symptoms in FND are related to observable cognitive deficits in real-world settings, in addition to ascertaining the direction of influence between self-reported cognitive symptoms and other clinical outcomes. Importantly, patients with FMS and FS may require targeted interventions to facilitate management of cognitive concerns and their impact in everyday life (Poole et al., 2025).

### Neurocognitive test performance

Test performance indicated reduced intellectual functioning and extended motor/cognitive response speed in one or both FND groups in this study, corresponding with some previous reports (Millman et al., 2025; Van Patten et al., 2024a), although intellectual functioning was in the normal range in all groups in this study. Nonetheless, some of our primary results failed to survive sensitivity analyses, including one or more of these variables, reinforcing the importance of assessing their influence in future neuropsychological studies in FND.

The lack of robust group differences in performance on tests of response inhibition, working memory, and social–emotional processing tests countered our predictions, adding to the inconsistent findings in these domains (Millman et al., 2025; Pick et al., 2019; Van Patten et al., 2024a). More thorough assessment of constituent processes with multiple tests may be necessary to uncover disorder-specific deficits in these cognitive functions.

Performance deficits on sustained attention (*CANTAB RVIP*) and set-shifting (*CANTAB IEDSS*) tasks exhibited by our FS and FMS samples withstood sensitivity analyses, therefore representing compelling evidence for demonstrable, specific executive function difficulties in these groups. The findings relating to sustained attention performance align with prior accounts of attentional control impairments in FND samples (Alluri et al., 2020; Millman et al., 2025; Van Patten et al., 2024a; Vechetova et al., 2022). Our observation of weaker performance on the set-shifting task (*CANTAB IEDSS*) in the FND groups differed from two previous studies that used the same test (O’Brien et al., 2015; Pick et al., 2023), although the pattern of deficits seen here suggested overall rule-learning difficulties during the task, rather than set-shifting performance specifically. Effect sizes were large for sustained attention test performance in this sample, whereas they were small to medium for set-shifting performance, demonstrating that differences in the former domain might be more clinically meaningful.

Impaired attentional control might represent a valuable neurocognitive marker in FS and FMS, characterised by diminished maintenance of attentional focus, together with difficulties in learning implicit rules and/or voluntarily switching from a particular cognitive set. However, given that test performance in these domains did not correlate meaningfully with clinical outcomes (e.g., FND, cognitive, psychological, and physical symptoms, quality-of-life, work/social functioning), the relevance of these differences to FND should be scrutinised in greater detail. Investigators may also examine executive function performance in FS/FMS relative to patients with FCD, to identify possible shared and distinctive profiles and mechanisms in these subgroups.

### Performance validity

A minority of participants in this study failed the performance validity test (*MSVT*), although the proportion was not significantly different between the FS/FMS/CC/HC groups (mean failure rates = 2–8%), consistent with a number of other studies providing no clear evidence of weaker performance validity in FND than in other clinical groups (McWhirter et al., 2020). The rates of *MSVT* failure in the present study were considerably lower than pooled performance validity test failure base-rates observed across clinical populations (16%) and in FS specifically (33%), although these values were based on patients being evaluated in clinical settings (Roor et al., 2024). As previously noted, various methodological issues may introduce bias in performance validity testing studies, such as inclusion of individuals with external motivation (e.g., ongoing litigation), medication-related impairments, and/or with psychiatric, cognitive and/or physical symptoms that might alter motivation or attentional focus on the tasks (McWhirter et al., 2020; Roor et al., 2024).

With the inclusion of both HC and CC groups in this statistically powered study, we were able to demonstrate that performance validity was not unduly disrupted in our FS and FMS samples, while accounting for the presence of psychotropic medications and general psychiatric symptoms (i.e., depression, anxiety). Our findings strengthen the literature showing intact effort and engagement in neuropsychological testing in FND (McWhirter et al., 2020). Nonetheless, it is notable that most previous studies focused on performance validity in FS samples only, with less data available for FMS or other FND subgroups. Inclusion of performance validity measures in future studies in all FND phenotypes would be valuable to ensure that this potential confound is consistently accounted for.

### Metacognitive confidence

Participants with FS and FMS expressed markedly reduced post-diction metacognitive confidence on the sustained attention task, mirroring the performance deficit displayed on this measure. Actual performance and post-diction confidence ratings on the set-shifting task (*CANTAB IEDSS*) were correlated within both FND groups, but not in HC/CC. Furthermore, sustained attention performance (*CANTAB RVIP*) correlated with post-diction confidence in the FMS group only, not in FS/CC/HC groups. These results indicate that the FND groups were generally accurate in appraising their performance on these measures, and possibly more so than the CC/HC comparison groups, suggesting intact *metacognitive sensitivity* in the domain of cognitive control (i.e., the statistical relationship between test performance and confidence ratings). This contrasts with our hypothesis and some theoretical perspectives, although it is consistent with several previous studies that have not supported the presence of local metacognitive deficits in FND samples (Sadnicka et al., 2025). As such, the emerging picture is that second-order metacognition may be unimpaired in FND, countering the proposition that altered self-evaluation of cognitive processing is an underlying mechanism in the disorder (Sadnicka et al., 2025).

In this study, post-diction metacognitive confidence ratings for sustained attention test performance, but not actual performance on that test, correlated significantly with self-reported cognitive symptoms in the FMS group, replicating our pilot findings (Pick et al., 2023). Lower post-diction confidence for sustained attention performance was also associated with other adverse clinical characteristics in the FMS group. Therefore, reduced local metacognitive confidence in attentional control might contribute to generally increased subjective cognitive concerns and/or other clinical outcomes in FMS, beyond the degree of actual performance deficits displayed. If replicated, these results imply that short-term (local) attentional performance self-appraisals may be important treatment targets in cognitive rehabilitation programs for patients with FND experiencing disruptive cognitive symptoms.

Our findings highlight some clear directions for future research on metacognition in FND. More rigorous experimental studies are needed to examine local metacognition on a trial-by-trial basis, to examine *metacognitive efficiency* (i.e., performance-controlled assessment of metacognitive sensitivity), and to utilise a comprehensive range of established local metacognitive metrics (Fleming & Lau, 2014), with careful consideration of potential confounds (Sadnicka et al., 2025). These experimental measures could be combined with post-diction test-level confidence ratings and measures of global metacognition, as used in the present study, to provide multidimensional assessments in specific FND subgroups (e.g., FS, FMS, FCD, functional sensory symptoms). Further investigations should also clarify the direction of effects between local metacognition for attentional control and relevant clinical outcomes (e.g., self-reported cognitive concerns, depression, anxiety). Finally, an examination of the potential benefits of targeted metacognitive training on cognitive symptoms and other clinical outcomes seems warranted, with a particular focus on improving local metacognitive confidence in cognitive control.

### Strengths and limitations

The major strength of this study was the direct comparison of two common FND subgroups using the same test battery, with a sample size providing adequate statistical power to detect small effects. Inclusion of HC and CC groups provided insights into cognitive features in FND that were distinct from the general population, while controlling for elevated psychological distress. We employed a range of measures to comprehensively assess cognitive performance, along with two measures of metacognitive functioning (self-reported cognitive symptoms, test-specific post-diction confidence), in addition to a test of performance validity. Our groups were comparable on most relevant background characteristics (e.g., age, gender, handedness), and we accounted for any pertinent group differences within our analyses (e.g., education, response speed, psychotropic medications). Examining potential relationships between cognitive variables and other clinical features allowed us to explore the clinical significance of neurocognitive differences in our FS/FMS/CC samples.

Some limitations of this study should be considered. We did not include a group with FCD because this was beyond the remit of the broader research program within which this study took place. While we controlled statistically for the influence of general intellectual functioning, education, psychotropic medications, and other confounds, there may have been residual effects on our results. A minority of our FND/CC samples reported diagnoses of attention-deficit hyperactivity disorder, which may have affected attentional performance. The use of a retrospective questionnaire to assess self-reported cognitive symptoms may have resulted in recall biases. Finally, the *CANTAB Connect* test battery currently lacks robust psychometric evaluation and has limited ecological validity.

## Conclusions

Our findings provide robust evidence of performance deficits on tests of sustained attention and set-shifting in FS and FMS, which were associated with reduced post-diction metacognitive confidence in these domains, and which were accompanied by elevated self-reported cognitive symptoms in daily life. Poorer subjective cognitive functioning was associated with greater physical and psychological symptom burden, decreased work and social functioning, and worse quality-of-life, in patients with FND and/or common psychological symptoms (anxiety/depression). Neuropsychological rehabilitation approaches might hold promise for some patients with FMS and FS, specifically targeting cognitive control and associated self-appraisals in everyday activities, and facilitating self-management of subjective cognitive symptoms and their impact.

## Supporting information

10.1017/S0033291726103420.sm001 Pick et al. supplementary material Pick et al. supplementary material

## Data Availability

The anonymised dataset will be made available in response to reasonable requests, on a case-by-case basis. Completion of a Data Sharing Agreement will be required, facilitated by the King’s College London Data Management team.

## Supplementary material

The supplementary material for this article can be found at http://doi.org/10.1017/S0033291726103420.
