# Supplementary material for: Cognition and metacognition in functional motor symptoms and functional seizures: a case–control study
Source: Psychol Med. 2026 Mar 3;56:e61. doi: 10.1017/S0033291726103420 (PMC12969200; doi:10.1017/S0033291726103420)
Supplement: Pick et al. supplementary material [file S0033291726103420sup001.docx]

**Cognition and metacognition in FND: A case-control study**

**Supplementary Materials**

**Contents**

Supplementary Table 1. Clinical self-report questionnaire details – page 2

Supplementary Table 2. Functional Neurological Symptoms Questionnaire – page 3

Supplementary Table 3. CANTAB Connect outcome variables – page 4

Supplementary Table 4. Sample characteristics: Sociodemographic and clinical variables – page 7

Supplementary Table 5. Clinical self-report measure statistics – page 9

Supplementary Table 6. Cognitive Failures Questionnaire sensitivity analyses – page 10

Supplementary Table 7. Cognitive Failures Questionnaire Bonferroni post-hoc tests – page 11

Supplementary Table 8. WASI-II post-hoc tests – page 11

Supplementary Table 9. WASI-II sensitivity analyses – page 12

Supplementary Table 10. Motor Screening and Reaction Time Test sensitivity analyses – page 14

Supplementary Table 11. Motor screening and reaction time Games-Howell post-hoc tests – page 16

Supplementary Table 12. Rapid Visual Information Processing sensitivity analyses – page 17

Supplementary Table 13. Rapid Visual Information Processing Bonferroni post-hoc tests – page 20

Supplementary Table 14. Intra-Extra Dimensional Set-Shift sensitivity analyses – page 21

Supplementary Table 15. Intra-Extra Dimensional Set Shift post-hoc tests – page 25

Supplementary Table 16. Spatial Span Test uncorrected results – page 26

Supplementary Table 17. Spatial Span Test sensitivity analyses – page 26

Supplementary Table 18. Stop Signal Task uncorrected results – page 29

Supplementary Table 19. Stop Signal Task sensitivity analyses – page 29

Supplementary Table 20. Emotional bias and recognition tasks uncorrected results – page 31

Supplementary Table 21. Emotional Bias Task sensitivity analyses – page 32

Supplementary Table 22. Emotion Recognition Test sensitivity analyses – page 38

Supplementary Table 23. Metacognitive performance ratings – page 45

Supplementary Table 24. RVIP performance ratings sensitivity analyses – page 46

Supplementary Table 25. RVIP performance ratings post-hoc tests – page 47

**Supplementary Table 1. Clinical self-report questionnaire details**

| **Questionnaire** | **Description** |
| --- | --- |
|  |  |
| ***Functional Neurological Symptoms Questionnaire (Pick et al., 2024)*** | A tailored questionnaire measuring the presence, severity and impact of varied functional neurological symptoms over 1-week. Scores can be calculated for: total number of functional neurological symptoms, average severity (1-7) and average impact (1-7). Worse severity, impact and number of functional neurological symptoms are indicated by higher scores. |
| ***Patient Health Questionnaire – 15 (Kroenke et al., 2002)*** | 15 items assess the frequency of common somatic symptoms over the previous four weeks. Scores range from 0-30 – higher scores indicate more somatic symptoms. |
| ***Patient Health Questionnaire – 9 (Kroenke et al., 2001)*** | 9 items capturing the frequency of depressive symptoms over 2-weeks. Scores range from 0-27 –elevated depressive symptoms are indicated by higher scores. |
| ***Generalised Anxiety Disorder – 7 (Spitzer et al., 1999)*** | 7 items assess the frequency of generalised anxiety symptoms in the over 2-weeks. Scores range from 0-21 – greater anxiety is denoted by higher scores. |
| ***Somatoform Dissociation Questionnaire – 20 (Nijenhuis et al., 1996)*** | 20 items examining the extent of various somatoform symptoms in the last year (e.g., sensory disturbances, speech/swallowing difficulties, pain). Scores range from 20-100 – higher scores indicate greater somatoform dissociation. |
| ***Multiscale Dissociation Inventory (Briere, 2002)*** | 30 items examining the frequency of psychological dissociation over a 1-month period. T-scores of 0-170 for six subscales: Disengagement, Depersonalisation, Derealisation, Emotion Constriction, Memory Disturbance, Identity Dissociation. Higher scores indicate greater dissociative symptomology. |
| ***Short Form Health Survey – 36 (Hays et al., 1993)*** | 36 items assessing eight domains of health-related quality-of-life, including physical functioning, emotional wellbeing, role limitations due to physical health, role limitations due to emotional difficulties, social functioning, energy/fatigue, pain, and general health perceptions. Subscale scores range from 0-100 - better quality-of-life is indicated by higher scores. |
| ***Work & Social Adjustment Scale (Mundt et al., 2002)*** | 5 items summarising general functioning in social and occupational domains. Scores range from 0-40 – worse functioning is indicated by higher scores. |

Adapted from Pick et al. (2023). Objective and subjective neurocognitive functioning in functional motor symptoms and functional seizures: preliminary findings. J Clin Exp Neuropsychol 2023; 45(10): 970-87 (license CC BY 4).

**References**

- Briere, J., Weathers, F. W., Runtz, M. (2005). Is dissociation a multidimensional construct? Data from the Multiscale Dissociation Inventory. J Trauma Stress, 18(3):221-31. doi: 10.1002/jts.20024.
- Guy, W. B. R. R. (1976). CGI. Clinical global impressions. ECDEU assessment manual for psychopharmacology.
- Hays, R. D., et al. (1993). The RAND 36-Item Health Survey 1.0. Health Econ, 2(3):217-27. doi: 10.1002/hec.4730020305. PMID: 8275167.
- Kroenke, K., Spitzer, R. L., Williams, J. B. (2001). The PHQ-9: validity of a brief depression severity measure. J Gen Intern Med, 16(9):606-13. doi: 10.1046/j.1525-1497.2001.016009606.x.
- Kroenke, K., Spitzer, R. L., Williams, J. B. (2002). The PHQ-15: validity of a new measure for evaluating the severity of somatic symptoms. Psychosom Med, 64(2):258-66. doi: 10.1097/00006842-200203000-00008.
- Mundt, J. C., et al. (2002). The Work and Social Adjustment Scale: a simple measure of impairment in functioning. Br J Psychiatry, 180:461-4. doi: 10.1192/bjp.180.5.461.
- Nijenhuis, E. R., et al. (1996). The development and psychometric characteristics of the Somatoform Dissociation Questionnaire (SDQ-20). J Nerv Ment Dis, 184(11):688-94. doi: 10.1097/00005053-199611000-00006.
- Pick, S., et al. (2024). Investigating psychobiological causes and mechanisms in functional seizures and functional motor symptoms: Study protocol. PloS One, 19(6), e0305015. <https://doi.org/10.1371/journal.pone.0305015>
- Spitzer, R. L., Kroenke, K., Williams, J. B., Löwe, B. (2006). A brief measure for assessing generalized anxiety disorder: the GAD-7. Arch Intern Med,166(10):1092-7. doi: 10.1001/archinte.166.10.1092.

**Supplementary Table 2. Functional Neurological Symptoms Questionnaire (Pick et al., 2024)**

Please look at the symptoms in the table below and tell us whether you have experienced these functional neurological symptoms in the **past week.** If you mark yes to indicate that the symptom was present in the past week, please complete the additional columns to tell us **how frequent** the symptoms were, **how severe** (intense) they were, and **how much impact** they had on you.

When rating the average **severity** of symptoms, please choose a number from 1 to 7, where **1=Symptom not present; 2=Minimal; 3=Mild; 4=Moderate; 5=Moderately severe; 6=Severe; 7=Very severe**.

When rating the **impact** of symptoms, please choose a number from 1 to 7, where **1=No impact at all; 2=Minimal impact; 3=Mild impact; 4=Moderate impact; 5=Moderately severe impact; 6=Severe impact; 7=Very severe impact**.

| **FND Symptom** | **Present?**  (circle or bold) | **Frequency**  (circle or bold) | **Average severity**  **(1-7)** | **Average impact**  **(1-7)** |
| --- | --- | --- | --- | --- |
| Weakness | Yes / No | Constant / daily / weekly / less than weekly |  |  |
| Tremor | Yes / No | Constant / daily / weekly / less than weekly |  |  |
| Dystonia (muscle spasms / fixed postures) | Yes / No | Constant (1) / daily (2) / weekly (3) / less than weekly (4) |  |  |
| Walking / mobility difficulties | Yes / No | Constant / daily / weekly / less than weekly |  |  |
| Myoclonus (muscle jerks) | Yes / No | Constant / daily / weekly / less than weekly |  |  |
| Seizures* | Yes / No | Number of seizures in the last week: |  |  |
| Numbness (loss of feeling) | Yes / No | Constant / daily / weekly / less than weekly |  |  |
| Visual disturbances | Yes / No | Constant / daily / weekly / less than weekly |  |  |
| Sensitivity to light/sound | Yes / No | Constant / daily / weekly / less than weekly |  |  |
| Dizziness | Yes / No | Constant / daily / weekly / less than weekly |  |  |
| Speech / swallowing difficulties | Yes / No | Constant / daily / weekly / less than weekly |  |  |
| Cognitive difficulties (e.g., brain fog, memory lapses) | Yes / No | Constant / daily / weekly / less than weekly |  |  |
| Other FND symptoms | Details: | Constant / daily / weekly / less than weekly |  |  |

Please tell us which FND symptom(s) is most severe and has the most impact on you:

*If you experience FND seizures, do you have warning symptoms? Yes / No

*If you experience warning symptoms before an FND seizure, what is the earliest or most consistent symptom(s) that you experience?

As presented in Pick et al. (2024). Investigating psychobiological causes and mechanisms in functional seizures and functional motor symptoms: Study protocol. PLoS One 2024; 19(6): e0305015. (license CC BY 4.0).

**Supplementary Table 3. CANTAB Connect outcome variables**

| **Task** | **Outcome variables** | **Description** | **Interpretation** |
| --- | --- | --- | --- |
| **Motor Screening Test** | Motor Mean Latency | Mean latencies for participants’ correct responses to stimuli presented during assessed trials (milliseconds). | Higher scores indicate poorer performance. |
| **Reaction Time** | Reaction Time | Median latencies for release of the response button after stimulus presentation. | Higher scores indicate poorer performance. |
|  | Movement Time | Median duration for release of the response button and selection of the target stimulus. | Higher scores indicate poorer performance. |
|  | Total Error Score | Total number of assessed trials eliciting any form of erroneous response: inaccurate responses, incorrect location, omission, premature errors, manual errors (e.g., use of multiple fingers). | Higher scores indicate poorer performance. |
| **Rapid Visual Information Processing** | Response Latency | Median duration for correct responses (milliseconds). | Higher scores indicate poorer performance. |
|  | RVIP Ability | Signal detection index of overall ability in identifying target sequences. | Range = 0.00 to 1.00 (poor to good performance) |
|  | Probability of False Alarm | Total number of false alarms (incorrectly selected number sequences), divided by the number of false alarms plus the number of correct rejections. | Higher scores indicate poorer performance. |
|  | Probability of Hit | Total number of correct sequence detections (Hit), divided by the number of target sequences presented. | Higher scores indicate superior performance. |
| **Spatial Span** | Forward Span Length | Number of items in a sequence that were successfully recalled. | Higher scores indicate stronger performance. |
|  | Reverse Span Length |  |  |
| **Intra-Extra Dimensional Set Shift** | Total Errors | Total number of assessed trials eliciting an incorrect response within the response window. | Higher scores indicate worse poorer performance. |
|  | Adjusted Errors | Total number of incorrect responses, adjusted for incomplete stages.  Total Errors Adjusted = Total Errors (stages reached) + [(number of unreached stages) * (25)]. | Higher scores indicate poorer performance. |
|  | Extra-Dimensional Shift Errors | Total number of trials on which a participant failed to select the correct stimulus on the stage where the extra-dimensional shift occurs. | Lower scores indicate better performance. |
|  | Pre-Extra Dimensional Shift Errors | The number of errors in stimulus selection on all trials prior to the extra-dimensional shift. Errors are trials on which the participant fails to select the stimulus that is compatible with the current rule. | Lower scores indicate superior performance. |
|  | Trials completed | Number of trials completed on all stages. | Higher scores indicate poorer performance. |
|  | Adjusted trials completed | Number of trials completed on all attempted stages, adjusted for stages not attempted.    Total Trials Adjusted = Total Trials (stages reached) + [(number of unreached stages) * (50)] | Higher scores indicate worse performance. |
|  | Completed Stage Errors | Total number of incorrect responses on completed stages. | Higher scores indicate poorer performance. |
|  | Stages Completed | Total number of stages completed (0-9) | Higher scores indicate stronger performance. |
|  | Response Latency | Sum of response times across all trials (milliseconds). | Higher scores indicate weaker performance. |
| **Stop Signal Task** | Stop Signal Reaction Time | An estimate of time in which a participant can successfully inhibit their responses 50% of the time. Calculated from the duration between the ‘Go’ and ‘Stop’ stimuli at which the participant can successfully inhibit their response on 50% of trials. | Higher scores indicate poorer performance. |
|  | Errors: Go Trials | Total number of trials where the participant made an erroneous response to the direction of the arrow stimulus on a Go trial. | Higher scores indicate poorer performance. |
|  | Errors: Stop Trials | Total number of trials where the participant made an erroneous response to the direction of the arrow stimulus on a Stop trial. | Higher scores indicate poorer performance. |
|  | Missed Trials | Total number of trials which the participant missed. | Higher scores indicate diminished performance. |
| **Emotional Bias Task** | Bias Point | Proportion of assessed trials on which the participants selected 'Happy', adjusted to a scale of 0 to 15.  (Number of assessed trials selected as 'Happy'/Number of all assessed trials) x 15 | Higher scores indicate a greater bias towards choosing 'Happy'. |
|  | Reaction Time | Mean / median total reaction time.  Reaction time for happy selections. | Higher scores indicate poorer performance. |
| **Emotional Recognition Task** | Total reaction time | Median time taken for a participant to select an emotion after being presented with a stimulus, across all trials. | Higher scores indicate poorer performance. |
|  | Reaction time by emotion | Median duration for a participant to select an emotion after being presented with a stimulus for each emotion category. | Higher scores indicate poorer performance. |
|  | Total Hits | Total number of correct emotion selections across all trials. | Higher scores indicate better performance. |
|  | Hits by emotion | Total number of correct emotion selections made for each emotion category. | Higher scores indicate superior performance. |
|  | Unbiased Hit Rate | A measure that reflects emotion recognition accuracy after accounting for guesses and other response biases. | Higher scores indicate stronger performance. |
|  | False alarms | Number of erroneous emotion selections across all trials. | Higher scores indicate worse performance. |

Adapted from Pick et al. (2023). Objective and subjective neurocognitive functioning in functional motor symptoms and functional seizures: preliminary findings. J Clin Exp Neuropsychol 2023; 45(10): 970-87 (license CC BY 4).

**Supplementary Table 4. Sample characteristics: Sociodemographic and clinical variables**

| **Clinical variable** | **FS**  **(n=50)** | **FMS**  **(n=50)** | **CC**  **(n=50)** | **HC**  **(n=50)** | **Test statistics** |
| --- | --- | --- | --- | --- | --- |
| ***Dependents: n (%)*** | Y= 12 (24)  N=38 (76) | Y= 11(22)  N=39 (78) | Y= 9 (18)  N=41(82) | Y= 6 (12)  N= 44 (88) | χ²(3, n=200) = 2.73, p=0.46, v=0.12 |
| ***Relationship status - single: n (%)*** | 31 (62) | 26 (52) | 29 (58) | 28 (56) | Fisher’s exact=4.58, p=0.93, v=0.09 |
| ***Carer: n (%)*** | Y= 6 (12)  N=44 (88) | Y= 3 (6)  N=47 (94) | Y= 5 (10)  N=45 (90) | Y= 4 (8)  N=46 (92) | Fisher’s exact=1.26, p=0.84, v=0.08 |
| ***Ethnicity: n (%)*** | White=36 (72)  Black=3 (6) Asian=2 (4) Other=9 (18) | White=45 (90)  Black=1 (2) Asian=2 (4) Other=2 (4) | White=30 (60)  Black=2 (4) Asian=15 (30)  Other=3 (6) | White=35 (70)  Black=1 (2) Asian=11 (22)  Other=3 (6) | Fisher’s exact=27.71, **p<0.001**, v=0.22 |
| ***Occupational status – employed / student: n (%)*** | 23 (46) | 25 (50) | 44 (88) | 42 (84) | χ² (3, n=200)=33.02, **p<0.001**, v=0.41 |
| ***CGI severity: M (SD)*** | 4.98 (8.9) | 4.72 (0.97) | NA | 3.6 (1.07) | *F (2,97.5)=26.05, **p<0.001**, ω²=0.27 |
| ***Illness duration - years: M (SD)*** | 8 (9.52) | 4.77 (5.33) | NA | 15.32 (11.78) | *F (2,87.4)=16.95, **p<0.001**, ω²=0.18 |
| ***Mental health diagnoses***  ***(Quick-SCID)*** | **n (%)** | **n (%)** | **n (%)** | **n (%)** |  |
| *Generalised anxiety disorder (current)* | 11 (22) | 9 (18) | 22 (44) | 0 (0) |  |
| *Major depressive disorder* | 33 (66) | 34 (68) | 48 (96) | 0 (0) |  |
| *Agoraphobia (current)* | 6 (12) | 4 (8) | 4 (8) | 0 (0) |  |
| *Social Anxiety Disorder (current)* | 4 (8) | 4 (8) | 8 (16) | 0 (0) |  |
| *Panic Disorder (current)* | 5 (10) | 3 (6) | 3 (6) | 0 (0) |  |
| ***Other mental health diagnoses (self-report, lifetime)*** |  |  |  |  |  |
| *Post-Traumatic Stress Disorder* | 10 (20) | 7 (14) | 2 (4) | 0 (0) |  |
| *Neurodevelopmental disorders* | 10 (20) | 2 (4) | 4 (8) | 0 (0) |  |
| *Emotionally Unstable Personality Disorder* | 4 (8) | 4 (8) | 2 (4) | 0 (0) |  |
| *Panic Disorder* | 2 (4) | 2 (4) | 3 (6) | 0 (0) |  |
| ***Physical health diagnoses (self-report, current)*** |  |  |  |  |  |
| *Migraine* | 3 (6) | 6 (12) | 2 (4) | 0 (0) |  |
| *Fibromyalgia* | 6 (12) | 8 (16) | 1 (2) | 0 (0) |  |
| *Asthma* | 10 (20) | 6 (12) | 8 (16) | 3 (6) |  |
| *Allergies* | 2 (4) | 3 (6) | 2 (4) | 0 (0) |  |
| *Irritable bowel syndrome* | 5 (10) | 1 (2) | 1 (2) | 0 (0) |  |
| *Hypermobility* | 3 (6) | 12 (24) | 3 (6) | 0 (0) |  |
| *Hypothyroidism* | 3 (6) | 4 (8) | 2 (4) | 2 (4) |  |
| *High blood pressure* | 2 (4) | 3 (6) | 4 (8) | 0 (0) |  |
| *Polycystic ovary syndrome* | 1 (2) | 3 (6) | 2 (4) | 1 (2) |  |

**Supplementary Table 5. Clinical self-report measure statistics**

| **Outcome variable** | **FS**  **(n=50)** | **FMS**  **(n=50)** | **CC**  **(n=50)** | **HC**  **(n=50)** | **Test statistics** |
| --- | --- | --- | --- | --- | --- |
| ***Functional Neurological Symptoms Questionnaire – Total: M (SD)*** | 7.40 (3.25) | 7.62 (2.59) | - | - | t(98)=-0.37, p=0.71, d=0.08 |
| *Functional Neurological Symptoms Questionnaire – Average severity: Mdn (IQR)* | 4.14 (1.16) | 4.25 (0.97) |  |  | U=1163.50, z=-0.60, p=0.55, r=0.06 |
| *Functional Neurological Symptoms Questionnaire – Average impact: Mdn (IQR)* | 4.05 (0.99) | 4.23 (1.11) | - | - | U=1130, z=-0.83, p=0.41, r=0.08 |
| ***Somatoform Dissociation Questionnaire -20: M (SD)*** *(FS=49; CC=48)^&^* | 35.04 (11.11) | 31.06 (7.10) | 23.04 (5.05) | 20.62 (1.18) | *F(3,84.40)=62.66, **p<0.001**, ω²=0.40 |
| ***Multiscale Dissociation Inventory (MDI)*** |  |  |  |  |  |
| *MDI Disengagement T scores: M (SD) (CC=48)^&^* | 84.58 (18.00) | 76.72 (18.88) | 65.02 (12.78) | 53.92 (9.50) | *F(3,103.90)=47.55, **p<0.001**, ω²=0.36 |
| *MDI Depersonalisation T scores: M (SD) (CC=48)^&^* | 89.46 (39.61) | 82.28 (37.44) | 63.42 (24.82) | 48.06 (5.21) | *F(3,84.47)=35.36, **p<0.001**, ω²=0.22 |
| *MDI Derealisation T scores: M (SD)*  *(FS=49; CC=48)^&^* | 81.25 (27.89) | 65.58 (21.14) | 57.77 (20.81) | 47.06 (2.54) | *F(3,81.88)=39.71, **p<0.001**, ω²=0.27 |
| *MDI Emotion Constriction T scores: M (SD)*  *(CC=48)^&^* | 71.64 (26.69) | 62.74 (22.45) | 58.42 (18.41) | 48.82 (6.51) | *F(3,91.53)=18.46, **p<0.001**, ω²=0.13 |
| *MDI Memory Disturbance T scores: M (SD)*  *(CC=48)^&^* | 93.34 (36.16) | 75.32 (29.43) | 60.02 (21.53) | 50.92 (11.13) | *F(3,97.22)=28.15, **p<0.001**, ω²=0.27 |
| *MDI Identity Dissociation T scores: M (SD)*  *(CC=48)^&^* | 66.46 (39.67) | 59.28 (31.54) | 52.04 (13.36) | 47.00 (0.00) | *^$^F(2,80.74)=3.63, **p=0.03**, ω²=0.02 |
| ***Patient Health Questionnaire–9: M (SD)***  *(FS=49; CC=48)^&^* | 12.31 (6.78) | 11.02 (6.45) | 10.88 (6.68) | 2.48 (2.38) | *F(3,94.04)=63.64, **p<0.001**, ω²=0.30 |
| ***Patient Health Questionnaire–15: M (SD)***  *(CC=48)^&^* | 14.92 (6.11) | 14.18 (4.53) | 10.23 (4.77) | 4.82 (3.22) | *F(3,104.85)=65.50, **p<0.001**, ω²=0.41 |
| ***Generalised Anxiety Disorder–7: M (SD)***  *(FS=47; CC=48)^&^* | 9.87 (6.41) | 8.62 (6.03) | 8.90 (5.09) | 2.54 (3.03) | *F(3,99.80)=34.31, **p<0.001**, ω²=0.22 |
| ***Short-Form Health Survey-36 (SF-36)*** |  |  |  |  |  |
| *SF-36 Physical Functioning: M (SD) (FS=49; CC=48)^&^* | 55.00 (29.08) | 33.30 (27.06) | 85.83 (22.63) | 95.90 (6.12) | *F(3,87.47)=110.64, **p<0.001**, ω²=0.54 |
| *SF-36 Role Limitations – Physical: M (SD) (FS=49; CC=48)^&^* | 16.33 (30.0) | 14.00 (25.83) | 71.88 (39.82) | 90.50 (24.16) | *F(3,105.38)=103.07, **p<0.001**, ω²=0.55 |
| *SF-36 Role Limitations – Emotional: M (SD)*  *(FMS=48; CC=48)^&^* | 38.00 (44.68) | 50.69 (45.61) | 45.14 (40.38) | 86.67 (29.36) | *F(3,104.31)=20.08, **p<0.001**, ω²=0.17 |
| *SF-36 Emotional Wellbeing: M (SD) (CC=48)^&^* | 52.88 (19.08) | 58.24 (22.90) | 51.75 (20.67) | 78.72 (11.55) | *F(3,102.96)=36.94, **p<0.001**, ω²=0.24 |
| *SF-36 Pain: M (SD)*  *(CC=48)^&^* | 41.50 (23.55) | 36.55 (22.59) | 64.06 (25.15) | 82.50 (17.65) | *F(3,106.43)=55.06, **p<0.001**, ω²=0.40 |
| *SF-36 Social Functioning: M (SD)*  *(FS=49; FMS=49; CC=48)^&^* | 39.29 (25.77) | 39.03 (26.35) | 62.50 (29.29) | 90.80 (14.46) | *F(3,100.91)=80.24, **p<0.001**, ω²=0.43 |
| *SF-36 Energy: M (SD)*  *(FS=46; FMS=45; HC=49; CC=48)^&^* | 29.57 (19.11) | 25.23 (15.70) | 34.38 (19.91) | 60.71 (14.54) | F(3,184)=39.95, **p<0.001**, η²=0.41 |
| *SF-36 General Health: M (SD)*  *(CC=48)^&^* | 36.90 (20.63) | 33.30 (22.17) | 51.88 (21.99) | 75.10 (16.74) | F(3,194)=42.98, **p<0.001**, η²=0.40 |
| ***Work and Social Adjustment Scale: M (SD)*** *(CC=48)^&^* | 25.64 (9.42) | 26.60 (7.97) | 17.50 (10.49) | 2.62 (4.44) | *F(3,100.47)=166.89, **p<0.001**, ω²=0.57 |

**Key:** CC= clinical control; FMS= functional motor symptoms; FS=functional seizures; HC=healthy control; M=mean; SD=standard deviation

*Welch’s ANOVA ^$^ANOVA included only FS/FMS/CC groups due to floor effects in HC group ^&^Sample size deviated due to missing data

**Supplementary Table 6. Cognitive Failures Questionnaire sensitivity analyses**

| **Adjustment / Outcome variable** | **FS**  **(n=49)** | **FMS (n=50)** | **CC (n=48)** | **HC (n=50)** | **Test statistics** |  |
| --- | --- | --- | --- | --- | --- | --- |
| ***Outliers winsorised*** | | **M (SD)** | **M (SD)** | **M (SD)** | **M (SD)** | **One-way ANOVA** |
| *CFQ-Total* | | 52.81 (11.37) | 53.04 (12.54) | 45.39 (11.26) | 35.90 (9.73) | Group: *F(3,101.85)=28.74, **p<0.001**, ω²=0.28 |
| ***Education*** | **EMM (SE)** | **EMM (SE)** | **EMM (SE)** | **EMM (SE)** | **ANCOVA** |  |
| *CFQ Total* | 57.05  (2.26) | 56.86  (2.27) | 31.82  (2.26) | 45.77  (2.29) | Group: F(3,192)=26.78, **p<0.001**, η²=0.30 |  |
|  |  |  |  |  | Education: F(1,192)=1.03, p=0.31, ηp²=0.001 |  |
| ***Age*** | **EMM (SE)** | **EMM (SE)** | **EMM (SE)** | **EMM (SE)** | **ANCOVA** |  |
| *CFQ Total* | 57.25 (2.26) | 57.28 (2.24) | 45.53 (2.28) | 31.43 (2.24) | Group: F(3,192)=29.93, **p<0.001**, η²=0.32 |  |
|  |  |  |  |  | Age: F(1,192)=0.04, p=0.84, ηp²=0.00 |  |
| ***Gender*** | **EMM (SE)** | **EMM (SE)** | **EMM (SE)** | **EMM (SE)** | **ANCOVA** |  |
| *CFQ Total* | 57.19 (2.26) | 57.34 (2.24) | 45.44 (2.28) | 31.52 (2.24) | Group: F(3,192)=29.92, **p<0.001**, η²=0.32 |  |
|  |  |  |  |  | Gender: F(1,192)=0.64, p=0.43, ηp²=0.003 |  |
| ***Psychotropic medication (FS/FMS/CC groups)*** | **EMM (SE)** | **EMM (SE)** | **EMM (SE)** |  | **ANCOVA** |  |
| *CFQ Total* | 56.72  (2.23) | 56.62  (2.21) | 46.74  (2.27) | - | Group: F(2,143)=6.34, **p=0.002**, ηp²=0.08 |  |
|  |  |  |  |  | Psychotropics: F(1,143)=10.65, **p=0.001**, ηp²=0.07 |  |

**Key:** CC=clinical control; EMM=estimated marginal means; FMS=functional motor symptoms;

FS=functional seizures; HC=healthy control; M=mean; SD=standard deviation; SE=standard error

*Welch’s ANOVA

**Supplementary Table 7. Cognitive Failures Questionnaire Bonferroni post-hoc tests**

| **Outcome variable** | **Comparison** | **Mean difference (standard error)** | **Confidence interval (95%)** | **p-value** |
| --- | --- | --- | --- | --- |
| ***CFQ-Total*** | FS vs FMS | -0.02 (3.17) | -8.47, 8.44 | 1.00 |
|  | FS vs HC | 25.81 (3.17) | 17.35, 34.26 | **<0.001** |
|  | FS vc CC | 11.70 (3.20) | 3.16, 20.24 | **0.002** |
|  | FMS vs HC | 25.82 (3.16) | 17.41, 34.23 | **<0.001** |
|  | FMS vs CC | 11.72 (3.19) | 3.22, 20.22 | **0.002** |
|  | HC vs CC | -14.10 (3.19) | -22.6, -5.6 | **<0.001** |

**Key:** CC=clinical control; CFQ=Cognitive Failures Questionnaire; FMS=functional motor symptoms; FS=functional seizures; HC=healthy control

**Supplementary Table 8. WASI-II post-hoc tests**

| **Test Outcome** | **Comparison** | **Mean difference (standard error)** | **Confidence interval (95%)** | **p-value** |  |
| --- | --- | --- | --- | --- | --- |
| ***WASI-II Full Scale IQ^#^*** | FS vs FMS | -2.04 (2.15) | -7.67, 3.59 | 0.78 |  |
|  | FS vs HC | -3.84 (1.68) | -8.25, 0.57 | 0.11 |  |
|  | FS vs CC | -6.36 (1.88) | -11.26, -1.46 | **0.005** |  |
|  | FMS vs HC | -1.80 (1.96) | -6.93, 3.33 | 0.79 |  |
|  | FMS vs CC | -4.32 (2.12) | -9.87, 1.23 | 0.18 |  |
|  | HC vs CC | -2.52 (1.65) | -6.83, 1.79 | 0.42 |  |
| ***WASI-II Vocabulary T scores^*^*** | | FS vs FMS | -2.04 (1.92) | -7.15, 3.07 | 1.00 |
|  | | FS vs HC | -3.84 (1.92) | -8.95, 1.27 | 0.28 |
|  | | FS vs CC | -6.36 (1.92) | -11.47, -1.25 | **0.006** |
|  | | FMS vs HC | -1.80 (1.92) | -6.91, 3.31 | 1.00 |
|  | | FMS vs CC | -4.32 (1.92) | -9.43, 0.79 | 0.15 |
|  | | HC vs CC | -2.52 (1.92) | -7.63, 2.59 | 1.00 |
| ***WASI-II Matrix Reasoning T scores^*^*** | FS vs FMS | 0.96 (1.80) | -3.84, 5.76 | 1.00 |  |
|  | | FS vs HC | -6.14 (1.80) | -10.94, -1.34 | **0.005** |
|  | | FS vs CC | -6.12 (1.80) | -10.92, -1.32 | **0.005** |
|  | | FMS vs HC | -7.10 (1.80) | -11.90, -2.30 | **<0.001** |
|  | | FMS vs CC | -7.08 (1.80) | -11.88, -2.28 | **<0.001** |
|  | | HC vs CC | 0.02 (1.80) | -4.78, 4.82 | 1.00 |

**Key:** CC=clinical control; CFQ=Cognitive Failures Questionnaire; FMS=functional motor symptoms; FS=functional seizures; HC=healthy control; IQ=intelligence quotient; WASI-II=Wechsler Abbreviated Scale of Intelligence – Second edition

*Bonferroni; ^#^Games-Howell

**Supplementary Table 9. WASI-II sensitivity analyses**

| **Adjustment / Test outcome** | **FS** | **FMS** | **CC** | **HC** | **Test statistics** |
| --- | --- | --- | --- | --- | --- |
| ***MSVT fails excluded*** | **M (SD)**  **n=48** | **M (SD)**  **n=46** | **M (SD)**  **n=48** | **M (SD)**  **n=49** | **One-way ANOVA** |
| *Full Scale IQ* | 97.46 (14.58) | 99.04 (13.88) | 108.29 (10.82) | 105.98 (9.60) | *F(3, 101.95)=8.27, **p<0.001**, ω²=0.11 |
| *Vocabulary T Score* | 46.25 (9.58) | 48.76 (12.04) | 52.35  (8.80) | 50.08 (6.86) | *F(3, 101.24)=3.63, **p=0.02,** ω²=0.04 |
| *Matrix Reasoning T Score* | 50.90 (9.61) | 50.17 (8.78) | 56.77  (8.03) | 56.98 (8.44) | F(3, 187)=8.41, **p<0.001,** η²=0.12 |
| ***Outliers winsorised*** | **M (SD)**  **n=48** | **M (SD)**  **n=46** | **M (SD)**  **n=48** | **M (SD)**  **n=49** | **One-way ANOVA** |
| *Full Scale IQ* | 97.42 (14.47) | 99.04 (13.88) | 108.15 (10.39) | 105.98 (9.60) | *F(3, 101.93)=8.39, **p<0.001,** ω²=0.11 |
| *Vocabulary T Score* | 46.48 (9.06) | 48.11 (10.46) | 51.81  (7.33) | 50.08 (6.86) | *F(3, 102.10)=3.70, **p=0.01,** ω²=0.04 |
| *Matrix Reasoning T Score* | 50.88 (8.93) | 50.17 (8.78) | 57.25  (6.44) | 56.98 (8.44) | *F(3, 102.64)=10.55, **p<0.001**, ω²=0.13 |
| ***Education*** | **EMM (SE)**  **n=50** | **EMM (SE)**  **n=50** | **EMM (SE)**  **n=50** | **EMM (SE)**  **n=50** | **ANCOVA** |
| *Full Scale IQ* | 97.46 (1.75) | 99.01 (1.77) | 107.31 (1.76) | 104.17 (1.77) | Group: F(3, 195)=6.48, **p<0.001,** ηp²=0.09 |
|  |  |  |  |  | Education: F(1, 195)=16.87, **p<0.001,** ηp²=0.08 |
| *Vocabulary T Score* | 46.39 (1.32) | 48.85 (1.33) | 51.80  (1.32) | 48.96 (1.33) | Group: F(3, 195)=2.79, **p=0.04,** ηp²=0.04 |
|  |  |  |  |  | Education: F(1, 195)=14.05, **p<0.001,** ηp²=0.07 |
| *Matrix Reasoning T Score* | 50.77 (1.25) | 50.13 (1.27) | 56.15  (1.25) | 55.93 (1.26) | Group: F(3, 195)=6.30, **p<0.001,** ηp²=0.09 |
|  |  |  |  |  | Education: F(1, 195)=9.27, **p=0.003,** ηp²=0.05 |
| ***Age*** | **EMM (SE)**  **n=50** | **EMM (SE)**  **n=50** | **EMM (SE)**  **n=50** | **EMM (SE)**  **n=50** | **ANCOVA** |
| *Full Scale IQ* | 96.80 (1.82) | 97.76 (1.82) | 108.03 (1.82) | 105.35 (1.82) | Group: F(3, 195)=9.31, **p<0.001,** ηp²=0.13 |
|  |  |  |  |  | Age: F(1, 195)=0.06, p=0.82, ηp²=0.00 |
| *Vocabulary T Score* | 45.94 (1.36) | 47.98 (1.36) | 52.30 (1.36) | 49.78 (1.36) | Group: F(3, 195)=3.96, **p=0.009,** ηp²=0.06 |
|  |  |  |  |  | Age: F(1, 195)=0.03, p=0.96, ηp²=0.00 |
| *Matrix Reasoning T Score* | 50.43 (1.27) | 49.50 (1.28) | 56.52 (1.27) | 56.53 (1.27) | Group: F(3, 195)=8.92, **p<0.001,** ηp²=0.12 |
|  |  |  |  |  | Age: F(1, 195)=0.57, p=0.45, ηp²=0.003 |
| ***Gender*** | **EMM (SE)**  **n=50** | **EMM (SE)**  **n=50** | **EMM (SE)**  **n=50** | **EMM (SE)**  **n=50** | **ANCOVA** |
| *Full Scale IQ* | 96.80 (1.82) | 97.74 (1.82) | 108.03 (1.82) | 105.36 (1.82) | Group: F(3, 195)=9.35, **p<0.001,** ηp²=0.13 |
|  |  |  |  |  | Gender: F(1, 195)=0.01, p=0.94, ηp²=0.000 |
| *Vocabulary T Score* | 45.95 (1.36) | 47.96 (1.36) | 52.33 (1.36) | 49.76 (1.36) | Group: F(3, 195)=3.98, **p=0.009**, ηp²=0.06 |
|  |  |  |  |  | Gender: F(1, 195)=0.18, p=0.68, ηp²=0.001 |
| *Matrix Reasoning T Score* | 50.42 (1.28) | 49.46 (1.28) | 56,54 (1.28) | 56.56 (1.28) | Group: F(3, 195)=9.05, **p<0.001**, ηp²=0.12 |
|  |  |  |  |  | Gender: F(1, 195)=0.00, p=0.97, ηp²=0.000 |
| ***Psychotropic medication (FS/FMS/CC groups)*** | **EMM (SE)**  **n=50** | **EMM (SE)**  **n=50** | **EMM (SE)**  **n=50** |  | **ANCOVA** |
| *Full Scale IQ* | 96.99  (1.92) | 97.96  (1.92) | 107.62  (1.94) | - | Group: F(2, 146)=9.09, **p<0.001,** ηp²=0.11 |
|  |  |  |  |  | Psychotropics: F(1, 146)=1.68, p=0.20, ηp²=0.01 |
| *Vocabulary T Score* | 46.05  (1.46) | 48.11  (1.46) | 52.05  (1.47) | - | Group: F(2, 146)=4.25, **p=0.02,** ηp²=0.06 |
|  |  |  |  |  | Psychotropics: F(1, 146)=1.01, p=0.32, ηp²=0.01 |
| *Matrix Reasoning T Score* | 50.50  (1.28) | 49.55  (1.29) | 56.37  (1.30) | - | Group: F(2, 146)=7.98, **p<0.001,** ηp²=0.10 |
|  |  |  |  |  | Psychotropics: F(1, 146)=0.64, p=0.43, ηp²=0.00 |

**Key:** ANCOVA=Analysis of Covariance; ANOVA=Analysis of Variance; CC=clinical control; EMM=estimated marginal means; FMS=functional motor symptoms; FS=functional seizures; HC=healthy control; IQ=Intelligence Quotient; M=mean; MSVT=Medical Symptom Validity Test; SD=standard deviation; SE=standard error

**Supplementary Table 10. Motor Screening and Reaction Time Test sensitivity analyses**

| **Adjustment / Test outcome** | **FS** | **FMS** | **CC** | **HC** | **Test statistics** |
| --- | --- | --- | --- | --- | --- |
| ***MSVT fails excluded*** | **M (SD)**  **n=48** | **M (SD)**  **n=46** | **M (SD)**  **n=48** | **M (SD)**  **n=49** | **ANOVA** |
| *MST Mean Motor Latency* | 895.99 (322.11) | 1009.87  (357.10) | 758.85 (176.84) | 783.97 (188.62) | *F(3, 99.69)=7.49, **p<0.001**, ω²=0.11 |
| *RTT Mean Reaction Time* | 409.02 (53.32) | 445.79 (117.81) | 369.28 (39.84) | 355.86 (32.18) | *F(3, 98.04)=17.67, **p<0.001**, ω²=0.20 |
| *RTT Mean Movement Time* | 293.34 (84.23) | 285.11 (85.71) | 245.04 (49.87) | 247.17 (57.34) | *F(3, 100.93)=5.94, **p<0.001**, ω²=0.07 |
| ***Outliers winsorised*** | **M (SD)**  **n=48** | **M (SD)**  **n=46** | **M (SD)**  **n=48** | **M (SD)**  **n=49** | **ANOVA** |
| *MST Mean Motor Latency* | 869.96 (237.85) | 1004.60 (342.31) | 752.88 (161.55) | 776.34 (168.30) | *F(3, 100.33)=8.42, **p<0.001**, ω²=0.13 |
| *RTT Mean Reaction Time* | 407.05 (47.29) | 440.90 (102.31) | 369.28 (39.84) | 355.25 (30.56) | *F(3, 98.44)=20.42, **p<0.001**, ω²=0.22 |
| *RTT Mean Movement Time* | 287.79 (61.89) | 283.78 (82.14) | 244.09 (47.45) | 247.17 (57.34) | *F(3, 101.82)=7.03, **p<0.001**, ω²=0.08 |
| ***Education*** | **EMM (SE)**  **n=50** | **EMM (SE)**  **n=50** | **EMM (SE)**  **n=50** | **EMM (SE)**  **n=50** | **ANCOVA** |
| *MST Mean Motor Latency* | 908.92  (38.66) | 1007.06  (39.10) | 765.29  (38.70) | 792.38  (39.03) | Group: F(3, 195)=7.83, **p<0.001,** ηp²=0.11 |
|  |  |  |  |  | Education: F(1, 195)=1.73, p=0.19, ηp²=0.01 |
| *RTT Mean Reaction Time* | 419.03  (12.07) | 460.60  (12.21) | 373.53  (12.08) | 361.65  (12.19) | Group: F(3, 195)=13.20, **p<0.001,** ηp²=0.17 |
|  |  |  |  |  | Education: F(1, 195)=6.82, **p=0.01,** ηp²=0.03 |
| *RTT Mean Movement Time* | 296.62  (10.21) | 291.17  (10.33) | 244.43  (10.22) | 246.90  (10.31) | Group: F(3, 195)=7.08, **p<0.001,** ηp²=0.10 |
|  |  |  |  |  | Education: F(1, 195)=0.22, p=0.64, ηp²=0.00 |
| ***Age*** | **EMM (SE)**  **n=50** | **EMM (SE)**  **n=50** | **EMM (SE)**  **n=50** | **EMM (SE)**  **n=50** | **ANCOVA** |
| *MST Mean Motor Latency* | 914.26 (38.17) | 1019.42 (38.20) | 758.57 (38.18) | 781.39 (38.19) | Group: F(3, 195)=10.17, **p<0.001,** ηp²=0.14 |
|  |  |  |  |  | Age: F(1, 195)=5.19, **p=0.02**, ηp²=0.03 |
| *RTT Mean Reaction Time* | 422.21 (11.93) | 467.61 (11.94) | 369.66 (11.93) | 355.33 (11.94) | Group: F(3, 195)=18.51, **p<0.001,** ηp²=0.22 |
|  |  |  |  |  | Age: F(1, 195)=9.79, **p=0.002**, ηp²=0.05 |
| *RTT Mean Movement Time* | 297.33 (9.88) | 293.33 (9.89) | 243.35 (9.88) | 245.12 (9.88) | Group: F(3, 195)=8.93, **p<0.001,** ηp²=0.12 |
|  |  |  |  |  | Age: F(1, 195)=11.81, **p<0.001**, ηp²=0.06 |
| ***Gender*** | **EMM (SE)**  **n=50** | **EMM (SE)**  **n=50** | **EMM (SE)**  **n=50** | **EMM (SE)**  **n=50** | **ANCOVA** |
| *MST Mean Motor Latency* | 914.71 (38.42) | 1014.10 (38.43) | 762.84 (38.45) | 782.00 (38.43) | Group: F(3, 195)=9.48, **p<0.001,** ηp²=0.13 |
|  |  |  |  |  | Gender: F(1, 195)=2.63, p=0.11, ηp²=0.01 |
| *RTT Mean Reaction Time* | 422.07 (12.21) | 465.88 (12.22) | 370.72 (12.22) | 356.15 (12.22) | Group: F(3, 195)=16.88, **p<0.001,** ηp²=0.21 |
|  |  |  |  |  | Gender: F(1, 195)=0.54, p=0.46, ηp²=0.003 |
| *RTT Mean Movement Time* | 296.88 (10.16) | 292.29 (10.16) | 243.56 (10.16) | 246.40 (10.16) | Group: F(3, 195)=8.00, **p<0.001,** ηp²=0.11 |
|  |  |  |  |  | Gender: F(1, 195)=0.78, p=0.38, ηp²=0.004 |
| ***WASI-II FSIQ-2*** | **EMM (SE)**  **n=50** | **EMM (SE)**  **n=50** | **EMM (SE)**  **n=50** | **EMM (SE)**  **n=50** | **ANCOVA** |
| *MST Mean Motor Latency* | 883.81 (38.01) | 991.69 (37.75) | 794.85 (38.28) | 803.30 (37.57) | Group: F(3, 195)=5.51, **p=0.001,** ηp²=0.08 |
|  |  |  |  |  | WASI-II FSIQ-2: F(1, 195)=15.32, **p<0.001,** ηp²=0.07 |
| *RTT Mean Reaction Time* | 412.50 (12.02) | 458.47 (11.94) | 381.30 (12.01) | 362.54 (11.88) | Group: F(3, 195)=11.68, **p<0.001,** ηp²=0.15 |
|  |  |  |  |  | WASI-II FSIQ-2: F(1, 195)=15.30, **p<0.001,** ηp²=0.07 |
| *RTT Mean Movement Time* | 291.43 (10.19) | 287.40 (10.12) | 250.52 (10.26) | 249.78 (10.07) | Group: F(3, 195)=4.56, **p=0.004,** ηp²=0.07 |
|  |  |  |  |  | WASI-II FSIQ-2: F(1, 195)=7.63, **p=0.006,** ηp²=0.04 |
| ***Psychotropic medication***  ***(FS/FMS/CC groups)*** | **EMM (SE)**  **n=50** | **EMM (SE)**  **n=50** | **EMM (SE)**  **n=50** |  | **ANCOVA** |
| *MST Mean Motor Latency* | 904.62  (41.05) | 1005.78  (41.09) | 779.30  (41.50) | - | Group: F(2, 146)=7.42, **p<0.001,** ηp²=0.09 |
|  |  |  |  |  | Psychotropics: F(1, 146)=7.73, **p=0.006,** ηp²=0.05 |
| *RTT Mean Reaction Time* | 419.75  (13.74) | 463.69  (13.75) | 374.94  (13.89) | - | Group: F(2, 146)=10.16, **p<0.001,** ηp²=0.12 |
|  |  |  |  |  | Psychotropics: F(1, 146)=4.00, **p=0.048**, ηp²=0.03 |
| *RTT Mean Movement Time* | 296.04  (10.76) | 290.85  (10.77) | 246.13  (10.88) | - | Group: F(2, 146)=6.28, **p=0.002,** ηp²=0.08 |
|  |  |  |  |  | Psychotropics: F(1, 146)=1.44, p=0.23, ηp²=0.01 |

**Key:** ANCOVA=Analysis of Covariance; ANOVA=Analysis of Variance; CC=clinical control; EMM=estimated marginal means; FMS=functional motor symptoms; FS=functional seizures; HC=healthy control; M=mean; MST=Motor Screening Test; RTT=Reaction Time Test; SD=standard deviation; SE=standard error; WASI-II FSIQ-2=Wechsler Abbreviated Scale of Intelligence – Second edition Full Scale Intelligence Quotient – 2 subtest

**Supplementary Table 11. Motor screening and reaction time Games-Howell post-hoc tests**

| **Test outcome** | **Comparison** | **Mean difference (standard error)** | **Confidence interval (95%)** | **p-value** |  |
| --- | --- | --- | --- | --- | --- |
| ***MST Mean Motor Latency*** | FS vs FMS | -102.49 (67.96) | -280.15, 75.16 | 0.44 |  |
|  | FS vs HC | 129.60 (53.29) | -10.31, 269.52 | 0.08 |  |
|  | FS vs CC | 153.43 (52.71) | 14.95, 291.91 | **0.02** |  |
|  | FMS vs HC | 232.10 (56.33) | 84.06, 380.13 | **<0.001** |  |
|  | FMS vs CC | 255.92 (55.79) | 109.23, 402.61 | **<0.001** |  |
|  | HC vs CC | 23.82 (36.51) | -71.60, 119.25 | 0.91 |  |
| ***RTT Mean Reaction Time*** | | FS vs FMS | -44.26 (23.26) | -105.31, 16.78 | 0.24 |
|  | | FS vs HC | 65.47 (12.74) | 31.86, 99.08 | **<0.001** |
|  | | FS vs CC | 51.57 (13.22) | 16.79, 86.36 | **0.001** |
|  | | FMS vs HC | 109.73 (20.50) | 55.39, 164.08 | **<0.001** |
|  | | FMS vs CC | 95.84 (20.80) | 40.78, 150.89 | **<0.001** |
|  | | HC vs CC | -13.90 (7.33) | -33.08, 5.28 | 0.24 |
| ***RTT Mean Movement Time*** | FS vs FMS | 5.04 (17.19) | -39.89, 49.97 | 0.99 |  |
|  | | FS vs HC | 50.93 (14.55) | 12.80, 89.07 | **0.004** |
|  | | FS vs CC | 53.10 (14.04) | 16.25, 89.95 | **0.002** |
|  | | FMS vs HC | 45.89 (14.65) | 7.49, 84.29 | **0.01** |
|  | | FMS vs CC | 48.06 (14.15) | 10.93, 85.18 | **0.006** |
|  | | HC vs CC | 2.166 (10.79) | -26.04, 30.37 | 0.10 |

**Key:** CC=clinical control; FMS=functional motor symptoms; FS=functional seizures; HC=healthy control; MST=Motor Screening Test; RTT=Reaction Time Test

^#^Games-Howell

**Supplementary Table 12. Rapid Visual Information Processing sensitivity analyses**

| **Adjustment / Test outcome** | **FS** | **FMS** | **CC** | **HC** | **Test statistics** |
| --- | --- | --- | --- | --- | --- |
| ***MSVT fails excluded*** | **M (SD)**  **n=46** | **M (SD)**  **n=46** | **M (SD)**  **n=47** | **M (SD)**  **n=48** | **ANOVA** |
| *RVIP Ability* | 0.89 (0.04) | 0.88 (0.05) | 0.93 (0.05) | 0.92 (0.05) | F(3, 183)=11.50, **p<0.001**, η²=0.16 |
| *RVIP Total Misses* | 22.11 (8.32) | 24.67 (10.01) | 15.06 (9.08) | 16.83 (9.24) | F(3, 183)=11.10, **p<0.001**, η²=0.15 |
| *RVIP Probability of Hit* | 0.59 (0.15) | 0.54 (0.19) | 0.72 (0.17) | 0.69 (0.17) | F(3, 183)=11.10, **p<0.001**, η²=0.15 |
| *RVIP Median Response Latency* | 458.79 (77.10) | 528.47 (173.83) | 454.98 (82.02) | 452.82 (70.10) | *F(3, 98.82)=2.57, p=0.06, ω²=0.06 |
| ***Outliers winsorised*** | **M (SD)**  **n=46** | **M (SD)**  **n=46** | **M (SD)**  **n=47** | **M (SD)**  **n=48** | **ANOVA** |
| *RVIP Ability* | 0.89 (0.04) | 0.88 (0.05) | 0.93 (0.04) | 0.92 (0.04) | F(3, 183)=11.93, **p<0.001**, η²=0.16 |
| *RVIP Total Misses* | 22.11 (8.32) | 24.67 (10.01) | 15.06 (9.08) | 16.83 (9.24) | F(3, 183)=11.10, **p<0.001**, η²=0.15 |
| *RVIP Probability of Hit* | 0.59 (0.15) | 0.54 (0.19) | 0.72 (0.17) | 0.69 (0.17) | F(3, 183)=11.10, **p<0.001**, η²=0.15 |
| *RVIP Median Response Latency* | 456.64 (70.92) | 511.97 (118.46) | 454.98 (82.02) | 451.77 (67.02) | *F(3, 98.84)=3.24, **p=0.03**, ω²=0.06 |
| ***Education*** | **EMM (SE)**  **n=47** | **EMM (SE)**  **n=50** | **EMM (SE)**  **n=49** | **EMM (SE)**  **n=49** | **ANCOVA** |
| *RVIP Ability* | 0.89  (0.01) | 0.88  (0.01) | 0.93  (0.01) | 0.91  (0.01) | Group: F(3, 190)=10.19, **p<0.001**, η²=0.14 |
|  |  |  |  |  | Education: F(1, 190)=6.41, **p=0.01**, η²=0.03 |
| *RVIP Total Misses* | 22.16  (1.35) | 24.51  (1.32) | 15.41  (1.32) | 17.57  (1.33) | Group: F(3, 190)=9.39, **p<0.001**, η²=0.13 |
|  |  |  |  |  | Education: F(3, 190)=4.87, **p=0.03**, η²=0.03 |
| *RVIP Probability of Hit* | 0.59 (0.03) | 0.55  (0.02) | 0.72  (0.02) | 0.68  (0.03) | Group: F(3, 190)=9.39, **p<0.001**, η²=0.13 |
|  |  |  |  |  | Education: F(1, 190)=4.87, **p=0.03**, η²=0.03 |
| *RVIP Median Response Latency* | 468.09  (16.81) | 546.22  (16.50) | 450.98  (16.51) | 454.28  (16.61) | Group: F(3, 190)=7.18, **p<0.001**, η²=0.10 |
|  |  |  |  |  | Education: F(1, 190)=1.10, p=0.30, η²=0.01 |
| ***Age*** | **EMM (SE)**  **n=47** | **EMM (SE)**  **n=50** | **EMM (SE)**  **n=49** | **EMM (SE)**  **n=49** | **ANCOVA** |
| *RVIP Ability* | 0.89 (0.01) | 0.88 (0.01) | 0.93 (0.01) | 0.92 (0.01) | Group: F(3, 190)=13.21, **p<0.001**, η²=0.17 |
|  |  |  |  |  | Age: F(1, 190)=0.96, p=0.33, η²=0.005 |
| *RVIP Total Misses* | 22.43 (1.35) | 25.00 (1.31) | 15.09 (1.32) | 17.14 (1.33) | Group: F(3, 190)=12.03, **p<0.001**, η²=0.16 |
|  |  |  |  |  | Age: F(1, 190)=1.46, p=0.23, η²=0.008 |
| *RVIP Probability of Hit* | 0.59 (0.03) | 0.54 (0.02) | 0.72 (0.03) | 0.68 (0.03) | Group: F(3, 190)=12.03, **p<0.001**, η²=0.16 |
|  |  |  |  |  | Age: F(1, 190)=1.46, p=0.23, η²=0.008 |
| *RVIP Median Response Latency* | 467.00 (16.47) | 544.23 (15.98) | 452.26 (16.14) | 456.07 (16.14) | Group: F(3, 190)=7.31, **p<0.001**, η²=0.10 |
|  |  |  |  |  | Age: F(1, 190)=7.27, **p=0.008**, η²=0.04 |
| ***Gender*** | **EMM (SE)**  **n=47** | **EMM (SE)**  **n=50** | **EMM (SE)**  **n=49** | **EMM (SE)**  **n=49** | **ANCOVA** |
| *RVIP Ability* | 0.89 (0.01) | 0.88 (0.01) | 0.93 (0.01) | 0.92 (0.01) | Group: F(3, 190)=13.244, **p<0.001**, η²=0.18 |
|  |  |  |  |  | Gender: F(1, 190)=0.83, p=0.36, η²=0.004 |
| *RVIP Total Misses* | 22.39 (1.36) | 25.09 (1.31) | 15.02 (1.33) | 17.15 (1.33) | Group: F(3, 190)=12.25, **p<0.001**, η²=0.16 |
|  |  |  |  |  | Gender: F(1, 190)=0.73, p=0.39, η²=0.004 |
| *RVIP Probability of Hit* | 0.59 (0.03) | 0.54 (0.02) | 0.72 (0.03) | 0.68 (0.03) | Group: F(3, 190)=12.25, **p<0.001**, η²=0.16 |
|  |  |  |  |  | Gender: F(1, 190)=0.73, p=0.39, η²=0.004 |
| *RVIP Median Response Latency* | 466.56 (16.81) | 542.96 (16.29) | 453.12 (16.45) | 456.95 (16.45) | Group: F(3, 190)=6.74, **p<0.001**, η²=0.096 |
|  |  |  |  |  | Gender: F(1, 190)=0.03, p=0.86, η²=0.00 |
| ***WASI-II FSIQ-2*** | **EMM**  **(SE)**  **n=47** | **EMM (SE)**  **n=50** | **EMM**  **(SE)**  **n=49** | **EMM (SE)**  **n=49** | **ANCOVA** |
| *RVIP Ability* | 0.90 (0.01) | 0.88 (0.01) | 0.92 (0.01) | 0.91 (0.01) | Group: F(3, 190)=6.59, **p<0.001** ηp²=0.09 |
|  |  |  |  |  | WASI-II FSIQ-2: F(1, 190)=41.00, **p<0.001,** ηp²=0.18 |
| *RVIP Total Misses* | 21.13 (1.30) | 23.93 (1.25) | 16.59 (1.28) | 17.97 (1.26) | Group: F(3, 190)=6.30, **p<0.001**, ηp²=0.09 |
|  |  |  |  |  | WASI-II FSIQ-2: F(1, 190)=27.02, **p<0.001,** ηp²=0.13 |
| *RVIP Probability of Hit* | 0.61 (0.02) | 0.56 (0.02) | 0.69 (0.02) | 0.67 (0.02) | Group: F(3, 190)=6.30, **p<0.001**, ηp²=0.09 |
|  |  |  |  |  | WASI-II FSIQ-2: F(1, 190)=27.02, **p<0.001,** ηp²=0.13 |
| *RVIP Median Response Latency* | 462.48 (17.05) | 539.76 (16.56) | 457.57 (16.83) | 459.66 (16.53) | Group: F(3, 190)=5.78, **p<0.001,** ηp²=0.08 |
|  |  |  |  |  | WASI-II FSIQ-2: F(1, 190)=1.38, p=0.24, ηp²=0.01 |
| ***Psychotropic medication***  ***(FS/FMS/CC groups)*** | **EMM (SE)**  **n=47** | **EMM (SE)**  **n=50** | **EMM (SE)**  **n=49** |  | **ANCOVA** |
| *RVIP Ability* | 0.89  (0.01) | 0.88 (0.01) | 0.93 (0.01) | - | Group: F(2, 142)=15.45, **p<0.001**, ηp²=0.18 |
|  |  |  |  |  | Psychotropics: F(1, 142)=0.17, p=0.68, ηp²=0.00 |
| *RVIP Total Misses* | 22.43 (1.36) | 25.02 (1.32) | 15.10 (1.34) | - | Group: F(2, 142)=14.53, **p<0.001**, ηp²=0.17 |
|  |  |  |  |  | Psychotropics: F(1, 142)=0.04, p=0.84, ηp²=0.00 |
| *RVIP Probability of Hit* | 0.59 (0.03) | 0.54 (0.02) | 0.72 (0.03) | - | Group: F(2, 142)=14.53, **p<0.001**, ηp²=0.17 |
|  |  |  |  |  | Psychotropics: F(1, 142)=0.04, p=0.84, ηp²=0.00 |
| *RVIP Median Response Latency* | 464.91 (18.29) | 541.24 (17.76) | 456.33 (18.09) | - | Group: F(2, 142)=6.80, **p=0.002**, ηp²=0.09 |
|  |  |  |  |  | Psychotropics: F(1, 142)=1.35, p=0.25, ηp²=0.01 |
| ***MST Mean Motor Latency*** | **EMM (SE)**  **n=47** | **EMM (SE)**  **n=50** | **EMM (SE)**  **n=49** | **EMM (SE)**  **n=49** | **ANCOVA** |
| *RVIP Ability* | 0.89 (0.01) | 0.88 (0.01) | 0.93 (0.01) | 0.92 (0.01) | Group: F(3, 190)=10.28, **p<0.001**, ηp²=0.14 |
|  |  |  |  |  | MST MML: F(1, 190)=1.19, p=0.28, ηp²=0.01 |
| *RVIP Total Misses* | 22.35 (1.36) | 24.50 (1.36) | 15.31 (1.35) | 17.30 (1.34) | Group: F(3, 190)=9.47, **p<0.001**, ηp²=0.13 |
|  |  |  |  |  | MST MML: F(1, 190)=0.89, p=0.35, ηp²=0.01 |
| *RVIP Probability of Hit* | 0.59 (0.03) | 0.54 (0.03) | 0.72 (0.03) | 0.68 (0.03) | Group: F(3, 190)=9.47, **p<0.001**, ηp²=0.13 |
|  |  |  |  |  | MST MML: F(1, 190)=0.89, p=0.35, ηp²=0.01 |
| *RVIP Median Response Latency* | 462.33 (16.41) | 529.14 (16.46) | 463.09 (16.33) | 465.13 (16.22) | Group: F(3, 190)=3.88, **p=0.01**, ηp²=0.06 |
|  |  |  |  |  | MST MML: F(1, 190)=10.06, **p=0.002**, ηp²=0.05 |
| ***RTT Mean Reaction Time*** | **EMM (SE)**  **n=47** | **EMM (SE)**  **n=50** | **EMM (SE)**  **n=49** | **EMM (SE)**  **n=49** | **ANCOVA** |
| *RVIP Ability* | 0.89 (0.01) | 0.88 (0.01) | 0.92 (0.01) | 0.91 (0.01) | Group: F(3, 190)=7.13, **p<0.001**, ηp²=0.10 |
|  |  |  |  |  | RTT MRT: F(1, 190)=8.38, **p=0.004**, ηp²=0.04 |
| *RVIP Total Misses* | 22.15 (1.34) | 23.72 (1.38) | 15.73 (1.33) | 18.06 (1.35) | Group: F(3, 190)=6.55, **p<0.001**, ηp²=0.09 |
|  |  |  |  |  | RTT MRT: F(1, 190)=7.06, **p=0.009**, ηp²=0.04 |
| *RVIP Probability of Hit* | 0.59 (0.03) | 0.56 (0.03) | 0.71 (0.03) | 0.67 (0.03) | Group: F(3, 190)=6.55, **p<0.001**, ηp²=0.09 |
|  |  |  |  |  | RTT MRT: F(1, 190)=7.06, **p=0.009**, ηp²=0.04 |
| *RVIP Median Response Latency* | 457.78 (15.15) | 505.50 (15.68) | 472.21 (15.06) | 484.49 (15.35) | Group: F(3, 190)=1.77, p=0.15, ηp²=0.03 |
|  |  |  |  |  | RTT MRT: F(1, 190)=44.79, **p<0.001**, ηp²=0.19 |
| ***RTT Mean Movement Time*** | **EMM (SE)**  **n=47** | **EMM (SE)**  **n=50** | **EMM (SE)**  **n=49** | **EMM (SE)**  **n=49** | **ANCOVA** |
| *RVIP Ability* | 0.89 (0.01) | 0.88 (0.01) | 0.93 (0.01) | 0.92 (0.01) | Group: F(3, 190)=11.62, **p<0.001**, ηp²=0.16 |
|  |  |  |  |  | RTT MMT: F(1, 190)=0.09, p=0.77, ηp²=0.00 |
| *RVIP Total Misses* | 22.42 (1.38) | 25.02 (1.33) | 15.09 (1.35) | 17.13 (1.35) | Group: F(3, 190)=10.78, **p<0.001**, ηp²=0.15 |
|  |  |  |  |  | RTT MMT: F(1, 190)=0.01, p=0.91, ηp²=0.00 |
| *RVIP Probability of Hit* | 0.59 (0.03) | 0.54 (0.03) | 0.72 (0.03) | 0.68 (0.03) | Group: F(3, 190)=10.78, **p<0.001**, ηp²=0.15 |
|  |  |  |  |  | RTT MMT: F(1, 190)=0.01, p=0.91, ηp²=0.00 |
| *RVIP Median Response Latency* | 461.59 | 539.17 | 457.54 | 461.16 | Group: F(3, 190)=5.73, **p<0.001**, ηp²=0.08 |
|  |  |  |  |  | RTT MMT: F(1, 190)=2.18, p=0.14, ηp²=0.01 |

**Key:** ANCOVA=Analysis of Covariance; ANOVA=Analysis of Variance; CC=clinical control; EMM=estimated marginal means; FMS=functional motor symptoms; FS=functional seizures; HC=healthy control; M=mean; MST MML=Motor Screening Test Mean Motor Latency; MSVT=Medical Symptom Validity Test; RTT MMT=Reaction Time Test Mean Movement Time; RTT MRT=Reaction Time Test Mean Reaction Time; RVIP=Rapid Visual Information Processing; SD=standard deviation; SE=standard error; WASI-II FSIQ-2=Wechsler Abbreviated Scale of Intelligence – Second edition Full Scale Intelligence Quotient – 2 subtest *Welch’s ANOVA

**Supplementary Table 13. Rapid Visual Information Processing Bonferroni post-hoc tests**

| **Test outcome** | **Comparison** | **Mean difference (standard error)** | **Confidence interval (95%)** | **p-value** |  |
| --- | --- | --- | --- | --- | --- |
| ***RVIP Ability*** | FS vs FMS | 0.01 (0.01) | -0.01, 0.04 | 0.92 |  |
|  | FS vs HC | -0.03 (0.01) | -0.05, -0.00 | **0.02** |  |
|  | FS vs CC | -0.04 (0.01) | -0.06, -0.01 | **<0.001** |  |
|  | FMS vs HC | -0.04 (0.01) | -0.07, -0.02 | **<0.001** |  |
|  | FMS vs CC | -0.05 (0.01) | -0.08, -0.03 | **<0.001** |  |
|  | HC vs CC | -0.01 (0.01) | -0.04, 0.01 | 1.00 |  |
| ***RVIP Total Misses*** | | FS vs FMS | -2.59 (1.89) | -7.62, 2.43 | 1.00 |
|  | | FS vs HC | 5.35 (1.90) | 0.29, 10.40 | **0.03** |
|  | | FS vs CC | 7.39 (1.90) | 2.33, 12.44 | **<0.001** |
|  | | FMS vs HC | 7.94 (1.90) | 2.96, 12.91 | **<0.001** |
|  | | FMS vs CC | 9.98 (1.90) | 5.01, 14.95 | **<0.001** |
|  | | HC vs CC | 2.04 (1.90) | -2.96, 7.04 | 1.00 |
| ***RVIP Probability of Hit*** | FS vs FMS | 0.05 (0.03) | -0.05, 0.14 | 1.00 |  |
|  | | FS vs HC | -0.10 (0.04) | -0.19, -0.01 | **0.03** |
|  | | FS vs CC | -0.14 (0.04) | -0.23, -0.04 | **<0.001** |
|  | | FMS vs HC | -0.15 (0.03) | -0.24, -0.05 | **<0.001** |
|  | | FMS vs CC | -0.18 (0.03) | -0.28, -0.09 | **<0.001** |
|  | | HC vs CC | -0.04 (0.03) | -0.13, 0.05 | 1.00 |

**Key:** CC=clinical control; FMS=functional motor symptoms; FS=functional seizures; HC=healthy control; RVIP=Rapid Visual Information Processing

**Supplementary Table 14. Intra-Extra Dimensional Set-Shift sensitivity analyses**

| **Adjustment / Test outcome** | **FS** | **FMS** | **CC** | **HC** | **Test statistics** |
| --- | --- | --- | --- | --- | --- |
| ***MSVT fails excluded*** | **M (SD)**  **n=40** | **M (SD)**  **n=36** | **M (SD)**  **n=42** | **M (SD)**  **n=43** | **ANOVA** |
| *IEDSS Total Errors* | 18.20 (11.79) | 19.14 (10.98) | 11.88 (4.41) | 14.72 (9.75) | *F(3, 77.10)=7.27, **p<0.001**, ω²=0.07 |
| *IEDSS Adjusted Errors* | 35.50 (44.98) | 31.76 (28.42) | 19.40 (22.21) | 27.16 (39.47) | *F(3, 100.70)=2.73, p=0.05, ω²=0.01 |
| *IEDSS Pre-Extra-Dimensional Shift Errors* | 12.65  (10.73) | 12.07  (11.00) | 7.54  (6.84) | 10.41  (9.23) | *F(3, 101.50)=3.55, **p=0.02**, ω²=0.03 |
| *IEDSS Total Trials Completed* | 85.80 (23.98) | 85.94 (20.75) | 72.69 (9.60) | 77.21 (18.34) | *F(3, 79.00)=6.59, **p<0.001**, ω²=0.07 |
| *IEDSS Response Latency* | 131119.23 (56928.56) | 138304.19 (84188.24) | 92543.05 (32042.95) | 106542.02 (37307.06) | *F(3, 80.94)=6.71, **p<0.001**, ω²=0.08 |
| ***Outliers winsorised*** | **M (SD)**  **n=40** | **M (SD)**  **n=36** | **M (SD)**  **n=42** | **M (SD)**  **n=43** | **ANOVA** |
| *IEDSS Total Errors* | 17.85 (10.84) | 18.69 (9.60) | 11.76 (4.11) | 14.19 (7.45) | *F(3, 78.55)=8.15, **p<0.001**, ω²=0.09 |
| *IEDSS Adjusted Errors* | 34.00 (40.39) | 30.35 (24.64) | 17.77 (16.35) | 22.29 (22.91) | *F(3, 100.03)=4.14, **p=0.008**, ω²=0.04 |
| *IEDSS Pre-Extra-Dimensional Shift Errors* | 12.35  (9.90) | 11.50  (8.63) | 6.52  (2.53) | 9.10  (5.56) | *F(3, 90.50)=10.74, **p<0.001**, ω²=0.08 |
| *IEDSS Total Trials Completed* | 85.45 (23.07) | 85.06 (18.14) | 72.69 (9.60) | 75.56 (12.44) | *F(3, 81.24)=6.80, **p<0.001**, ω²=0.09 |
| *IEDSS Response Latency* | 129992.65 (54220.01) | 127800.11 (51374.59) | 91591.74 (28872.71) | 104408.47 (31201.39) | *F(3, 82.21)=8.08, **p<0.001**, ω²=0.11 |
| ***Education*** | **EMM (SE)**  **n=40** | **EMM (SE)**  **n=39** | **EMM (SE)**  **n=43** | **EMM (SE)**  **n=44** | **ANCOVA** |
| *IEDSS Total Errors* | 18.38  (1.53) | 19.22  (1.55) | 11.67  (1.48) | 14.98  (1.46) | Group: F(3, 161)=5.07, **p=0.002**, ηp²=0.09 |
|  |  |  |  |  | Education: F(1, 161)=0.69, p=0.41 ηp²=0.00 |
| *IEDSS Adjusted Errors* | 39.33  (5.23) | 31.97  (5.29) | 20.62  (5.23) | 28.06  (5.28) | Group: F(3, 195)=2.18, p=0.09, ηp²=0.03 |
|  |  |  |  |  | Education: F(1, 195)=0.81, p=0.37, ηp²=0.00 |
| *IEDSS Pre-Extra-Dimensional Shift Errors* | 13.14  (1.41) | 12.55  (1.42) | 7.92  (1.41) | 10.75  (1.42) | Group: F(3, 195)=2.69, **p=0.048**, ηp²=0.04 |
|  |  |  |  |  | Education: F(1, 195)=2.00, p=0.16, ηp²=0.01 |
| *IEDSS Total Trials Completed* | 86.11  (2.99) | 86.34  (3.02) | 72.25  (2.88) | 77.49  (2.84) | Group: F(3, 161)=5.28, **p=0.002**, ηp²=0.09 |
|  |  |  |  |  | Education: F(1, 161)=0.52, p=0.47, ηp²=0.00 |
| *IEDSS Response Latency* | 130817.40 (8719.83) | 136426.12  (8818.62) | 91941.75  (8404.06) | 107387.4  (8293.94) | Group: F(3, 161)=5.58, **p<0.001**, ηp²=0.09 |
|  |  |  |  |  | Education: F(1, 161)=0.06, p=0.81, ηp²=0.00 |
| ***Age*** | **EMM (SE)**  **n=50** | **EMM (SE)**  **n=50** | **EMM (SE)**  **n=50** | **EMM (SE)**  **n=50** | **ANCOVA** |
| *IEDSS Total Errors* | 18.20  (1.51) | 19.13 (1.52) | 11.84 (1.45) | 15.07 (1.44) | Group: F(3, 161)=5.00, **p=0.002**, ηp²=0.09 |
|  |  |  |  |  | Age: F(1, 161)=2.89, p=0.09, ηp²=0.02 |
| *IEDSS Adjusted Errors* | 39.90  (5.06) | 33.48 (5.07) | 19.83 (5.06) | 26.77 (5.06) | Group: F(3, 195)=2.91, **p=0.04**, ηp²=0.04 |
|  |  |  |  |  | Age: F(1, 195)=11.98, **p<0.001**, ηp²=0.06 |
| *IEDSS Pre-Extra-Dimensional Shift Errors* | 13.34  (1.40) | 13.00 (1.40) | 7.68  (1.40) | 10.35 (1.40) | Group: F(3, 195)=3.56, **p=0.02**, ηp²=0.05 |
|  |  |  |  |  | Age: F(1, 195)=3.10, p=0.08, ηp²=0.02 |
| *IEDSS Total Trials Completed* | 85.80  (2.95) | 86.15 (2.99) | 72.53 (2.84) | 77.67 (2.81) | Group: F(3, 161)=5.22, **p=0.002**, ηp²=0.09 |
|  |  |  |  |  | Age: F(1, 161)=1.29, p=0.26, ηp²=0.008 |
| *IEDSS Response Latency* | 131124.15 (8422.46) | 137406.48 (8533.29) | 91645.83 (8123.35) | 106528.83 (8033.35) | Group: F(3, 161)=6.63, **p<0.001**, ηp²=0.11 |
|  |  |  |  |  | Age: F(1, 161)=8.05, **p=0.005**, ηp²=0.05 |
| ***Gender*** | **EMM (SE)**  **n=50** | **EMM (SE)**  **n=50** | **EMM (SE)**  **n=50** | **EMM (SE)**  **n=50** | **ANCOVA** |
| *IEDSS Total Errors* | 18.21  (1.52) | 19.03 (1.54) | 11.85 (1.47) | 15.13 (1.45) | Group: F(3, 161)=4.79, **p=0.003**, ηp²=0.08 |
|  |  |  |  |  | Gender: F(1, 161)=0.10, p=0.75, ηp²=0.001 |
| *IEDSS Adjusted Errors* | 39.83  (5.21) | 32.68  (5.21) | 20.30 (5.21) | 27.16  (5.21) | Group: F(3, 195)=2.53, p=0.06, ηp²=0.04 |
|  |  |  |  |  | Gender: F(1, 195)=0.50, p=0.48, ηp²=0.003 |
| *IEDSS Pre-Extra-Dimensional Shift Errors* | 13.33  (1.41) | 12.88  (1.41) | 7.74  (1.41) | 10.40  (1.41) | Group: F(3, 195)=3.34, **p=0.02**, ηp²=0.05 |
|  |  |  |  |  | Gender: F(1, 195)=0.13, p=0.72, ηp²=0.001 |
| *IEDSS Total Trials Completed* | 85.81  (2.96) | 86.03 (3.00) | 72.55 (2.86) | 77.74 (2.82) | Group: F(3, 161)=5.11, **p=0.02**, ηp²=0.09 |
|  |  |  |  |  | Gender: F(1, 161)=0.02, p=0.89, ηp²=0.00 |
| *IEDSS Response Latency* | 131249.94 (8622.23) | 136477.51 (8737.38) | 91849.92 (8319.02) | 107038.42 (8220.03) | Group: F(3, 161)=6.08, **p<0.001**, ηp²=0.10 |
|  |  |  |  |  | Gender: F(1, 161)=0.40, p=0.53, ηp²=0.00 |
| ***WASI-II FSIQ-2*** | **EMM (SE)**  **n=40** | **EMM (SE)**  **n=39** | **EMM (SE)**  **n=43** | **EMM (SE)**  **n=44** | **ANCOVA** |
| *IEDSS Total Errors* | 17.25 (1.51) | 18.65 (1.50) | 12.75 (1.46) | 15.46 (1.41) | Group: F(3, 161)=2.85, **p=0.04**, ηp²=0.05 |
|  |  |  |  |  | WASI-II FSIQ-2: F(1, 161)=8.95, **p=0.003,** ηp²=0.05 |
| *IEDSS Adjusted Errors* | 35.43 (5.09) | 29.26 (5.06) | 25.19 (5.13) | 30.10 (5.03) | Group: F(3, 195)=0.66, p=0.58, ηp²=0.01 |
|  |  |  |  |  | WASI-II FSIQ-2: F(1, 195)=18.07, **p<0.001,** ηp²=0.009 |
| *IEDSS Pre-Extra-Dimensional Shift Errors* | 12.03  (1.36) | 11.84  (1.35) | 9.23  (1.37) | 11.26  (1.35) | Group: F(3, 195)=0.84, p=0.48, ηp²=0.01 |
|  |  |  |  |  | WASI-II FSIQ-2: F(1, 195)=22.48, **p<0.001,** ηp²=0.10 |
| *IEDSS Total Trials Completed* | 83.80 (2.93) | 85.21 (2.91) | 74.46 (2.83) | 78.43 (2.74) | Group: F(3, 161)=2.84, **p=0.04**, ηp²=0.05 |
|  |  |  |  |  | WASI-II FSIQ-2: F(1, 161)=10.60, **p=0.001,** ηp²=0.06 |
| *IEDSS Response Latency* | 124352.72 (8449.75) | 133857.17 (8401.10) | 98198.34 (8150.27) | 109427.06 (7900.89) | Group: F(3, 161)=3.48, **p=0.02**, ηp²=0.06 |
|  |  |  |  |  | WASI-II FSIQ-2: F(1, 161)=14.65, **p=0.001,** ηp²=0.08 |
| ***Psychotropic medication***  ***(FS/FMS/CC groups)*** | **EMM (SE)**  **n=40** | **EMM (SE)**  **n=39** | **EMM (SE)**  **n=43** |  | **ANCOVA** |
| *IEDSS Total Errors* | 18.31 (1.45) | 19.24  (1.50) | 11.56 (1.44) | - | Group: F(2, 118)=8.14, **p<0.001**, ηp²=0.12 |
|  |  |  |  |  | Psychotropics: F(3, 161)=2.19, p=0.14, ηp²=0.02 |
| *IEDSS Adjusted Errors* | 39.25 (5.09) | 32.21 (5.10) | 21.24 (5.15) | - | Group: F(2, 146)=3.09, **p=0.049**, ηp²=0.04 |
|  |  |  |  |  | Psychotropics: F(1, 146)=1.64, p=0.20, ηp²=0.01 |
| *IEDSS Pre-Extra-Dimensional Shift Errors* | 13.07  (1.43) | 12.62  (1.43) | 8.25  (1.44) | - | Group: F(2, 146)=3.36, **p=0.04**, ηp²=0.04 |
|  |  |  |  |  | Psychotropics: F(1, 146)=4.91, **p=0.03**, ηp²=0.03 |
| *IEDSS Total Trials Completed* | 85.95 (2.96) | 86.31 (3.01) | 72.16 (2.86) | - | Group: F(2, 118)=7.57, **p<0.001**, ηp²=0.11 |
|  |  |  |  |  | Psychotropics: F(1, 118)=1.05, p=0.31, ηp²=0.01 |
| *IEDSS Response Latency* | 130536.6  (9391.45) | 135718.33  (9533.25) | 93102.90  (9122.23) | - | Group: F(2, 118)=6.24, **p=0.003**, ηp²=0.10 |
|  |  |  |  |  | Psychotropics: F(1, 118)=1.51, p=0.22, ηp²=0.01 |
| ***MST Mean Motor Latency*** | **EMM (SE)**  **n=40** | **EMM (SE)**  **n=39** | **EMM (SE)**  **n=43** | **EMM (SE)**  **n=44** | **ANCOVA** |
| *IEDSS Total Errors* | 18.18 (1.52) | 18.77 (1.59) | 12.02 (1.48) | 15.23 (1.45) | Group: F(3, 161)=4.04, **p=0.008**, ηp²=0.07 |
|  |  |  |  |  | MST MML: F(1, 161)=0.50, 0.48 ηp²=0.00 |
| *IEDSS Adjusted Errors* | 38.47 (5.12) | 28.59 (5.28) | 23.23 (5.20) | 29.69 (5.16) | Group: F(3, 195)=1.50, p=0.22, ηp²=0.02 |
|  |  |  |  |  | MST MML: F(1, 195)=9.14, **p=0.003**, ηp²=0.05 |
| *IEDSS Pre-Extra-Dimensional Shift Errors* | 13.14  (1.41) | 12.31  (1.45) | 8.16  (1.43) | 10.76  (1.42) | Group: F(3, 195)=2.25, p=0.08, ηp²=0.03 |
|  |  |  |  |  | MST MML: F(1, 195)=2.41, p=0.12, ηp²=0.01 |
| *IEDSS Total Trials Completed* | 85.76 (2.95) | 85.37 (3.09) | 72.97 (2.89) | 77.97 (2.82) | Group: F(3, 161)=4.25, **p=0.006**, ηp²=0.07 |
|  |  |  |  |  | MST MML: F(1, 161)=0.78, p=0.38, ηp²=0.01 |
| *IEDSS Response Latency* | 130716.28 (8395.52) | 130064.99 (8779.96) | 95919.50 (8217.51) | 109230.32 (8033.64) | Group: F(3, 161)=3.95, **p=0.009**, ηp²=0.07 |
|  |  |  |  |  | MST MML: F(1, 161)=9.18, **p=0.003**, ηp²=0.05 |
| ***RTT Mean Reaction Time*** | **EMM (SE)**  **n=40** | **EMM (SE)**  **n=39** | **EMM (SE)**  **n=43** | **EMM (SE)**  **n=44** | **ANCOVA** |
| *IEDSS Total Errors* | 18.16 (1.53) | 18.89 (1.64) | 11.92 (1.49) | 15.24 (1.50) | Group: F(3, 161)=3.88, **p=0.01**, ηp²=0.07 |
|  |  |  |  |  | RTT MRT: F(1, 161)=0.08, p=0.79, ηp²=0.00 |
| *IEDSS Adjusted Errors* | 37.84 (5.08) | 26.21 (5.38) | 23.66 (5.15) | 32.27 (5.24) | Group: F(3, 195)=1.58, p=0.20, ηp²=0.02 |
|  |  |  |  |  | RTT MRT: F(1, 195)=12.71, **p<0.001**, ηp²=0.06 |
| *IEDSS Pre-Extra-Dimensional Shift Errors* | 12.87  (1.38) | 11.36  (1.47) | 8.55  (1.40) | 11.59 (1.43) | Group: F(3, 195)=1.70, p=0.17, ηp²=0.03 |
|  |  |  |  |  | RTT MRT: F(1, 195)=9.41, **p=0.002**, ηp²=0.05 |
| *IEDSS Total Trials Completed* | 85.79 (2.98) | 86.02 (3.21) | 72.55 (2.91) | 77.77 (2.91) | Group: F(3, 161)=4.32, **p=0.006**, ηp²=0.07 |
|  |  |  |  |  | RTT MRT: F(1, 161)=0.00, p=0.98, ηp²=0.00 |
| *IEDSS Response Latency* | 129471.81 (8597.81) | 130888.53 (9258.94) | 94647.13 (8414.34) | 110875.15 (8418.51) | Group: F(3, 161)=3.58, **p=0.02**, ηp²=0.06 |
|  |  |  |  |  | RTT MRT: F(1, 161)=3.14, p=0.08, ηp²=0.02 |
| ***RTT Mean Movement Time*** | **EMM (SE)**  **n=40** | **EMM (SE)**  **n=39** | **EMM (SE)**  **n=43** | **EMM (SE)**  **n=44** | **ANCOVA** |
| *IEDSS Total Errors* | 18.33 (1.56) | 19.11 (1.54) | 11.74 (1.49) | 15.07 (1.46) | Group: F(3, 161)=4.68, **p=0.004**, ηp²=0.08 |
|  |  |  |  |  | RTT MMT: F(1, 161)=0.13, p=0.72, ηp²=0.00 |
| *IEDSS Adjusted Errors* | 38.85 (5.30) | 32.06 (5.27) | 21.00 (5.29) | 28.07 (5.28) | Group: F(3, 195)=1.89, p=0.13, ηp²=0.03 |
|  |  |  |  |  | RTT MMT: F(1, 195)=0.83, p=0.36, ηp²=0.00 |
| *IEDSS Pre-Extra-Dimensional Shift Errors* | 12.87  (1.42) | 12.54  (1.42) | 8.14  (1.42) | 10.81  (1.42) | Group: F(3, 195)=2.19, p=0.09, ηp²=0.03 |
|  |  |  |  |  | RTT MMT: F(1, 195)=2.77, p=0.10, ηp²=0.01 |
| *IEDSS Total Trials Completed* | 86.02 (3.04) | 86.14 (3.01) | 72.37 (2.90) | 77.63 (2.85) | Group: F(3, 161)=4.88, **p=0.003**, ηp²=0.08 |
|  |  |  |  |  | RTT MMT: F(1, 161)=0.10, p=0.76, ηp²=0.00 |
| *IEDSS Response Latency* | 128311.70 (8823.99) | 135534.93 (8732.75) | 93762.02 (8418.07) | 108676.38 (8259.06) | Group: F(3, 161)=4.62, **p=0.004**, ηp²=0.08 |
|  |  |  |  |  | RTT MMT: F(1, 161)=1.86, p=0.17, ηp²=0.01 |

**Key:** ANCOVA=Analysis of Covariance; ANOVA=Analysis of Variance; CC=clinical control; EMM=estimated marginal means; FMS=functional motor symptoms; FS=functional seizures; HC=healthy control; IEDSS=Intra-Extra Dimensional Set Shift; M=mean; MST MML=Motor Screening Test Mean Motor Latency; MSVT=Medical Symptom Validity Test; RTT MMT=Reaction Time Test Mean Movement Time; RTT MRT=Reaction Time Test Mean Reaction Time; SD=standard deviation; SE=standard error; WASI-II FSIQ-2=Wechsler Abbreviated Scale of Intelligence – Second edition Full Scale Intelligence Quotient – 2 subtest, *Welch’s ANOVA

**Supplementary Table 15. Intra-Extra Dimensional Set Shift post-hoc tests**

| **Test outcome** | **Comparison** | **Mean difference (standard error)** | **Confidence interval (95%)** | **p-value** |  |
| --- | --- | --- | --- | --- | --- |
| ***IEDSS Total Errors^#^*** | FS vs FMS | -0.85 (2.53) | -7.48, 5.78 | 0.99 |  |
|  | FS vs HC | 3.06 (2.40) | -3.24, 9.36 | 0.58 |  |
|  | FS vs CC | 6.36 (1.98) | 1.10, 11.63 | **0.01** |  |
|  | FMS vs HC | 3.92 (2.28) | -2.06, 9.89 | 0.32 |  |
|  | FMS vs CC | 7.21 (1.83) | 2.35, 12.08 | **0.001** |  |
|  | HC vs CC | 3.30 (1.65) | -1.06, 7.66 | 0.20 |  |
| ***IEDSS Total Trials Completed^#^*** | FS vs FMS | -0.25 (4.98) | -13.33, 12.83 | 1.00 |  |
|  | | FS vs HC | 8.05 (4.70) | -4.32, 20.42 | 0.33 |
|  | | FS vs CC | 13.27 (4.06) | 2.47, 24.06 | **0.01** |
|  | | FMS vs HC | 8.30 (4.26) | -2.89, 19.49 | 0.22 |
|  | | FMS vs CC | 13.52 (3.54) | 4.13, 22.91 | **0.002** |
|  | | HC vs CC | 5.22 (3.14) | -3.07, 13.50 | 0.35 |
| ***IEDSS Response Latency^#^*** | | FS vs FMS | -5591.37 (15816.16) | -47247.62, 36064.89 | 0.99 |
|  | | FS vs HC | 23983.91 (10595.76) | -3943.68, 51911.49 | 0.12 |
|  | | FS vs CC | 39458.25 (10252.27) | 12374.75, 66541.74 | **0.002** |
|  | | FMS vs HC | 29575.27 (14155.49) | -8000.12, 67150.66 | 0.17 |
|  | | FMS vs CC | 45049.61 (13900.25) | 8074.83, 82024.40 | **0.01** |
|  | | HC vs CC | 15474.34 (7438.82) | -4025.15, 34973.83 | 0.17 |

**Key:** CC=clinical control; FMS=functional motor symptoms; FS=functional seizures; HC=healthy control; IEDSS=Intra-Extra Dimensional Set Shift, ^#^Games-Howell

**Supplementary Table 16. Spatial Span Test uncorrected results**

|  | **FS**  **(n=50)** | **FMS**  **(n=50)** | **CC**  **(n=50)** | **HC**  **(n=50)** | **ANOVA** **statistics** |
| --- | --- | --- | --- | --- | --- |
| *SSP Forward Span Length: M (SD)* | 6.18  (1.17) | 6.16  (1.39) | 6.74  (1.43) | 6.78  (1.45) | F(3, 196)=3.13, **p=0.03**, η²=0.05 |
| *SSP Forward Errors: M (SD)* | 16.46  (6.27) | 15.72  (9.84) | 14.0  (7.23) | 13.34  (5.31) | F(3, 196)=1.95, p=0.122, η²=0.03 |
| *SSP Reverse Span Length: M (SD)* | 5.62  (1.37) | 5.60  (1.21) | 6.60  (1.34) | 6.30  (1.42) | F(3, 196)=7.00, **p<0.001**, η²=0.10 |
| *SSP Reverse Errors: M (SD)* | 12.30  (5.02) | 13.06  (5.01) | 13.66  (5.84) | 12.20  (5.80) | F(3, 196)=0.80, p=0.50, η²=0.01 |

**Key:** CC=clinical controls; FS=functional seizures; FMS=functional motor symptoms; HC=healthy controls; M=mean; ms=milliseconds; SD=standard deviation

^*^Welch’s ANOVA

**Supplementary Table 17. Spatial Span Test sensitivity analyses**

| **Adjustment / Test outcome*^β^*** | **FS** | **FMS** | **CC** | **HC** | **Test statistics** |
| --- | --- | --- | --- | --- | --- |
| ***MSVT fails excluded*** | **M (SD)**  **n=48** | **M (SD)**  **n=46** | **M (SD)**  **n=48** | **M (SD)**  **n=49** | **ANOVA** |
| *SSP Forward Span Length* | 6.23 (1.17) | 6.33 (1.32) | 6.77 (1.39) | 6.82 (1.44) | F(3, 187)=2.45, p=0.07, η²=0.04 |
| *SSP Reverse Span Length* | 5.71 (1.32) | 5.70 (1.21) | 6.67 (1.26) | 6.33 (1.42) | F(3, 187)=6.41, **p<0.001**, η²=0.09 |
| ***Education*** | **EMM (SE)**  **n=50** | **EMM (SE)**  **n=50** | **EMM (SE)**  **n=50** | **EMM**  **(SE)**  **n=50** | **ANCOVA** |
| *SSP Forward Span Length* | 6.19 (0.19) | 6.18 (0.20) | 6.73 (0.19) | 6.76 (0.20) | Group: F(3, 195)=2.63, p=0.05, ηp²=0.04 |
|  |  |  |  |  | Education: F(1, 195)=0.29, p=0.59, ηp²=0.00 |
| *SSP Reverse Span Length* | 5.64 (0.19) | 5.63 (0.19) | 6.58 (0.19) | 6.27 (0.19) | Group: F(3, 195)=5.70, **p<0.001,** ηp²=0.08 |
|  |  |  |  |  | Education: F(1, 195)=0.82, p=0.37, ηp²=0.00 |
| ***Age*** | **EMM (SE)**  **n=50** | **EMM (SE)**  **n=50** | **EMM**  **(SE)**  **n=50** | **EMM**  **(SE)**  **n=50** | **ANCOVA** |
| *SSP Forward Span Length* | 6.17 (0.18) | 6.12 (0.18) | 6.76 (0.18) | 6.81 (0.18) | Group: F(3, 195)=4.27, **p=0.006**, ηp²=0.06 |
|  |  |  |  |  | Age: F(1, 195)=33.02, **p<0.001**, ηp²=0.15 |
| *SSP Reverse Span Length* | 5.62 (0.19) | 5.58 (0.19) | 6.61 (0.19) | 6.32 (0.19) | Group: F(3, 195)=7.77, **p<0.001**, ηp²=0.11 |
|  |  |  |  |  | Age: F(1, 195)=10.33, **p=0.002**, ηp²=0.05 |
| ***Gender*** | **EMM (SE)**  **n=50** | **EMM (SE)**  **n=50** | **EMM**  **(SE)**  **n=50** | **EMM**  **(SE)**  **n=50** | **ANCOVA** |
| *SSP Forward Span Length* | 6.17 (0.19) | 6.17 (0.19) | 6.72 (0.19) | 6.79 (0.19) | Group: F(3, 195)=3.15, **p=0.03**, ηp²=0.05 |
|  |  |  |  |  | Gender: F(1, 195)=5.04, **p=0.03**, ηp²=0.03 |
| *SSP Reverse Span Length* | 5.62 (0.19) | 5.61 (0.19) | 6.59 (0.19) | 6.31 (0.19) | Group: F(3, 195)=6.92, **p<0.001**, ηp²=0.10 |
|  |  |  |  |  | Gender: F(1, 195)=1.77, p=0.19, ηp²=0.009 |
| ***WASI-II FSIQ-2*** | **EMM (SE)**  **n=50** | **EMM (SE)**  **n=50** | **EMM**  **(SE)**  **n=50** | **EMM**  **(SE)**  **n=50** | **ANCOVA** |
| *SSP Forward Span Length* | 6.38  (0.18) | 6.32  (0.18) | 6.51 (0.19) | 6.65  (0.18) | Group: F(3, 195)=0.61, p=0.61 ηp²=0.009 |
|  |  |  |  |  | WASI-II FSIQ-2: F(1, 195)=28.75, **p<0.001,** ηp²=0.13 |
| *SSP Reverse Span Length* | 5.82  (0.18) | 5.76 (0.18) | 6.37 (0.18) | 6.17  (0.18) | Group: F(3, 195)=2.36, p=0.07 ηp²=0.04 |
|  |  |  |  |  | WASI-II FSIQ-2: F(1, 195)=30.36, **p<0.001,** ηp²=0.14 |
| ***Psychotropic medication***  ***(FS/FMS/CC groups)*** | **EMM (SE)**  **n=50** | **EMM (SE)**  **n=50** | **EMM (SE)**  **n=50** |  | **ANCOVA** |
| *SSP Forward Span Length* | 6.20  (0.19) | 6.18  (0.19) | 6.70 (0.19) | - | Group: F(2, 146)=2.33, p=0.10 ηp²=0.03 |
|  |  |  |  |  | Psychotropics: F(1, 146)=1.70, p=0.19, ηp²=0.01 |
| *SSP Reverse Span Length* | 5.64 (0.19) | 5.62 (0.19) | 6.57 (0.19) | - | Group: F(2, 146)=8.25, **p<0.001**, ηp²=0.10 |
|  |  |  |  |  | Psychotropics: F(1, 146)=1.16, p=0.28, ηp²=0.01 |
| ***MST Mean Motor Latency*** | **EMM (SE)**  **n=50** | **EMM (SE)**  **n=50** | **EMM (SE)**  **n=50** | **EMM**  **(SE)**  **n=50** | **ANCOVA** |
| *SSP Forward Span Length* | 6.22 (0.19) | 6.29 (0.20) | 6.64 (0.19) | 6.71 (0.19) | Group: F(3, 195)=1.50, p=0.22 ηp²=0.02 |
|  |  |  |  |  | MST MML: F(1, 195)=6.31, **p=0.01**, ηp²=0.03 |
| *SSP Reverse Span Length* | 5.65 (0.19) | 5.71 (0.19) | 6.52 (0.19) | 6.24 (0.19) | Group: F(3, 195)=4.46, **p=0.005**, ηp²=0.06 |
|  |  |  |  |  | MST MML: F(1, 195)=4.80, **p=0.03**, ηp²=0.02 |
| ***RTT Mean Reaction Time*** | **EMM (SE)**  **n=50** | **EMM (SE)**  **n=50** | **EMM (SE)**  **n=50** | **EMM**  **(SE)**  **n=50** |  |
| *SSP Forward Span Length* | 6.26 (0.19) | 6.43 (0.19) | 6.59 (0.19) | 6.57 (0.19) | Group: F(3, 195)=0.63, p=0.60, ηp²=0.01 |
|  |  |  |  |  | RTT MRT: F(1, 195)=16.30, **p<0.001**, ηp²=0.08 |
| *SSP Reverse Span Length* | 5.70 (0.18) | 5.87 (0.19) | 6.46 (0.19) | 6.10 (0.19) | Group: F(3, 195)=2.92, **p=0.04**, ηp²=0.04 |
|  |  |  |  |  | RTT MRT: F(1, 195)=16.56, **p<0.001**, ηp²=0.08 |
| ***RTT Mean Movement Time*** | **EMM (SE)**  **n=50** | **EMM (SE)**  **n=50** | **EMM (SE)**  **n=50** | **EMM**  **(SE)**  **n=50** |  |
| *SSP Forward Span Length* | 6.27 (0.19) | 6.23 (0.19) | 6.66 (0.19) | 6.71 (0.19) | Group: F(3, 195)=1.55, p=0.20, ηp²=0.02 |
|  |  |  |  |  | RTT MMT: F(1, 195)=5.65, **p=0.02**, ηp²=0.03 |
| *SSP Reverse Span Length* | 5.70 (0.19) | 5.66 (0.19) | 6.53 (0.19) | 6.24 (0.19) | Group: F(3, 195)=4.60, **p=0.004**, ηp²=0.07 |
|  |  |  |  |  | RTT MMT: F(1, 195)=4.34, **p=0.04**, ηp²=0.02 |

**Key:** ANCOVA=Analysis of Covariance; ANOVA=Analysis of Variance; CC=clinical control; EMM=estimated marginal means; FMS=functional motor symptoms; FS=functional seizures; HC=healthy control; M=mean; MMT=Mean Movement Time; MST MML=Motor Screening Test Mean Motor Latency; MRT=Mean Reaction Time; MSVT=Medical Symptom Validity Test; RTT=Reaction Time Test; SD=standard deviation; SE=standard error; SSP=Spatial Span Test; WASI-II FSIQ-2=Wechsler Abbreviated Scale of Intelligence – Second edition Full Scale Intelligence Quotient – 2 subtest

^β^No outliers present

**Supplementary Table 18. Stop Signal Task uncorrected results**

|  | **FS**  **(n=49)** | **FMS**  **(n=48)** | **CC**  **(n=50)** | **HC**  **(n=50)** | **ANOVA** **statistics** |
| --- | --- | --- | --- | --- | --- |
| *Errors Go Trials: M (SD)* | 2.71  (4.35) | 2.94  (4.22) | 1.56  (2.55) | 1.64  (2.20) | *F(3,102.91)=2.04, p=0.113, ω²=0.02 |
| *Errors Stop Trials: M (SD)* | 40.69  (4.67) | 42.27  (4.00) | 40.86 (4.00) | 41.36 (5.50) | F(3, 193)=1.03, p=0.38, ω²=0.02 |
| *Number of Missed Trials: M (SD)* | 6.67  (6.08) | 6.19  (5.95) | 3.88  (3.81) | 5.46 (11.28) | F(3,193)=1.37, **p=0.03**, η²=0.02 |
| *Stop Signal Reaction Time (ms):*  *M (SD)* | 244.02 (56.98) | 253.91 (58.47) | 229.42  (47.93) | 243.24 (59.85) | F(3, 193)=1.59, p=0.19, η²=0.02 |

**Key:** CC=clinical controls; FS=functional seizures; FMS=functional motor symptoms; HC=healthy controls; M=mean; ms=milliseconds; SD=standard deviation

^*^Welch’s ANOVA

**Supplementary Table 19. Stop Signal Task sensitivity analyses**

| **Adjustment / Test outcome** | **FS** | **FMS** | **CC** | **HC** | **Test statistics** |
| --- | --- | --- | --- | --- | --- |
| ***MSVT fails excluded*** | **M (SD)**  **n=48** | **M (SD)**  **n=45** | **M (SD)**  **n=48** | **M (SD)**  **n=49** | **ANOVA** |
| *SST Number of Missed Trials* | 6.479 (5.99) | 5.96  (5.81) | 3.79  (3.72) | 5.51 (11.39) | F(3, 186)=1.20, p=0.31, ω²=0.02 |
| ***Outliers winsorised*** | **M (SD)**  **n=48** | **M (SD)**  **n=45** | **M (SD)**  **n=48** | **M (SD)**  **n=49** | **ANOVA** |
| *SST Number of Missed Trials* | 6.31  (5.49) | 5.78  (5.28) | 3.63  (3.32) | 4.41  (5.12) | *F(3, 100.25)=3.66, **p=0.02**, ω²=0.03 |
| ***Education*** | **EMM (SE)**  **n=49** | **EMM (SE)**  **n=48** | **EMM (SE)**  **n=50** | **EMM (SE)**  **n=50** | **ANCOVA** |
| *SST Number of Missed Trials* | 6.65  (1.05) | 6.14  (1.08) | 3.91  (1.05) | 5.51  (1.05) | Group: F(3, 192)=1.26, p=0.29, ηp²=0.02 |
|  |  |  |  |  | Education: F(1, 192)=0.07, p=0.79, ηp²=0.00 |
| ***Age*** | **EMM (SE)**  **n=49** | **EMM (SE)**  **n=48** | **EMM (SE)**  **n=50** | **EMM (SE)**  **n=50** | **ANCOVA** |
| *SST Number of Missed Trials* | 6.68  (1.05) | 6.22  (1.06) | 3.87  (1.04) | 5.44  (1.04) | Group: F(3, 192)=1.40, p=0.24, ηp²=0.02 |
|  |  |  |  |  | Age: F(1, 192)=0.51, p=0.48, ηp²=0.003 |
| ***Gender*** | **EMM (SE)**  **n=49** | **EMM (SE)**  **n=48** | **EMM (SE)**  **n=50** | **EMM (SE)**  **n=50** | **ANCOVA** |
| *SST Number of Missed Trials* | 6.73  (1.05) | 6.13  (1.06) | 3.94  (1.04) | 5.41  (1.04) | Group: F(3, 192)=1.34, p=0.26, ηp²=0.02 |
|  |  |  |  |  | Gender: F(1, 192)=1.73, p=0.19, ηp²=0.009 |
| ***WASI-II FSIQ-2*** | **EMM (SE)**  **n=49** | **EMM (SE)**  **n=48** | **EMM (SE)**  **n=50** | **EMM (SE)**  **n=50** | **ANCOVA** |
| *SST Number of Missed Trials* | 6.59  (1.07) | 6.12  (1.08) | 3.98  (1.07) | 5.51  (1.05) | Group: F(3, 192)=1.07, p=0.37, ηp²=0.02 |
|  |  |  |  |  | WASI-II FSIQ-2: F(1, 192)=0.16, p=0.69, ηp²=0.00 |
| ***Psychotropic medication***  ***(FS/FMS/CC groups)*** | **EMM (SE)**  **n=49** | **EMM (SE)**  **n=48** | **EMM (SE)**  **n=50** |  | **ANCOVA** |
| *SST Number of Missed Trials* | 6.61 (0.77) | 6.10  (0.78) | 4.03  (0.77) | - | Group: F(2, 143)=3.09, **p=0.049**, ηp²=0.04 |
|  |  |  |  |  | Psychotropics: F(1, 143)=1.35, p=0.25, ηp²=0.01 |
| ***MST Mean Motor Latency*** | **EMM (SE)**  **n=49** | **EMM (SE)**  **n=48** | **EMM (SE)**  **n=50** | **EMM (SE)**  **n=50** | **ANCOVA** |
| *SST Number of Missed Trials* | 6.54  (1.04) | 5.63  (1.10) | 4.26  (1.05) | 5.75  (1.04) | Group: F(3, 192)=0.81, p=0.49, ηp²=0.01 |
|  |  |  |  |  | MST MML: F(1, 192)=3.43, p=0.07, ηp²=0.02 |
| ***RTT Mean Reaction Time*** | **EMM (SE)**  **n=49** | **EMM (SE)**  **n=48** | **EMM (SE)**  **n=50** | **EMM (SE)**  **n=50** | **ANCOVA** |
| *SST Number of Missed Trials* | 6.39  (1.03) | 4.99  (1.11) | 4.47  (1.03) | 6.31  (1.05) | Group: F(3, 192)=0.88, p=0.45, ηp²=0.01 |
|  |  |  |  |  | RTT MRT: F(1, 192)=9.41, **p=0.002**, ηp²=0.05 |
| ***RTT Mean Movement Time*** | **EMM (SE)**  **n=49** | **EMM (SE)**  **n=48** | **EMM (SE)**  **n=50** | **EMM (SE)**  **n=50** | **ANCOVA** |
| *SST Number of Missed Trials* | 6.62  (1.07) | 6.14  (1.07) | 3.93  (1.06) | 5.51  (1.05) | Group: F(3, 192)=1.16, p=0.33, ηp²=0.02 |
|  |  |  |  |  | RTT MMT: F(1, 192)=0.08, p=0.78, ηp²=0.00 |

**Key:** ANCOVA=Analysis of Covariance; ANOVA=Analysis of Variance; CC=clinical control; EMM=estimated marginal means; FMS=functional motor symptoms; FS=functional seizures; HC=healthy control; M=mean; SD=standard deviation; SE=standard error; WASI-II FSIQ-2=Wechsler Abbreviated Scale of Intelligence – Second edition Full Scale Intelligence Quotient – 2 subtest

*Welch’s ANOVA

**Supplementary Table 20. Emotional bias and recognition tasks uncorrected results**

|  | **FS** | **FMS** | **CC** | **HC** | **ANOVA statistics** |
| --- | --- | --- | --- | --- | --- |
| ***Emotional Bias Task - Anger (EBT-A)*** | **n=49** | **n=50** | **n=50** | **n=49** | ^$^Group: F(3, 194)=3.71, **p=0.01**, ηp²=0.05  Emotion: F(1,194)= 19.44, **p<0.001**, ηp²=0.09  Group x Emotion: F(3, 194)=2.67, **p=0.049**, ηp²=0.04 |
| *EBT-A Mdn RT Happiness (ms): M (SD)* | 772.14 (192.77) | 820.50 (264.27) | 748.13 (193.35) | 736.91 (225.02) |  |
| *EBT-A Mdn RT Anger (ms): M (SD)* | 834.76 (248.82) | 1040.71 (650.92) | 799.76 (193.00) | 824.83 (291.62) |  |
| *EBT-A Bias Point: M (SD)* | 8.63 (1.32) | 8.83 (1.57) | 8.55 (1.35) | 8.83 (1.50) | F(3, 195)=4.90, p=0.69, η²=0.01 |
| ***Emotional Bias Task – Disgust (EBT-D)*** | **n=50** | **n=49** | **n=50** | **n=50** | ^$^Group: F(3, 195)=3.11, **p=0.03**, ηp²=0.05  Emotion: F(1,195)=14.88, **p<0.001**, ηp²=0.07  Group x Emotion: F(3, 195)=0.26, p=0.86, ηp²=0.00 |
| *EBT-D Mdn RT Happiness (ms): M (SD)* | 768.83 (191.26) | 803.79  (310.71) | 666.41 (159.42) | 709.50 (287.09) |  |
| *EBT-D Mdn RT Disgust: M (SD)* | 804.89 (223.40) | 845.41 (334.04) | 714.61 (190.48) | 734.51 (269.46) |  |
| *EBT-D Bias Point: M (SD)* | 8.13 (1.55) | 8.21 (1.86) | 8.31 (1.23) | 8.41 (1.58) | F(3, 196)=0.31, p=0.82, η²=0.01 |
| ***Emotional Bias Task – Sadness (EBT-S)*** | **(n=49)** | **(n=50)** | **(n=50)** | **(n=50)** | ^$^Group: F(3, 195)=3.43, **p=0.02**, ηp²=0.05  Emotion: F(1,195)=2.82, p=0.10, ηp²=0.01  Group x Emotion: F(3, 195)=1.15, p=0.33, ηp²=0.02 |
| *EBT-S Mdn RT Happiness (ms): M (SD)* | 776.86 (214.30) | 818.53 (310.22) | 672.83 (167.44) | 710.37 (249.37) |  |
| *EBT-S Mdn RT Sadness (ms): M (SD)* | 754.19 (240.24) | 794.35 (272.98) | 649.77 (161.57) | 723.35 (308.12) |  |
| *EBT-S Bias Point: M (SD)* | 5.97 (1.29) | 6.33 (1.64) | 6.11 (1.31) | 5.84 (1.61) | F(3, 195)=1.01, p=0.39, η²=0.02 |
| ***Emotion Recognition Test (ERT) – Total Hits*** | **n=49** | **n=50** | **n=50** | **n=50** |  |
| *ERT Total Hits: M (SD)* | 55.96 (10.44) | 56.46 (11.10) | 61.96 (7.00) | 59.12 (8.44) | ^$^Group: F(3, 195)=4.32, **p=0.006**, ηp²=0.06  Emotion: F(4.45, 867.30)=182.88, **p<0.001**, ηp²=0.48  Group x Emotion: F(13.34, 867.30)=0.97, p=0.48, ηp²=0.02 |
| *ERT Total Hits Happiness: M (SD)* | 11.29 (2.62) | 11.88 (2.30) | 12.60 (1.49) | 12.08 (2.16) |  |
| *ERT Total Hits Anger:*  *M (SD)* | 7.80  (2.48) | 7.34  (2.94) | 8.02  (2.00) | 7.82  (2.48) |  |
| *ERT Total Hits Disgust: M (SD)* | 10.00 (3.12) | 10.08 (3.05) | 11.42 (2.54) | 10.76 (2.41) |  |
| *ERT Total Hits Fear:*  *M (SD)* | 5.82  (3.57) | 6.34 (3.15) | 7.52  (3.10) | 6.24  (3.01) |  |
| *ERT Total Hits Sadness: M (SD)* | 10.39 (2.46) | 9.76  (2.92) | 10.76 (2.49) | 10.72 (2.69) |  |
| *ERT Total Hits Surprise: M (SD)* | 10.67 (1.83) | 11.06 (2.13) | 11.64 (1.75) | 11.50 (1.67) |  |
| ***ERT Unbiased Hits*** | **n=48** | **n=48** | **n=49** | **n=50** |  |
| *ERT Unbiased Hits Happiness: M (SD)* | 0.59  (0.17) | 0.61  (0.18) | 0.67  (0.13) | 0.65  (0.16) | ^$^Group: F(3, 191)=4.45, **p=0.005**, ηp²=0.07  ^#^Emotion: F(4.35, 831.35)=183.59, **p<0.001**, ηp²=0.49  ^#^Group x Emotion: F(13.10, 831.35)=1.51, p=0.11, ηp²=0.02 |
| *ERT Unbiased Hits Anger: M (SD)* | 0.39  (0.15) | 0.39  (0.19) | 0.46  (0.14) | 0.44  (0.15) |  |
| *ERT Unbiased Hits Disgust: M (SD)* | 0.41  (0.19) | 0.41  (0.18) | 0.50  (0.13) | 0.49  (0.17) |  |
| *ERT Unbiased Hits Fear:*  *M (SD)* | 0.24  (0.19) | 0.28  (0.17) | 0.36  (0.19) | 0.25  (0.15) |  |
| *ERT Unbiased Hits Sadness: M (SD)* | 0.48  (0.16) | 0.48  (0.18) | 0.53  (0.12) | 0.50  (0.13) |  |
| *ERT Unbiased Hits Surprise: M (SD)* | 0.45  (0.15) | 0.46  (0.15) | 0.47  (0.13) | 0.47  (0.12) |  |
| ***ERT False Alarms*** | **n=49** | **n=50** | **n=50** | **n=50** |  |
| *ERT False Alarms Happiness: M (SD)* | 3.90 (3.99) | 4.76 (5.91) | 4.18 (4.59) | 3.84 (4.37) | ^$^Group: F(3, 191)=4.45, **p=0.005**, ηp²=0.07  ^#^Emotion: F(4.36, 849.92)=35.32, **p<0.001**, ηp²=0.15  ^#^Group x Emotion: F(13.08, 849.92)=1.12, p=0.34, ηp²=0.02 |
| *ERT False Alarms Anger: M (SD)* | 3.35 (4.13) | 2.84 (2.79) | 1.86 (2.26) | 1.78 (1.65) |  |
| *ERT False Alarms Disgust: M (SD)* | 7.65 (4.97) | 8.08 (4.44) | 6.98 (4.51) | 6.10 (4.60) |  |
| *ERT False Alarms Fear:*  *M (SD)* | 5.92 (5.37) | 5.44 (4.19) | 4.06 (3.29) | 5.90 (4.74) |  |
| *ERT False Alarms Sadness: M (SD)* | 5.82 (4.72) | 4.28 (3.52) | 4.74 (4.14) | 5.08 (3.86) |  |
| *ERT False Alarms Surprise: M (SD)* | 7.41 (5.11) | 8.14 (5.42) | 6.22 (4.93) | 8.18 (5.12) |  |
| ***ERT Median Reaction Time (Mdn RT)*** | **n=49** | **n=50** | **n=50** | **n=50** |  |
| *ERT Mdn RT – Correct Responses: M (SD)* | 1135.34 (330.27) | 1182.09 (354.35) | 979.38 (200.69) | 1019.34 (246.86) | ^$^Group: F(3, 185)=5.28, **p=0.002**, ηp²=0.08  ^#^Emotion: F(4.18, 773.49)=53.00, **p<0.001**, ηp²=0.22  ^#^Group x Emotion: F(12.54, 773.49)=1.33, p=0.19, ηp²=0.02 |
| *ERT Mdn RT Happiness: M (SD)* | 1047.26 (711.52) | 921.72 (309.96) | 836.38 (233.45) | 871.94 (298.73) |  |
| *ERT Mdn RT Anger: M (SD)* | 1437.89  (683.55) | 1281.80 (513.88) | 1053.21 (334.49) | 1227.90 (541.21) |  |
| *ERT Mdn RT Disgust: M (SD)* | 1331.54 (517.28 | 1415.09 (542.45) | 1072.76  (400.14) | 1227.21 (387.10) |  |
| *ERT Mdn RT Fear: M (SD)* | 1498.70 (599.27) | 1655.31 (707.94) | 1378.80 (496.74) | 1407.04 (567.96) |  |
| *ERT Mdn RT Sadness: M (SD)* | 1217.47 (359.91) | 1316.82 (531.52) | 1045.68 (278.17) | 1114.94 (448.74) |  |
| *ERT Mdn RT Surprise: M (SD)* | 1044.22 (420.19) | 1057.03 (399.12) | 929.40 (277.94) | 942.79 (435.53) |  |

**Key:** CC=clinical controls; EBT=Emotional Bias Task; ERT=Emotion Recognition Test; FS=functional seizures; FMS=functional motor symptoms; HC=healthy controls; M=mean; ms=milliseconds; RT=reaction time; SD=standard deviation

^$^Mixed ANOVA; ^#^Greenhouse=Geiser corrections applied (sphericity assumption violated)

**Supplementary Table 21. Emotional Bias Task sensitivity analyses**

| **Adjustment / Test outcome** | **FS** | **FMS** | **CC** | **HC** | **Test statistics** |
| --- | --- | --- | --- | --- | --- |
| ***MSVT fails excluded*** | **M (SD)** | **M (SD)** | **M (SD)** | **M (SD)** | **ANOVA** |
| *EBT-Anger Median Reaction Times*  (FS=47; FMS=46; CC=48; HC=49) | 781.29  (38.68) | 931.65  (39.09) | 776.66  (38.27) | 780.87  (37.88) | Group: F(3, 186)=3.23, **p=0.01**, ηp²=0.06  Emotion: F(1, 186)=17.46, **p<0.001**, ηp²=0.09  Group x Emotion: F(3, 186)=2.63, p=0.05, ηp²=0.04 |
| *EBT-Disgust Median Reaction Times*  (FS=48; FMS=45; CC=48; HC=49) | 784.26  (34.88) | 816.38  (36.03) | 692.19  (34.88) | 725.36  (34.53) | Group: F(3, 186)=2.53, p=0.06, ηp²=0.04  Emotion: F(1, 186)=13.88, **p<0.001**, ηp²=0.07  Group x Emotion: F(3, 186)=0.23, p=0.88, ηp²=0.00 |
| *EBT-Sadness Median Reaction Times*  (FS=47; FMS=46; CC=48; HC=49) | 759.94  (34.15) | 792.37  (34.52) | 662.25  (33.79) | 717.52  (33.44) | Group: F(3, 186)=2.73, **p=0.046**, ηp²=0.04  Emotion: F(1, 186)=4.39, **p=0.04**, ηp²=0.02  Group x Emotion: F(3, 186)=1.02, p=0.39, ηp²=0.02 |
| ***Outliers winsorised*** | **M (SD)** | **M (SD)** | **M (SD)** | **M (SD)** | **ANOVA** |
| *EBT-Anger Median Reaction Times*  (FS=47; FMS=46; CC=48; HC=49) | 772.89  (28.33) | 877.45  (28.64) | 770.79  (28.04) | 763.10  (27.75) | Group: F(3, 186)=3.65, **p=0.01**, ηp²=0.06  Emotion: F(1, 186)=36.60, **p<0.001**, ηp²=0.16  Group x Emotion: F(3, 186)=4.86, **p=0.003**, ηp²=0.07 |
| *EBT-Disgust Median Reaction Times*  (FS=48; FMS=45; CC=48; HC=49) | 777.46  (25.36) | 771.47  (26.19) | 689.63  (25.36) | 706.03  (25.10) | Group: F(3, 186)=3.09, **p=0.03**, ηp²=0.05  Emotion: F(1, 186)=18.38, **p<0.001**, ηp²=0.09  Group x Emotion: F(3, 186)=0.54, p=0.64, ηp²=0.01 |
| *EBT-Sadness Median Reaction Times*  (FS=47; FMS=46; CC=48; HC=49) | 756.81  (25.58) | 766.10  (25.86) | 655.82  (25.31) | 686.09  (25.05) | Group: F(3, 186)=4.46, **p=0.005**, ηp²=0.07  Emotion: F(1, 186)=5.46, **p=0.02**, ηp²=0.03  Group x Emotion: F(3, 186)=0.44, p=0.73, ηp²=0.01 |
| ***Education*** | **EMM (SE)** | **EMM (SE)** | **EMM (SE)** | **EMM (SE)** | **ANCOVA** |
| *EBT-Anger Median Reaction Times*  (FS=49; FMS=50; CC=50; HC=49) | 795.11  (38.29) | 916.49  (38.28) | 782.55  (37.92) | 794.83  (38.64) | Group: F(3, 193)=2.68, **p=0.048**, ηp²=0.04 |
|  |  |  |  |  | Emotion: F(1, 193)=5.54, **p=0.02**, ηp²=0.03 |
|  |  |  |  |  | Group x emotion: F(3, 193)=2.29, p=0.08, ηp²=0.03 |
|  |  |  |  |  | Education: F(1, 193)=4.61, **p=0.03**, ηp²=0.02 |
| *EBT-Disgust Median Reaction Times*  (FS=50; FMS=49; CC=50; HC=50) | 782.87  (34.44) | 817.17  (35.15) | 694.77  (34.46) | 729.01  (34.75) | Group: F(3, 194)=2.37, p=0.07, ηp²=0.04 |
|  |  |  |  |  | Emotion: F(1, 194)=3.73, p=0.06, ηp²=0.02 |
|  |  |  |  |  | Group x emotion: F(3, 194)=0.23, p=0.88, ηp²=0.00 |
|  |  |  |  |  | Education: F(1, 194)=1.54, p=0.22, ηp²=0.01 |
| *EBT-Sadness Median Reaction Times*  (FS=49; FMS=50; CC=48; HC=50) | 759.58  (34.17) | 796.40  (34.17) | 667.38  (33.84) | 726.66  (34.14) | Group: F(3, 194)=2.51, p=0.06, ηp²=0.04 |
|  |  |  |  |  | Emotion: F(1, 194)=2.23, p=0.14, ηp²=0.01 |
|  |  |  |  |  | Group x emotion: F(3, 194)=0.90, p=0.44, ηp²=0.01 |
|  |  |  |  |  | Education: F(1, 194)=2.91, p=0.09, ηp²=0.02 |
| ***Age*** | **EMM (SE)** | **EMM (SE)** | **EMM (SE)** | **EMM (SE)** | **ANCOVA** |
| *EBT-Anger Median Reaction Times*  (FS=49; FMS=50; CC=50; HC=49) | 805.17 (38.13) | 933.48 (37.77) | 772.38 (37.75) | 777.81 (38.15) | Group: F(3, 193)=3.99, **p=0.009**, ηp²=0.06 |
|  |  |  |  |  | Emotion: F(1, 193)=1.03, p=0.31, ηp²=0.05 |
|  |  |  |  |  | Group x emotion: F(3, 193)=2.69, **p=0.048**, ηp²=0.04 |
|  |  |  |  |  | Age: F(1, 193)=4.23, **p=0.04**, ηp²=0.02 |
| *EBT-Disgust Median Reaction Times*  (FS=50; FMS=49; CC=50; HC=50) | 787.43 (33.91) | 828.24 (34.28) | 688.92 (33.91) | 719.46 (33.92) | Group: F(3, 194)=3.45, **p=0.02**, ηp²=0.05 |
|  |  |  |  |  | Emotion: F(1, 194)=0.03, p=0.88, ηp²=0.00 |
|  |  |  |  |  | Group x emotion: F(3, 194)=0.28, p=0.84, ηp²=0.004 |
|  |  |  |  |  | Age: F(1, 194)=5.98, **p=0.02**, ηp²=0.03 |
| *EBT-Sadness Median Reaction Times*  (FS=49; FMS=50; CC=50; HC=50) | 766.89 (33.91) | 808.80 (33.58) | 659.84 (33.57) | 714.62 (33.58) | Group: F(3, 194)=3.69, **p=0.01**, ηp²=0.05 |
|  |  |  |  |  | Emotion: F(1, 194)=0.57, p=0.45, ηp²=0.003 |
|  |  |  |  |  | Group x emotion: F(3, 194)=1.14, p=0.34, ηp²=0.02 |
|  |  |  |  |  | Age: F(1, 194)=3.99, **p=0.047**, ηp²=0.02 |
| ***Gender*** | **EMM (SE)** | **EMM (SE)** | **EMM (SE)** | **EMM (SE)** | **ANCOVA** |
| *EBT-Anger Median Reaction Times*  (FS=49; FMS=50; CC=50; HC=49) | 804.07 (38.32) | 928.58 (37.96) | 776.19 (37.97) | 780.03 (38.33) | Group: F(3, 193)=3.58, **p=0.02**, ηp²=0.05 |
|  |  |  |  |  | Emotion: F(1, 193)=14.84, **p<0.001**, ηp²=0.07 |
|  |  |  |  |  | Group x emotion: F(3, 193)=2.52, p=0.06, ηp²=0.04 |
|  |  |  |  |  | Gender: F(1, 193)=2.21, p=0.14, ηp²=0.01 |
| *EBT-Disgust Median Reaction Times*  (FS=50; FMS=49; CC=50; HC=50) | 787.04 (34.42) | 824.39 (34.77) | 691.03 (34.44) | 721.51 (34.44) | Group: F(3, 194)=3.08, **p=0.03**, ηp²=0.05 |
|  |  |  |  |  | Emotion: F(1, 194)=2.35, p=0.13, ηp²=0.01 |
|  |  |  |  |  | Group x emotion: F(3, 194)=0.25, p=0.86, ηp²=0.004 |
|  |  |  |  |  | Gender: F(1, 194)=0.15, p=0.70, ηp²=0.001 |
| *EBT-Sadness Median Reaction Times*  (FS=49; FMS=50; CC=50; HC=50) | 765.71 (34.24) | 806.09 (33.91) | 661.82 (33.93) | 716.51 (33.91) | Group: F(3, 194)=3.38, **p=0.02**, ηp²=0.05 |
|  |  |  |  |  | Emotion: F(1, 194)=0.06, p=0.81, ηp²=0.00 |
|  |  |  |  |  | Group x emotion: F(3, 194)=1.11, p=0.35, ηp²=0.02 |
|  |  |  |  |  | Gender: F(1, 194)=0.12, p=0.73, ηp²=0.001 |
| ***WASI-II FSIQ-2*** | **EMM (SE)** | **EMM (SE)** | **EMM (SE)** | **EMM (SE)** | **ANCOVA** |
| *EBT-Anger Median Reaction Times*  (FS=49; FMS=50; CC=50; HC=49) | 782.11 (38.66) | 912.82 (38.04) | 797.59 (38.48) | 796.23 (38.27) | Group: F(3, 193)=1.07, p=0.37, ηp²=0.02 |
|  |  |  |  |  | Emotion: F(1, 193)=1.04, p=0.31, ηp²=0.01 |
|  |  |  |  |  | Group x emotion: F(3, 193)=2.42, p=0.07, ηp²=0.04 |
|  |  |  |  |  | WASI-II FSIQ-2: F(1, 193)=7.23, **p=0.008**, ηp²=0.04 |
| *EBT-Disgust Median Reaction Times*  (FS=50; FMS=49; CC=50; HC=50) | 772.46 (34.82) | 813.89 (34.83) | 706.59 (35.00) | 730.82 (34.36) | Group: F(3, 194)=1.72, p=0.17, ηp²=0.03 |
|  |  |  |  |  | Emotion: F(1, 194)=0.82, p=0.37, ηp²=0.00 |
|  |  |  |  |  | Group x emotion: F(3, 194)=0.31, p=0.82, ηp²=0.01 |
|  |  |  |  |  | WASI-II FSIQ-2: F(1, 194)=4.04, p=0.05, ηp²=0.02 |
| *EBT-Sadness Median Reaction Times*  (FS=49; FMS=50; CC=50; HC=50) | 747.87 (34.34) | 791.80 (33.80) | 681.85 (34.25) | 728.25 (33.61) | Group: F(3, 194)=1.70, p=0.17, ηp²=0.03 |
|  |  |  |  |  | Emotion: F(1, 194)=0.35, p=0.56, ηp²=0.00 |
|  |  |  |  |  | Group x emotion: F(3, 194)=1.29, p=0.28, ηp²=0.02 |
|  |  |  |  |  | WASI-II FSIQ-2: F(1, 194)=6.78, **p=0.01**, ηp²=0.03 |
| ***Psychotropic medication (FS/FMS/CC)*** | **EMM (SE)** | **EMM (SE)** | **EMM (SE)** |  | **ANCOVA** |
| *EBT-Anger Median Reaction Times*  (FS=49; FMS=50; CC=50) | 797.17  (39.00) | 921.61  (38.67) | 789.10  (39.00) | - | Group: F(2, 145)=3.66, **p=0.03**, ηp²=0.05 |
|  |  |  |  |  | Emotion: F(1, 145)=0.11, p=0.74, ηp²=0.00 |
|  |  |  |  |  | Group x emotion: F(2, 145)=2.65, p=0.07, ηp²=0.04 |
|  |  |  |  |  | Psychotropics: F(1, 145)=5.79, **p=0.02**, ηp²=0.04 |
| *EBT-Disgust Median Reaction Times*  (FS=50; FMS=49; CC=50) | 780.56  (32.40) | 818.69  (32.72) | 702.59  (32.71) | - | Group: F(2, 145)=3.22, **p=0.04**, ηp²=0.04 |
|  |  |  |  |  | Emotion: F(1, 145)=0.16, p=0.69, ηp²=0.00 |
|  |  |  |  |  | Group x emotion: F(2, 145)=0.17, p=0.84, ηp²=0.00 |
|  |  |  |  |  | Psychotropics: F(1, 145)=5.24, **p=0.02**, ηp²=0.04 |
| *EBT-Sadness Median Reaction Times*  (FS=49; FMS=50; CC=50) | 760.25  (38.17) | 798.90  (31.63) | 674.01  (31.90) | - | Group: F(2, 145)=3.97, **p=0.02**, ηp²=0.05 |
|  |  |  |  |  | Emotion: F(1, 145)=1.02, p=0.31, ηp²=0.01 |
|  |  |  |  |  | Group x emotion: F(2, 145)=0.00, p=1.00, ηp²=0.00 |
|  |  |  |  |  | Psychotropics: F(1, 145)=6.09, **p=0.02**, ηp²=0.04 |
| ***MST Mean Motor Latency (MST MML)*** | **EMM (SE)** | **EMM (SE)** | **EMM (SE)** | **EMM (SE)** | **ANCOVA** |
| *EBT-Anger Median Reaction Times*  (FS=49; FMS=50; CC=50; HC=49) | 795.90  (37.04) | 885.90  (38.25) | 803.69  (37.35) | 803.67  (37.42) | Group: F(3, 193)=1.22, p=0.30, ηp²=0.02 |
|  |  |  |  |  | Emotion: F(1, 193)=0.04, p=0.83, ηp²=0.00 |
|  |  |  |  |  | Group x emotion: F(3, 193)=1.85, p=0.14, ηp²=0.03 |
|  |  |  |  |  | MST MML: F(1, 193)=16.43, **p<0.001**, ηp²=0.08 |
| *EBT-Disgust Median Reaction Times*  (FS=50; FMS=49; CC=50; HC=50) | 775.33  (33.15) | 787.69  (34.57) | 717.34  (33.68) | 742.88  (33.43) | Group: F(3, 194)=0.82, p=0.49, ηp²=0.01 |
|  |  |  |  |  | Emotion: F(1, 194)=4.22, **p=0.04**, ηp²=0.02 |
|  |  |  |  |  | Group x emotion: F(3, 194)=0.31, p=0.82, ηp²=0.01 |
|  |  |  |  |  | MST MML: F(1, 194)=16.70, **p<0.001**, ηp²=0.08 |
| *EBT-Sadness Median Reaction Times*  (FS=49; FMS=50; CC=50; HC=50) | 759.13  (33.09) | 769.06  (34.18) | 686.02  (33.36) | 735.79  (33.10) | Group: F(3, 194)=1.16, p=0.33, ηp²=0.02 |
|  |  |  |  |  | Emotion: F(1, 194)=0.01, p=0.92, ηp²=0.00 |
|  |  |  |  |  | Group x emotion: F(3, 194)=1.04, p=0.38, ηp²=0.02 |
|  |  |  |  |  | MST MML: F(1, 194)=14.33, **p<0.001**, ηp²=0.07 |
| ***RTT Mean Reaction Time (RTT MRT)*** | **EMM (SE)** | **EMM (SE)** | **EMM (SE)** | **EMM (SE)** | **ANCOVA** |
| *EBT-Anger Median Reaction Times*  (FS=49; FMS=50; CC=50; HC=49) | 786.91  (36.93) | 871.86  (38.75) | 805.08  (37.05) | 825.56  (38.12) | Group: F(3, 193)=0.95, p=0.42, ηp²=0.02 |
|  |  |  |  |  | Emotion: F(1, 193)=0.01, p=0.93, ηp²=0.00 |
|  |  |  |  |  | Group x emotion: F(3, 193)=1.80, p=0.15, ηp²=0.03 |
|  |  |  |  |  | RTT MRT: F(1, 193)=19.36, **p<0.001**, ηp²=0.09 |
| *EBT-Disgust Median Reaction Times*  (FS=50; FMS=49; CC=50; HC=50) | 771.79  (33.12) | 773.01  (35.42) | 717.64  (33.55) | 760.50  (34.16) | Group: F(3, 194)=0.57, p=0.63, ηp²=0.01 |
|  |  |  |  |  | Emotion: F(1, 194)=3.29, p=0.07, ηp²=0.02 |
|  |  |  |  |  | Group x emotion: F(3, 194)=0.40, p=0.76, ηp²=0.01 |
|  |  |  |  |  | RTT MRT: F(1, 193)=17.99, **p<0.001**, ηp²=0.09 |
| *EBT-Sadness Median Reaction Times*  (FS=49; FMS=50; CC=50; HC=50) | 750.69  (32.82) | 753.92  (34.43) | 689.01  (32.92) | 756.21  (33.52) | Group: F(3, 194)=0.57, p=0.63, ηp²=0.01 |
|  |  |  |  |  | Emotion: F(1, 194)=1.26, p=0.26, ηp²=0.01 |
|  |  |  |  |  | Group x emotion: F(3, 194)=0.97, p=0.41, ηp²=0.02 |
|  |  |  |  |  | RTT MRT: F(1, 194)=19.55, **p<0.001**, ηp²=0.09 |
| ***RTT Mean Movement Time (RTT MMT)*** | **EMM (SE)** | **EMM (SE)** | **EMM (SE)** | **EMM (SE)** | **ANCOVA** |
| *EBT-Anger Median Reaction Times*  (FS=49; FMS=50; CC=50; HC=49) | 787.61  (38.81) | 918.27  (38.15) | 788.92  (38.36) | 794.02  (38.59) | Group: F(3, 193)=2.81, **p=0.04**, ηp²=0.04 |
|  |  |  |  |  | Emotion: F(1, 193)=0.98, p=0.32, ηp²=0.01 |
|  |  |  |  |  | Group x emotion: F(3, 193)=2.55, p=0.06, ηp²=0.04 |
|  |  |  |  |  | RTT MMT: F(1, 193)=4.56, **p=0.03**, ηp²=0.02 |
| *EBT-Disgust Median Reaction Times*  (FS=50; FMS=49; CC=50; HC=50) | 777.39  (34.88) | 816.88  (35.01) | 699.40  (34.80) | 730.15  (34.71) | Group: F(3, 194)=2.06, p=0.11, ηp²=0.03 |
|  |  |  |  |  | Emotion: F(1, 194)=4.06, **p=0.045**, ηp²=0.02 |
|  |  |  |  |  | Group x emotion: F(3, 194)=0.31, p=0.82, ηp²=0.01 |
|  |  |  |  |  | RTT MMT: F(1, 194)=2.06, p=0.15, ηp²=0.01 |
| *EBT-Sadness Median Reaction Times*  (FS=49; FMS=50; CC=50; HC=50) | 749.28  (34.38) | 793.74  (33.80) | 676.24  (33.95) | 730.55  (33.86) | Group: F(3, 194)=1.98, p=0.12, ηp²=0.03 |
|  |  |  |  |  | Emotion: F(1, 194)=1.49, p=0.22, ηp²=0.01 |
|  |  |  |  |  | Group x emotion: F(3, 194)=1.31, p=0.27, ηp²=0.02 |
|  |  |  |  |  | RTT MMT: F(1, 194)=5.97, **p=0.02**, ηp²=0.03 |

**Key:** ANCOVA=Analysis of Covariance; CC=clinical control; EBT=Emotional Bias Task; EMM=estimated marginal means; FMS=functional motor symptoms; FS=functional seizures; HC=healthy control; M=mean; SD=standard deviation; SE=standard error; WASI-II FSIQ-2=Wechsler Abbreviated Scale of Intelligence – Second edition Full Scale Intelligence Quotient – 2 subtest

*Welch’s ANOVA

**Supplementary Table 22. Emotion Recognition Test sensitivity analyses**

| **Adjustment / Test outcome** | **FS** | **FMS** | **CC** | **HC** | **Test statistics** |
| --- | --- | --- | --- | --- | --- |
| ***MSVT fails excluded*** | **M (SD)** | **M (SD)** | **M (SD)** | **M (SD)** | **ANOVA** |
| *ERT Total Hits*  (FS=47; FMS=46; CC=48; HC=49) | 9.51  (0.20) | 9.67  (0.20) | 10.37  (0.19) | 10.00  (0.19) | ^$^Group: F(3, 186)=3.86, **p=0.01**, ηp²=0.06  ^#^Emotion: F(4.38, 814.47)=175.35, **p<0.001**, ηp²=0.49  ^#^Group x Emotion: F(13.14, 814.47)=0.87, p=0.59, ηp²=0.01 |
| *ERT Unbiased Hits*  (FS=46; FMS=44; CC=47; HC=49) | 0.44  (0.02) | 0.46  (0.02) | 0.51  (0.02) | 0.47  (0.02) | ^$^Group: F(3, 182)=3.94, **p=0.009**, ηp²=0.06  ^#^Emotion: F(4.37, 794.58)=176.98, **p<0.001**, ηp²=0.49  ^#^Group x Emotion: F(13.10, 794.58)=1.46, p=0.13, ηp²=0.02 |
| *ERT False Alarms*  (FS=47; FMS=46; CC=48; HC=49) | 5.49  (0.20) | 5.33  (0.20) | 4.63  (0.19) | 5.00  (0.19) | ^$^Group: F(3, 186)=3.86, **p=0.01**, ηp²=0.06  ^#^Emotion: F(4.33, 806.02)=33.12, **p<0.001**, ηp²=0.15  ^#^Group x Emotion: F(13.00, 806.02)=1.08, p=0.37, ηp²=0.02 |
| *ERT Median Reaction Times*  FS=44; FMS=43; CC=47; HC=47) | 1235.78  (45.64) | 1228.05  (46.17) | 1041.54  (44.16) | 1131.97  (44.16) | ^$^Group: F(3, 177)=4.17, **p=0.007**, ηp²=0.07  ^#^Emotion: F(3.94, 696.84)=64.55, **p<0.001**, ηp²=0.27  ^#^Group x Emotion: F(11.81, 696.84)=1.62, p=0.08, ηp²=0.03 |
| ***Outliers winsorised*** | **M (SD)** | **M (SD)** | **M (SD)** | **M (SD)** | **ANOVA** |
| *ERT Total Hits*  (FS=47; FMS=46; CC=48; HC=49) | 9.52  (0.19) | 9.71  (0.19) | 10.38  (0.19) | 10.00  (0.19) | ^$^Group: F(3, 186)=3.88, **p=0.01**, ηp²=0.06  ^#^Emotion: F(4.26, 791.53)=188.13, **p<0.001**, ηp²=0.50  ^#^Group x Emotion: F(12.77, 791.53)=0.93, p=0.52, ηp²=0.02 |
| *ERT Unbiased Hits*  (FS=46; FMS=44; CC=47; HC=49) | 0.44  (0.02) | 0.46  (0.02) | 0.47  (0.02) | 0.51  (0.02) | ^$^Group: F(3, 182)=4.09, **p=0.008**, ηp²=0.06  ^#^Emotion: F(4.35, 791.66)=181.58, **p<0.001**, ηp²=0.50  ^#^Group x Emotion: F(13.05, 791.66)=1.53, p=0.10, ηp²=0.03 |
| *ERT False Alarms*  (FS=47; FMS=46; CC=48; HC=49) | 5.38  (0.18) | 5.24  (0.18) | 4.48  (0.18) | 4.85  (0.18) | ^$^Group: F(3, 186)=5.14, **p=0.002**, ηp²=0.08  ^#^Emotion: F(4.24, 788.16)=40.95, **p<0.001**, ηp²=0.18  ^#^Group x Emotion: F(12.17, 788.16)=1.14, p=0.32, ηp²=0.02 |
| *ERT Median Reaction Times*  FS=44; FMS=43; CC=47; HC=47) | 1218.09  (39.50) | 1221.51  (39.95) | 1026.43  (38.21) | 1100.87  (38.21) | ^$^Group: F(3, 177)=5.98, **p<0.001**, ηp²=0.09  ^#^Emotion: F(3.55, 627.45)=84.86, **p<0.001**, ηp²=0.32  ^#^Group x Emotion: F(10.64, 627.45)=2.14, **p=0.02**, ηp²=0.04 |
| ***Education*** | **EMM (SE)** | **EMM (SE)** | **EMM (SE)** | **EMM**  **(SE)** | **ANCOVA** |
| *ERT Total Hits*  (FS=49; FMS=50; CC=50; HC=50) | 9.35  (0.23) | 9.45  (0.22) | 10.30  (0.22) | 9.82  (0.22) | Group: F(3, 194)=3.63, **p=0.01**, ηp²=0.05 |
|  |  |  |  |  | ^#^Emotion: F(4.45, 862.52)=21.04, **p<0.001**, ηp²=0.10 |
|  |  |  |  |  | ^#^Group x emotion: F(13.34, 862.52)=1.00, p=0.45, ηp²=0.02 |
|  |  |  |  |  | Education: F(1, 194)=0.98, p=0.32, ηp²=0.01 |
| *ERT Unbiased Hits*  (FS=48; FMS=48; CC=49; HC=50) | 0.43  (0.02) | 0.44  (0.02) | 0.51  (0.02) | 0.46  (0.02) | Group: F(3, 190)=3.80, **p=0.01**, ηp²=0.06 |
|  |  |  |  |  | ^#^Emotion: F(4.35, 826.39)=23.08, **p<0.001**, ηp²=0.11 |
|  |  |  |  |  | ^#^Group x emotion: F(13.05, 826.39)=1.48, p=0.12, ηp²=0.02 |
|  |  |  |  |  | Education: F(1, 190)=0.88, p=0.35, ηp²=0.01 |
| *ERT False Alarms*  (FS=49; FMS=50; CC=50; HC=50) | 5.65  (0.23) | 5.55  (0.22) | 4.70  (0.22) | 5.18  (0.22) | Group: F(3, 194)=3.63, **p=0.01**, ηp²=0.05 |
|  |  |  |  |  | ^#^Emotion: F(4.36, 844.93)=4.00, **p=0.002**, ηp²=0.02 |
|  |  |  |  |  | ^#^Group x emotion: F(13.07, 844.93)=1.08, p=0.37, ηp²=0.02 |
|  |  |  |  |  | Education: F(1, 194)=0.98, p=0.32, ηp²=0.01 |
| *ERT Median Reaction Times*  FS=46; FMS=47; CC=49; HC=47) | 1254.01  (47.33) | 1258.27  (47.41) | 1062.04  (45.91) | 1147.23  (47.31) | Group: F(3, 184)=3.92, **p=0.01**, ηp²=0.06 |
|  |  |  |  |  | ^#^Emotion: F(4.18, 769.70)=3.92, **p=0.003**, ηp²=0.02 |
|  |  |  |  |  | ^#^Group x emotion: F(12.55, 769.70)=1.34, p=0.19, ηp²=0.02 |
|  |  |  |  |  | Education: F(1, 184)=3.46, **p=0.06**, ηp²=0.02 |
| ***Age*** | **EMM (SE)** | **EMM (SE)** | **EMM (SE)** | **EMM**  **(SE)** | **ANCOVA** |
| *ERT Total Hits*  (FS=49; FMS=50; CC=50; HC=50) | 9.32 (0.22) | 9.40 (0.22) | 10.33 (0.22) | 9.87  (0.22) | Group: F(3, 194)=4.51, **p=0.004**, ηp²=0.07 |
|  |  |  |  |  | ^#^Emotion: F(4.47, 867.54)=15.77, **p<0.001**, ηp²=0.08 |
|  |  |  |  |  | ^#^Group x emotion: F(13.42, 867.54)=0.99, p=0.46, ηp²=0.02 |
|  |  |  |  |  | Age: F(1, 194)=2.36, p=0.13, ηp²=0.01 |
| *ERT Unbiased Hits*  (FS=48; FMS=48; CC=49; HC=50) | 0.43 (0.02) | 0.44 (0.02) | 0.51 (0.02) | 0.47  (0.02) | Group: F(3, 190)=4.76, **p=0.003**, ηp²=0.07 |
|  |  |  |  |  | ^#^Emotion: F(4.34, 824.59)=20.80, **p<0.001**, ηp²=0.10 |
|  |  |  |  |  | ^#^Group x emotion: F(13.02, 824.59)=1.49, p=0.11, ηp²=0.02 |
|  |  |  |  |  | Age: F(1, 190)=3.31, p=0.07, ηp²=0.02 |
| *ERT False Alarms*  (FS=49; FMS=50; CC=50; HC=50) | 5.68 (0.22) | 5.60 (0.22) | 4.67 (0.22) | 5.14  (0.22) | Group: F(3, 194)=4.51, **p=0.004**, ηp²=0.07 |
|  |  |  |  |  | ^#^Emotion: F(4.37, 847.97)=10.51, **p<0.001**, ηp²=0.05 |
|  |  |  |  |  | ^#^Group x emotion: F(13.11, 847.97)=1.09, p=0.37, ηp²=0.02 |
|  |  |  |  |  | Age: F(1, 194)=2.36, p=0.13, ηp²=0.01 |
| *ERT Median Reaction Times*  FS=46; FMS=47; CC=49; HC=47) | 1266.56 (44.53) | 1285.48 (44.10) | 1048.52 (43.15) | 1121.85 (44.09) | Group: F(3, 184)=6.83, **p<0.001**, ηp²=0.10 |
|  |  |  |  |  | ^#^Emotion: F(4.16, 766.01)=5.29, **p<0.001**, ηp²=0.03 |
|  |  |  |  |  | ^#^Group x emotion: F(12.49, 766.01)=1.33, p=0.19, ηp²=0.02 |
|  |  |  |  |  | Age: F(1, 184)=25.75, **p<0.001**, ηp²=0.12 |
| ***Gender*** | **EMM (SE)** | **EMM (SE)** | **EMM (SE)** | **EMM**  **(SE)** | **ANCOVA** |
| *ERT Total Hits*  (FS=49; FMS=50; CC=50; HC=50) | 9.32 (0.22) | 9.42 (0.22) | 10.31 (0.22) | 9.86  (0.22) | Group: F(3, 194)=4.22, **p=0.006**, ηp²=0.06 |
|  |  |  |  |  | ^#^Emotion: F(4.45, 863.28)=34.42, **p<0.001**, ηp²=0.15 |
|  |  |  |  |  | ^#^Group x emotion: F(13.35, 863.28)=0.95, p=0.50, ηp²=0.02 |
|  |  |  |  |  | Gender: F(1, 194)=2.75, p=0.10, ηp²=0.01 |
| *ERT Unbiased Hits*  (FS=48; FMS=48; CC=49; HC=50) | 0.43 (0.02) | 0.44 (0.02) | 0.51 (0.02) | 0.47  (0.02) | Group: F(3, 190)=4.34, **p=0.005**, ηp²=0.06 |
|  |  |  |  |  | ^#^Emotion: F(4.36, 828.52)=24.93, **p<0.001**, ηp²=0.12 |
|  |  |  |  |  | ^#^Group x emotion: F(13.08, 828.52)=1.54, p=0.10, ηp²=0.02 |
|  |  |  |  |  | Gender: F(1, 190)=2.13, p=0.15, ηp²=0.01 |
| *ERT False Alarms*  (FS=49; FMS=50; CC=50; HC=50) | 5.68 (0.22) | 5.58 (0.22) | 4.69 (0.22) | 5.14  (0.22) | Group: F(3, 194)=4.22, **p=0.006**, ηp²=0.06 |
|  |  |  |  |  | ^#^Emotion: F(4.39, 850.63)=6.45, **p<0.001**, ηp²=0.03 |
|  |  |  |  |  | ^#^Group x emotion: F(13.15, 850.63)=1.12, p=0.34, ηp²=0.02 |
|  |  |  |  |  | Gender: F(1, 194)=2.75, p=0.10, ηp²=0.01 |
| *ERT Median Reaction Times*  FS=46; FMS=47; CC=49; HC=47) |  |  |  |  | Group: F(3, 184)=5.23, **p=0.002**, ηp²=0.08 |
|  |  |  |  |  | ^#^Emotion: F(4.16, 765.06)=10.54, **p<0.001**, ηp²=0.05 |
|  |  |  |  |  | ^#^Group x emotion: F(12.47, 765.06)=1.35, p=0.18, ηp²=0.02 |
|  |  |  |  |  | Gender: F(1, 184)=4.83, **p=0.03**, ηp²=0.03 |
| ***WASI-II FSIQ-2*** | **EMM (SE)** | **EMM (SE)** | **EMM (SE)** | **EMM**  **(SE)** | **ANCOVA** |
| *ERT Total Hits*  (FS=49; FMS=50; CC=50; HC=50) | 9.54  (0.22) | 9.59  (0.21) | 10.08  (0.21) | 9.71  (0.21) | Group: F(3, 194)=1.21, p=0.31, ηp²=0.02 |
|  |  |  |  |  | ^#^Emotion: F(4.46, 864.59)=5.18, **p<0.001**, ηp²=0.03 |
|  |  |  |  |  | ^#^Group x emotion: F(13.37, 864.59)=0.84, p=0.63, ηp²=0.01 |
|  |  |  |  |  | WASI-II FSIQ-2: F(1, 194)=25.91, **p<0001**, ηp²=0.12 |
| *ERT Unbiased Hits*  (FS=48; FMS=48; CC=49; HC=50) | 0.44  (0.02) | 0.45  (0.02) | 0.49  (0.02) | 0.46  (0.02) | Group: F(3, 190)=1.37, p=0.26, ηp²=0.02 |
|  |  |  |  |  | ^#^Emotion: F(4.34, 824.14)=7.02, **p<0.001**, ηp²=0.04 |
|  |  |  |  |  | ^#^Group x emotion: F(13.01, 824.14)=1.35, p=0.18, ηp²=0.02 |
|  |  |  |  |  | WASI-II FSIQ-2: F(1, 190)=24.56, **p<0.001**, ηp²=0.11 |
| *ERT False Alarms*  (FS=49; FMS=50; CC=50; HC=50) | 5.46  (0.22) | 5.41  (0.21) | 4.92  (0.21) | 5.29  (0.21) | Group: F(3, 194)=1.21, p=0.31, ηp²=0.02 |
|  |  |  |  |  | ^#^Emotion: F(4.37, 847.13)=3.13, **p=0.01**, ηp²=0.02 |
|  |  |  |  |  | ^#^Group x emotion: F(13.10, 847.13)=1.05, p=0.40, ηp²=0.02 |
|  |  |  |  |  | WASI-II FSIQ-2: F(1, 194)=25.91, **p<0.001**, ηp²=0.12 |
| *ERT Median Reaction Times*  FS=44; FMS=43; CC=47; HC=47) | 1235.46  (47.22) | 1244.87 (46.89) | 1084.49 (46.11) | 1155.40 (46.50) | Group: F(3, 184)=2.39, p=0.07, ηp²=0.04 |
|  |  |  |  |  | ^#^Emotion: F(4.17, 767.55)=1.81, p=0.12, ηp²=0.01 |
|  |  |  |  |  | ^#^Group x emotion: F(12.51, 767.55)=1.25, p=0.24, ηp²=0.02 |
|  |  |  |  |  | WASI-II FSIQ-2: F(1, 184)=9.40, **p=0.003**, ηp²=0.04 |
| ***Psychotropic medications (FS/FMS/CC)*** | **EMM (SE)** | **EMM (SE)** | **EMM (SE)** |  | **ANCOVA** |
| *ERT Total Hits*  (FS=49; FMS=50; CC=50) | 9.35  (0.23) | 9.44  (0.23) | 10.28  (0.23) | - | Group: F(2, 145)=4.85, **p=0.009**, ηp²=0.06 |
|  |  |  |  |  | ^#^Emotion: F(4.35, 631.30)=15.74, **p<0.001**, ηp²=0.10 |
|  |  |  |  |  | ^#^Group x emotion: F(8.71, 631.30)=1.07, p=0.38, ηp²=0.02 |
|  |  |  |  |  | Psychotropics: F(1, 145)=1.81, p=0.18, ηp²=0.01 |
| *ERT Unbiased Hits*  (FS=48; FMS=48; CC=49) | 0.43  (0.02) | 0.44  (0.02) | 0.50  (0.02) | - | Group: F(2, 141)=5.27, **p=0.006**, ηp²=0.07 |
|  |  |  |  |  | ^#^Emotion: F(4.38, 617.41)=17.44, **p<0.001**, ηp²=0.11 |
|  |  |  |  |  | ^#^Group x emotion: F(8.76, 617.41)=0.52, p=0.85, ηp²=0.01 |
|  |  |  |  |  | Psychotropics: F(1, 141)=1.15, p=0.29, ηp²=0.01 |
| *ERT False Alarms*  (FS=49; FMS=50; CC=50) | 5.65  (0.23) | 5.56  (0.23) | 4.72  (0.23) | - | Group: F(2, 145)=4.85, **p=0.009**, ηp²=0.06 |
|  |  |  |  |  | ^#^Emotion: F(4.43, 642.32)=2.87, **p=0.02**, ηp²=0.02 |
|  |  |  |  |  | ^#^Group x emotion: F(8.86, 642.32)=0.75, p=0.66, ηp²=0.01 |
|  |  |  |  |  | Psychotropics: F(1, 144)=1.81, p=0.18, ηp²=0.01 |
| *ERT Median Reaction Times*  FS=46; FMS=47; CC=49) | 1254.70  (48.02) | 1268.19  (47.50) | 1066.53  (46.98) | - | Group: F(2, 138)=5.59, **p=0.005**, ηp²=0.08 |
|  |  |  |  |  | ^#^Emotion: F(3.96, 545.93)=8.40, **p<0.001**, ηp²=0.06 |
|  |  |  |  |  | ^#^Group x emotion: F(7.91, 545.93)=1.82, p=0.07, ηp²=0.03 |
|  |  |  |  |  | Psychotropics: F(1, 138)=3.47, p=0.07, ηp²=0.03 |
| ***MST Mean Motor Latency (MST MML)*** | **EMM (SE)** | **EMM (SE)** | **EMM (SE)** | **EMM**  **(SE)** | **ANCOVA** |
| *ERT Total Hits*  (FS=49; FMS=50; CC=50; HC=50) | 9.36  (0.22) | 9.61  (0.23) | 10.20  (0.22) | 9.75  (0.22) | Group: F(3, 194)=2.47, p=0.06, ηp²=0.04 |
|  |  |  |  |  | ^#^Emotion: F(4.45, 862.24)=15.80, **p<0.001**, ηp²=0.08 |
|  |  |  |  |  | ^#^Group x emotion: F(13.33, 862.24)=0.99, p=0.46, ηp²=0.02 |
|  |  |  |  |  | MST MML: F(1, 194)=8.90, **p=0.003**, ηp²=0.04 |
| *ERT Unbiased Hits*  (FS=48; FMS=48; CC=49; HC=50) | 0.43  (0.02) | 0.45  (0.02) | 0.50  (0.02) | 0.46  (0.02) | Group: F(3, 190)=2.76, **p=0.04**, ηp²=0.04 |
|  |  |  |  |  | ^#^Emotion: F(4.35, 826.58)=19.66, **p<0.001**, ηp²=0.09 |
|  |  |  |  |  | ^#^Group x emotion: F(13.05, 826.58)=1.42, p=0.15, ηp²=0.02 |
|  |  |  |  |  | MST MML: F(1, 190)=7.67, **p=0.006**, ηp²=0.04 |
| *ERT False Alarms*  (FS=49; FMS=50; CC=50; HC=50) | 5.64  (0.22) | 5.40  (0.23) | 4.80  (0.22) | 5.25  (0.22) | Group: F(3, 194)=2.47, p=0.06, ηp²=0.04 |
|  |  |  |  |  | ^#^Emotion: F(4.36, 844.86)=3.31, **p=0.009**, ηp²=0.02 |
|  |  |  |  |  | ^#^Group x emotion: F(13.07, 844.86)=1.14, p=0.32, ηp²=0.02 |
|  |  |  |  |  | MST MML: F(1, 194)=8.90, **p=0.003**, ηp²=0.04 |
| *ERT Median Reaction Times*  FS=46; FMS=47; CC=49; HC=47) | 1249.33  (45.27) | 1219.57  (46.37) | 1092.17  (44.65) | 1159.11  (45.10) | Group: F(3, 184)=2.27, p=0.08, ηp²=0.04 |
|  |  |  |  |  | ^#^Emotion: F(4.17, 766.75)=5.66, **p<0.001**, ηp²=0.03 |
|  |  |  |  |  | ^#^Group x emotion: F(12.50, 766.75)=1.41, p=0.15, ηp²=0.02 |
|  |  |  |  |  | MST MML: F(1, 184)=19.79, **p<0.001**, ηp²=0.10 |
| ***RTT Mean Reaction Time (RTT MRT)*** | **EMM (SE)** | **EMM (SE)** | **EMM (SE)** | **EMM**  **(SE)** | **ANCOVA** |
| *ERT Total Hits*  (FS=49; FMS=50; CC=50; HC=50) | 9.47  (0.20) | 9.90  (0.21) | 10.07  (0.20) | 9.48  (0.21) | Group: F(3, 194)=2.26, p=0.08, ηp²=0.03 |
|  |  |  |  |  | ^#^Emotion: F(4.45, 863.71)=4.95, **p<0.001**, ηp²=0.03 |
|  |  |  |  |  | ^#^Group x emotion: F(13.36, 863.71)=1.02, p=0.43, ηp²=0.02 |
|  |  |  |  |  | RTT MRT: F(1, 194)=45.08, **p<0.001**, ηp²=0.19 |
| *ERT Unbiased Hits*  (FS=48; FMS=48; CC=49; HC=50) | 0.44  (0.02) | 0.47  (0.02) | 0.49  (0.02) | 0.44  (0.02) | Group: F(3, 190)=2.77, **p=0.04**, ηp²=0.04 |
|  |  |  |  |  | ^#^Emotion: F(4.36, 828.27)=6.64, **p<0.001**, ηp²=0.03 |
|  |  |  |  |  | ^#^Group x emotion: F(13.08, 828.27)=1.39, p=0.16, ηp²=0.02 |
|  |  |  |  |  | RTT MRT: F(1, 190)=41.90, **p<0.001**, ηp²=0.18 |
| *ERT False Alarms*  (FS=49; FMS=50; CC=50; HC=50) | 5.53  (0.20) | 5.10  (0.21) | 4.93  (0.20) | 5.52  (0.21) | Group: F(3, 194)=2.26, p=0.08, ηp²=0.03 |
|  |  |  |  |  | ^#^Emotion: F(4.35, 844.19)=1.68, p=0.15, ηp²=0.01 |
|  |  |  |  |  | ^#^Group x emotion: F(13.05, 844.19)=1.27, p=0.23, ηp²=0.02 |
|  |  |  |  |  | RTT MRT: F(1, 194)=45.08, **p<0.001**, ηp²=0.19 |
| *ERT Median Reaction Times*  FS=46; FMS=47; CC=49; HC=47) | 1236.27  (44.07) | 1183.22  (46.23) | 1099.41  (43.25) | 1200.69  (45.00) | Group: F(3, 184)=1.79, p=0.15, ηp²=0.03 |
|  |  |  |  |  | ^#^Emotion: F(4.22, 777.11)=10.85, **p<0.001**, ηp²=0.06 |
|  |  |  |  |  | ^#^Group x emotion: F(12.67, 777.11)=1.97, **p=0.02**, ηp²=0.03 |
|  |  |  |  |  | RTT MRT: F(1, 184)=32.48, **p<0.001**, ηp²=0.15 |
| ***RTT Mean Movement Time (RTT MMT)*** | **EMM (SE)** | **EMM (SE)** | **EMM (SE)** | **EMM**  **(SE)** | **ANCOVA** |
| *ERT Total Hits*  (FS=49; FMS=50; CC=50; HC=50) | 9.43  (0.23) | 9.49  (0.22) | 10.23  (0.22) | 9.77  (0.22) | Group: F(3, 194)=2.55, p=0.06, ηp²=0.04 |
|  |  |  |  |  | ^#^Emotion: F(4.45, 862.93)=9.95, **p<0.001**, ηp²=0.05 |
|  |  |  |  |  | ^#^Group x emotion: F(13.34, 862.93)=0.92, p=0.54, ηp²=0.01 |
|  |  |  |  |  | RTT MMT: F(1, 194)=5.59, **p=0.02**, ηp²=0.03 |
| *ERT Unbiased Hits*  (FS=48; FMS=48; CC=49; HC=50) | 0.44  (0.02) | 0.45  (0.02) | 0.50  (0.02) | 0.46  (0.02) | Group: F(3, 190)=2.47, p=0.06, ηp²=0.04 |
|  |  |  |  |  | ^#^Emotion: F(4.36, 828.99)=5.98, **p<0.001**, ηp²=0.03 |
|  |  |  |  |  | ^#^Group x emotion: F(13.09, 828.99)=1.53, p=1.00, ηp²=0.02 |
|  |  |  |  |  | RTT MMT: F(1, 190)=9.04, **p=0.003**, ηp²=0.05 |
| *ERT False Alarms*  (FS=49; FMS=50; CC=50; HC=50) | 5.57  (0.23) | 5.51  (0.22) | 4.77  (0.22) | 5.23  (0.22) | Group: F(3, 194)=2.55, p=0.06, ηp²=0.04 |
|  |  |  |  |  | ^#^Emotion: F(4.36, 845.60)=3.57, **p=0.005**, ηp²=0.02 |
|  |  |  |  |  | ^#^Group x emotion: F(13.08, 845.60)=1.17, p=0.29, ηp²=0.02 |
|  |  |  |  |  | RTT MMT: F(1, 194)=5.59, **p=0.02**, ηp²=0.03 |
| *ERT Median Reaction Times*  FS=46; FMS=47; CC=49; HC=47) | 1237.60  (46.63) | 1241.68  (46.54) | 1083.74  (45.51) | 1157.27  (46.14) | Group: F(3, 184)=2.48, p=0.06, ηp²=0.04 |
|  |  |  |  |  | ^#^Emotion: F(4.17, 768.00)=4.11, **p=0.002**, ηp²=0.02 |
|  |  |  |  |  | ^#^Group x emotion: F(12.52, 768.00)=1.23, p=0.26, ηp²=0.02 |
|  |  |  |  |  | RTT MMT: F(1, 184)=12.04, **p<0.001**, ηp²=0.06 |

**Key:** ANCOVA=Analysis of Covariance; CC=clinical control; ERT=Emotion Recognition Test; EMM=estimated marginal means; FMS=functional motor symptoms; FS=functional seizures; HC=healthy control; SE=standard error; WASI-II FSIQ-2=Wechsler Abbreviated Scale of Intelligence – Second edition Full Scale Intelligence Quotient – 2 subtest

^$^Mixed ANOVA; ^#^Greenhouse-Geiser correction (sphericity assumption violated)

**Supplementary Table 23. Metacognitive performance ratings**

| **Test** | **FS**  **(n=50)** | **FMS**  **(n=50)** | **CC**  **(n=50)** | **HC**  **(n=50)** | **ANOVA statistics** |
| --- | --- | --- | --- | --- | --- |
| ***Medical Symptom Validity Test: M (SD)*** | 5.13 (1.08) | 5.16 (1.13) | 5.04 (1.09) | 5.18 (1.04) | F(3, 194)=0.16, p=0.92, η²=0.00 |
| ***WASI-II Vocabulary:***  ***M (SD)*** | 4.48 (0.79) | 4.34 (0.98) | 4.80 (0.93) | 4.50 (0.86) | F(3, 196)=2.35, p=0.07, η²=0.04 |
| ***WASI-II Matrix Reasoning: M (SD)*** | 3.74 (1.14) | 3.62 (1.16) | 3.92 (1.01) | 4.14 (0.99) | F(3, 196)=2.21, p=0.09, η²=0.03 |
| ***Motor Screening Test:***  ***M (SD)*** | 5.52 (1.11) | 5.62 (1.21) | 5.12 (1.27) | 5.32 (1.19) | F(3, 196)=1.72, p=0.17, ω²=0.03 |
| ***Reaction Time Test:***  ***M (SD)*** *FS=49^&^* | 4.74 (1.17) | 4.80 (1.40) | 4.72 (1.14) | 5.12 (1.12) | F(3, 195)=1.19, p=0.31, η²=0.02 |
| ***Rapid Visual Information Processing (RVIP): M (SD)***  *FS=47; CC/HC=49^&^* | 2.94 (1.05) | 2.40 (1.14) | 3.53 (1.23) | 3.88 (1.18) | F(3, 192)=15.79, **p<0.001**, η²=0.20 |
| ***Spatial Span Forward:***  ***M (SD)*** *HC=49^&^* | 3.18 (1.08) | 3.36 (1.22) | 3.36 (1.08) | 3.57 (1.19) | F(3, 195)=0.96, p=0.41, η²=0.02 |
| ***Spatial Span Reverse:***  ***M (SD)*** | 3.36 (1.34) | 2.96 (1.31) | 3.26 (1.17) | 3.54 (1.13) | F(3, 196)=1.92, p=0.13, η²=0.03 |
| ***Intra-Extra Dimensional Set Shift (IEDSS): M (SD)*** | 3.92 (1.58) | 4.04 (1.36) | 4.56 (0.91) | 4.44 (1.11) | F(3, 196)=2.98, p<0.03^!^, η²=0.04 |
| ***Stop Signal Task (SST):***  ***M (SD)*** *FS/FMS=49^&^* | 3.53 (1.06) | 3.50 (1.14) | 3.48 (1.00) | 3.94 (1.06) | F(3, 194)=2.15, p=0.10, η²=0.03 |
| ***Emotional Bias Task – Anger: M (SD)*** *FS=49^&^* | 4.67 (1.13) | 4.48 (1.33) | 4.56 (0.95) | 4.46 (0.86) | *F(3, 107.07)=0.41, p=0.75, ω²=0.01 |
| ***Emotional Bias Task – Disgust: M (SD)*** *FS=49^&^* | 4.18 (1.17) | 4.34 (1.24) | 4.52 (0.71) | 4.34 (0.92) | *F(3, 105.58)=1.14, p=0.34, ω²=0.00 |
| ***Emotional Bias Task – Sadness: M (SD)*** *FS=49^&^* | 4.18 (1.01) | 4.46 (1.15) | 4.46 (0.76) | 4.44 (0.94) | F(3, 195)=0.99, p=0.40, η²=0.02 |
| ***Emotion Recognition Test: M (SD)*** *FS=49^&^* | 3.53 (1.12) | 3.62 (1.10) | 3.88 (0.80) | 3.90 (1.04) | *F(3, 107.10)=1.61, p=0.19, ω²=0.01 |

**Key:** CC=clinical controls; FS=functional seizures; FMS=functional motor symptoms; HC=healthy controls; M=mean; SD=standard deviation ^&^Sample size deviated due to missing data *Welch’s ANOVA ^!^Did not survive Benjamini-Hochberg correction (5%)

**Supplementary Table 24. RVIP performance ratings sensitivity analyses**

| **Adjustment** | **FS** | **FMS** | **CC** | **HC** | **Test statistics** |
| --- | --- | --- | --- | --- | --- |
| ***MSVT fails excluded*** | **M (SD)**  **n=46** | **M (SD)**  **n=46** | **M (SD)**  **n=47** | **M (SD)**  **n=48** | **ANOVA** |
|  | 2.98  (1.02) | 2.46  (1.15) | 3.60  (1.20) | 3.88  (1.20) | F(3, 183)=14.47, **p<0.001**, η²=0.19 |
| ***Outliers winsorised*** | **M (SD)**  **n=46** | **M (SD)**  **n=46** | **M (SD)**  **n=47** | **M (SD)**  **n=48** | **ANOVA** |
|  | 3.00  (1.02) | 2.46  (1.15) | 3.57  (1.14) | 3.813 (1.10) | F(3, 183)=14.28, **p<0.001**, η²=0.19 |
| ***Education*** | **EMM (SE)**  **n=47** | **EMM (SE)**  **n=50** | **EMM (SE)**  **n=49** | **EMM**  **(SE)**  **n=49** | **ANCOVA** |
|  | 2.97  (0.17) | 2.46  (0.17) | 3.49  (0.17) | 3.82  (0.17) | Group: F(3, 190)=12.43, **p<0.001**, ηp²=0.16 |
|  |  |  |  |  | Education: F(1, 190)=4.36, **p=0.04**, ηp²=0.02 |
| ***Age*** | **EMM (SE)**  **n=47** | **EMM (SE)**  **n=50** | **EMM (SE)**  **n=49** | **EMM**  **(SE)**  **n=49** | **ANCOVA** |
|  | 2.94 (0.17) | 2.40 (0.16) | 3.53 (0.17) | 3.88 (0.17) | Group: F(3, 190)=15.80, **p<0.001**, ηp²=0.20 |
|  |  |  |  |  | Age: F(1, 190)=0.24, p=0.63, ηp²=0.001 |
| ***Gender*** | **EMM (SE)**  **n=47** | **EMM (SE)**  **n=50** | **EMM (SE)**  **n=49** | **EMM**  **(SE)**  **n=49** | **ANCOVA** |
|  | 2.93 (0.17) | 2.40 (0.16) | 3.53 (0.17) | 3.88 (0.17) | Group: F(3, 190)=15.71, **p<0.001**, ηp²=0.20 |
|  |  |  |  |  | Gender: F(1, 190)=0.05, p=0.83, ηp²=0.00 |
| ***WASI-II FSIQ-2*** | **EMM (SE)**  **n=47** | **EMM (SE)**  **n=50** | **EMM (SE)**  **n=49** | **EMM**  **(SE)**  **n=49** | **ANCOVA** |
|  | 3.04  (0.17) | 2.49  (0.16) | 3.41 (0.17) | 3.81  (0.16) | Group: F(3, 190)=11.42, **p<0.001**, ηp²=0.15 |
|  |  |  |  |  | WASI-II FSIQ-2: F(1, 190)=9.40, **p=0.002**, ηp²=0.05 |
| ***Psychotropic medication*** | **EMM (SE)**  **n=47** | **EMM (SE)**  **n=50** | **EMM (SE)**  **n=49** |  | **ANCOVA** |
|  | 2.96  (0.17) | 2.43  (0.16) | 3.47  (0.16) | - | Group: F(2, 142)=10.19, **p<0.001**, ηp²=0.13 |
|  |  |  |  |  | Psychotropics: F(1, 142)=5.54, **p=0.02**, ηp²=0.04 |
| ***RVIP Ability*** | **EMM (SE)**  **n=49** | **EMM (SE)**  **n=48** | **EMM (SE)**  **n=50** | **EMM**  **(SE)**  **n=50** |  |
|  | 3.11  (0.16) | 2.69  (0.16) | 3.37  (0.16) | 3.75  (0.16) | Group: F(3, 182)=7.35, **p<0.001**, ηp²=0.11 |
|  |  |  |  |  | RVIP Ability: F(1, 182)=30.34, **p<0.001**, ηp²=0.14 |

**Key:** ANCOVA=Analysis of Covariance; CC=clinical control; EMM=estimated marginal means; FMS=functional motor symptoms; FS=functional seizures; HC=healthy control; M=mean; RVIP=Rapid Visual Information Processing; SD=standard deviation; SE=standard error; WASI-II FSIQ-2=Wechsler Abbreviated Scale of Intelligence – Second edition Full Scale Intelligence Quotient – 2 subtest

**Supplementary Table 25. RVIP performance ratings**

**post-hoc tests**

| **Comparison** | **Mean difference (standard error)** | **Confidence interval (95%)** | **p-value** |
| --- | --- | --- | --- |
| FS vs FMS | 0.54 (0.23) | -.09, 1.16 | 0.14 |
| FS vs HC | -0.94 (0.24) | -1.57, -0.31 | **<0.001** |
| FS vs CC | -0.59 (0.24) | -1.22, 0.03 | 0.08 |
| FMS vs HC | -1.48 (0.23) | -2.10, -0.86 | **<0.001** |
| FMS vs CC | -1.13 (0.23) | -1.75, -0.51 | **<0.001** |
| HC vs CC | 0.35 (0.23) | -0.27, 0.97 | 0.83 |

**Key:** CC=clinical controls; FMS=functional motor symptoms;

FS=functional seizures; HC=healthy controls
